# Identification of the Antioxidant Enzyme B166 as a Novel Biomarker for Patients With Glioblastoma Multiforme

**DOI:** 10.1155/ancp/6120748

**Published:** 2026-02-17

**Authors:** Weiqiang Liu, Bingwu Wang, Fanhua Kong, Cindy Cheung

**Affiliations:** ^1^ Department of Surgery, Weifang People’s Hospital, The First Affiliated Hospital of Shandong Second Medical University, Weifang, 261000, Shandong, China, wfph.cn; ^2^ Department of Pathology, Weifang People’s Hospital, The First Affiliated Hospital of Shandong Second Medical University, Weifang, 261000, Shandong, China, wfph.cn; ^3^ Department of Oncology, Mayo Clinic, Rochester, 55905, Minnesota, USA, mayo.edu; ^4^ Department of Oncology, Weifang People’s Hospital, The First Affiliated Hospital of Shandong Second Medical University, Weifang, 261000, Shandong, China, wfph.cn

**Keywords:** B166, glioblastoma multiforme, lipid metabolism, reactive oxygen species, sterol regulatory element-binding protein 1

## Abstract

This study aimed to explore the regulatory role of sterol regulatory element‐binding protein 1 (SREBP1) in the antioxidant enzyme B166 and to examine the prognostic and therapeutic potential of B166 in glioblastoma multiforme (GBM). RNA‐seq data from TCGA and GTEX databases were analyzed to explore the relationship between B166 and GBM, as well as between B166 and SREBP1. Functional experiments were performed to determine whether SREBPs could regulate B166 expression level and to identify the major regulator of B166. Immunofluorescence staining was performed to investigate the cellular distribution of B166 in GBM. Quantitative reverse transcription polymerase chain reaction was employed to determine the regulatory effects of SREBP1‐a. CellROX Deep Red dye was applied to detect mitochondrial reactive oxygen species (ROS) levels. The results revealed that B166 protein was significantly upregulated in GBM tissues compared with lower‐grade gliomas and normal brain tissues (*p*<0.0001). The B166 expression level was elevated through SREBP1‐a‐mediated regulation in GBM, simultaneously decreasing endogenous ROS levels to maintain cellular redox homeostasis and preserve normal mitochondrial function. Reducing SREBP1 level decreased both RNA and protein expression levels of B166. Overexpression of the active form of SREBP1‐a elevated the protein levels of B166 in both the mitochondria and nucleus. Pharmacological suppression of SREBP1 or genetic inhibition of B166 disrupted redox homeostasis, resulting in elevated oxidative stress and rapid cell death. In conclusion, B166 may serve as a promising therapeutic target for GBM. Its overexpression could enhance ROS elimination, highlighting its potential therapeutic benefits for GBM patients.

## 1. Background

With a median overall survival (OS) of only 12–15 months, glioblastoma multiforme (GBM) is recognized as the most aggressive primary brain tumor globally [1]. Despite advancements in clinical management through the combination of temozolomide with radiotherapy, GBM patients’ prognosis has been slightly improved [2]. Clinical studies demonstrated that targeted therapies did not significantly improve GBM patients’ survival outcomes [3, 4]. Therefore, it is imperative to investigate the underlying mechanisms of GBM progression and to develop more effective therapeutic approaches.

Over the past few decades, it has been evidenced that GBM progression is associated with reprograming of lipid metabolism, with an upsurge in lipogenesis and a predilection for mitochondrial fatty acid oxidation. To support the high demands of membrane expansion, signal transduction, and other physiological activities, rapidly dividing GBM cells consume cholesterol at rates that exceed their requirements [5]. Both cholesterol uptake and de novo synthesis pathways are regulated by sterol regulatory element‐binding proteins (SREBPs), which exist in three isoforms: SREBP1‐a, SREBP1‐c, and SREBP2. Sterol regulatory element‐binding protein 1 (SREBP1) is a major transcription factor for several important lipogenic enzymes, including fatty acid synthase (FASN), acetyl‐CoA carboxylases (ACC), and stearoyl‐CoA desaturase 1 (SCD1), playing a crucial role in fatty acid synthesis and cholesterol synthesis. Notably, SREBP1 is markedly upregulated in GBM patients and is closely associated with poor patient outcomes, making it a pivotal factor in GBM pathogenesis [6–9]. However, the mechanisms by which SREBP1, a key transcriptional regulator of lipid metabolism, contributes to GBM pathogenesis remain elusive.

Metabolic reprograming in GBM also leads to alterations in energy production and redox balance. GBM cells upregulate surrogate systems, such as acyl‐CoA‐binding protein, to compensate for impaired tricarboxylic acid cycle function and shift to fatty acids as the primary catabolic pathway, thereby producing ATP more efficiently [6, 10]. During this process, excessive fatty acids are transported into the mitochondria for oxidation and phosphorylation, producing reactive oxygen species (ROS), which can cause severe damage to cell membranes, chromosomal DNA, and proteins, ultimately leading to cell death. To counteract these harmful effects, GBM cells increase the production of certain antioxidant molecules to maintain tolerable and beneficial levels of endogenous ROS, supporting cell survival and proliferation. The thiol‐based antioxidant system consists of mitochondrial peroxiredoxin (PRX), superoxide dismutase, and glutathione peroxidase. Superoxide dismutase converts the superoxide anion into hydrogen peroxide, which is then further reduced to water and other nonreactive molecules by PRX or glutathione peroxidase [11–13].

Among these systems, PRXs are regarded as the most important ROS scavengers in cancer, promoting the reduction of hydroperoxides, including hydrogen peroxide, lipid peroxide, peptide/protein hydroperoxide, and other organic peroxides using thioredoxins as reducing agents [14, 15]. B166, also known as PRX5, is significantly upregulated in aggressive Hodgkin’s lymphoma, malignant mesothelioma, ovarian cancer, and breast cancer (BRCA) [16–19]. In BRCA, high‐B166 expression level is associated with a poor prognosis; in aggressive Hodgkin lymphoma, B166 expression level also indicates a low likelihood of complete response to chemotherapy [16, 17]. Despite its confirmed roles in other malignancies, the regulation and functional significance of B166 in GBM remain largely unexplored. A previous study demonstrated that the mitochondrial form of B166 has a signal peptide at its N‐terminus, which is cleaved after guiding its entrance into the mitochondria, and it contains two important Cystine residues (Cys100 and Cys204), catalyzing the reduction of hydroperoxides [20], highlighting its potential role in maintaining redox homeostasis. Therefore, it is hypothesized that different B166 isoforms may also contribute to GBM progression, which has not been explored in previous studies. Moreover, SREBP1 has been reported to regulate antioxidant pathways in other cancer types [21]; however, whether it modulates B166 in GBM has not been investigated. These gaps highlight the need to explore B166 isoforms in GBM and their potential regulation by SREBP1. Therefore, this study aimed to investigate the role of B166 in GBM, explore its potential regulation by SREBP1, and evaluate its prognostic and therapeutic potential for GBM.

## 2. Methods

### 2.1. Cell Culture and Reagents

Authenticated human GBM cell lines U251 and U87 were obtained from the Cell Resource Center, Institute of Basic Medical Sciences, Peking Union Medical College, Institute of Basic Medical Sciences, Chinese Academy of Medical Sciences. The cell lines were cultured in a Dulbecco’s modified Eagle’s medium (DMEM) supplemented with 5% fetal bovine serum (HyClone, Logan, UT, USA). All cells were cultured in a humidified atmosphere with 5% CO_2_ at 37°C.

Antibodies against B166 (17724‐1‐AP), glucose‐6‐phosphate dehydrogenase (G6PD) (25413‐1‐AP), and SREBP1 (14088‐1‐AP) were obtained from Proteintech (Rosemont, IL, USA). Cell Signaling Technology (Danvers, MA, USA) provided the antibodies for FASN (#3180), stearoyl‐CoA desaturase (SCD) (#2438), and HA‐tag (#C29F4). Paraformaldehyde (P6148), Triton X‐100 (T8787), the antibodies for β‐actin (#A1978) and Flag‐tag (#F3165), lentiviral vectors containing short hairpin RNAs (shSREBP1‐1#, TRCN0000414192; shSREBP1‐2#, TRCN0000421299), and the nonmammalian control (shCTR, SHC002) were purchased from Sigma–Aldrich (St. Louis, MO, USA). Alexa Fluor 488 goat anti‐rabbit IgG (A‐11034), Alexa Fluor 568 goat anti‐rabbit IgG (A‐11036), and Lipofectamine RNAiMAX (13778) antibodies were purchased from Life Technologies (Carlsbad, CA, USA). X‐treme GENE HP DNA transfection reagent (06366236001), small interfering RNAs (siRNAs), and drugs (i.e., fatostatin and PF429242) were provided by Roche, Dharmacon, and MedChemExpress, respectively. GenScript Co., Ltd. (Nanjing, China) provided 2× HA‐tagged SREBP2‐N, plasmids pcDNA3.0‐B166‐V5‐3× Flag, and pcDNA3.0 (empty vector). Plasmids encoding 2× Flag‐tagged SREBP1‐aN, SREBP1‐cN, and SREBP2‐N (pcDNA3.1) were purchased from Addgene (cat. #26801, #26802, and #26807), along with the psPAX2 packaging plasmid (cat. #12260) and the pMD2.G envelope plasmid (cat. #12259). Adenoviruses containing SREBP1‐aN, SREBP1‐cN, and SREBP2‐N were prepared by Shanghai GeneChem Co., Ltd. (Shanghai, China). Scramble nonsilencing siRNAs, which were purchased from GenePharma (Shanghai GenePharma Co., Ltd., Shanghai, China), were used as negative controls.

### 2.2. Microarray of Glioma Specimens

Glioma tissue microarray (TMA) cohort PHG‐1, comprising low‐grade (WHO Grade I, *n* = 14; Grade II, *n* = 36; Grade III, *n* = 26) and high‐grade gliomas (GBM, WHO Grade IV, *n* = 12), was supplied by Superchip Biotech (Cat. Number HBraG180Su01, Table S1) [22]. Approval from the Institutional Review Board of Superchip Biotech was obtained before commencing the study. Informed consent was granted by all patients before the use of their clinical specimens for research purposes. The study was also approved by the Institutional Review Board of the First Affiliated Hospital, Shandong Second Medical University (Jinan, China; Approval Number KYLL20240219‐2). The histology of the specimens was centrally reviewed and confirmed by two experienced neuropathologists using hematoxylin and eosin staining, according to the 2021 World Health Organization (WHO) classification of Central Nervous System Tumors [23].

### 2.3. Immunohistochemistry (IHC) of TMA

Paraffin‐embedded tissue sections were prepared using standard methods, and immunoperoxidase staining was used to detect the expression levels of B166 (1:700 dilution), G6PD (1:1000 dilution), and SREBP1 (1:2000 dilution). The IHC protocol was identical to that previously published [23]. The expression levels of B166, G6PD, and SREBP1 were independently analyzed by two neuropathologists who were unaware of the experimental design and patient data. Each specimen was scored based on immunostaining intensity, and *H*‐scores were accordingly calculated.

### 2.4. Western Blotting

Cells were lysed with RIPA buffer containing a protease inhibitor cocktail and a phosphatase inhibitor. Proteins were separated by sodium dodecyl‐sulfate polyacrylamide gel electrophoresis (SDS‐PAGE) and transferred onto enhanced chemiluminescence (ECL) nitrocellulose membranes. Following 60‐min incubation with 5% nonfat milk (diluted with Tris‐buffered saline containing 0.1% Tween 20), the membranes were probed with a series of primary antibodies, which were subsequently detected using horseradish peroxidase‐conjugated secondary antibodies. The immunoreactivity was visualized using an ECL kit.

### 2.5. Immunofluorescence Microscopy

Cells were cultured and treated on glass coverslips, washed twice with phosphate‐buffered saline (PBS), and fixed with 4% paraformaldehyde for 10 min, followed by 5 min of permeabilization with 0.1% Triton X‐100. Following overnight incubation at 4°C with the primary antibody, the coverslips were stained with the secondary antibody for 30 min at 37°C. The secondary antibodies included Alexa Fluor 488 goat anti‐rabbit IgG (H + L), Alexa Fluor 594 goat anti‐mouse IgG (H + L), and Alexa Fluor 647 donkey anti‐mouse IgG (H + L). The cells were thereafter washed three times with PBS in a dark chamber, after which the coverslips were mounted with antifade reagent containing DAPI (#P36935, Life Technologies). Mitochondria were visualized using a confocal microscope (Carl Zeiss LSM800, 63 x /1.4 NA oil), with imaging performed in ZEISS Airyscan multiplex mode. A 1 μm *Z*‐stack was captured, and the images represent maximum intensity projections of 10–14 *Z*‐planes. A total of 15–20 fields of view were captured for each group for each replicate. The fluorescence intensity in the control group was first calibrated to optimize imaging conditions. The same imaging parameters were thereafter applied to the experimental group to ensure consistency and enable accurate comparison. Mitochondrial numbers were quantified using the ImageJ software. The morphological analysis was conducted on more than 100 cells in each experimental group.

### 2.6. Cell Proliferation Analysis

At the beginning of the experiment, a total of 3 × 10^4^ cells per well were seeded into 12‐well plates. At the end of the experiment (either drug treatment with fatostatin or PF429242 or B166 knockdown), the adherent cells were dissociated from the bottom of the dish using trypsin and then suspended in a fresh culture medium. The number of cells was quantified using a hemocytometer, and the proportion of dead cells was determined using trypan blue solution.

### 2.7. Colony Formation Assay

GBM cell lines U251 and U87 (2 × 10^3^) were plated into a 6 cm dish. Three 6‐cm cell culture dishes were used for each group per replicate. After 14 days of culture, colonies were formed and were visible to the naked eye. Subsequently, these colonies were stained with 0.1% crystal violet, washed, photographed with a digital camera, and counted under a light microscope. Each experiment was conducted in triplicate and repeated three times.

### 2.8. Quantitative Reverse Transcription Polymerase Chain Reaction (RT‐qPCR)

Total RNA was extracted using TRIzol reagent in accordance with the manufacturer’s instructions, and cDNA was synthesized using the iScript cDNA Synthesis kit. RT‐qPCR was conducted using the iQ SYBR Green Supermix on the Applied Biosystems 7900HT Real‐Time PCR System. The expression levels were normalized to the *36B4* housekeeping gene and calculated using the comparative 2^-ΔΔCt^ method. The amplification conditions were set as follows: 95°C for 10 min, followed by denaturation at 95°C for 30 s, annealing at 60°C for 30 s, and extension at 72°C for 30 s, with 45 cycles. A total of five replicate wells were prepared for each reaction, and each experiment was repeated at least four times. The primers utilized for RT‐qPCR are listed in Table S2.

### 2.9. ROS Detection

For live cells, CellROX Deep Red dye was used to detect mitochondrial ROS. Cells were thrice washed with PBS and incubated in a FluoroBrite DMEM containing 5% fetal bovine serum, 0.5 μM CellROX Deep Red, and MitoTracker Green for 30 min. The control group was treated with a 1/1000 dilution of DMSO. The cells were washed with PBS twice, incubated with DAPI for 20 min, and then visualized by confocal microscopy (Carl Zeiss LSM800, 63 x /1.4 NA oil). Imaging was performed in ZEISS Airyscan multiplex mode, capturing a 1 μm‐wide *Z*‐stack. The images represent maximum intensity projections of 10–14 *Z*‐planes. More than 100 cells were analyzed, and fluorescence intensity was quantified by ImageJ software.

### 2.10. Statistical Analysis

RNA‐seq data from TCGA and GTEX databases were downloaded using the UCSC Xena browser (http://xena.ucsc.edu/) [24]. These datasets were analyzed as provided, following the standard normalization and processing procedures documented by the UCSC Xena platform. Normalized data are listed in Table S3. All experiments were performed with four biological replicates, unless indicated otherwise. Categorical variables were compared using the chi‐square test or Fisher’s exact test. Quantitative variables were compared using Student’s *t*‐test or the Wilcoxon rank‐sum test. Unsupervised hierarchical clustering was employed to explore associations between variables. Survival curves were generated by Kaplan–Meier analysis, and statistically significant differences were assessed using the log‐rank test. Comprehensive patient information is presented in Tables S4–S6. The statistical analysis was performed using GraphPad Prism or R software, and the threshold for statistical significance was set at *p* = 0.05. Correlation analyses were conducted with reporting Pearson’s coefficient *r* and 95% confidence intervals (CIs). Group comparisons were reported with mean differences and 95% CIs (for continuous variables) or risk differences/odds ratios (OR) with 95% CIs (for categorical variables). Effect sizes were reported as Cohen’s *d* for continuous outcomes. Multiple comparisons were adjusted using the Bonferroni method where applicable. All figures were representative of at least three biological replicates with similar results, unless stated otherwise.

## 3. Results

### 3.1. B166 Is a Valuable Predictive and Prognostic Biomarker for Patients With GBM

RNA‐seq data from TCGA and GTEX databases were analyzed. B166 mRNA expression level significantly increased in several other cancer types, including BRCA, cholangiocarcinoma (CHLO), liver cancer (LIHC), renal chromophobia (KIRP), and endometrioid carcinoma (ESCA) compared with the corresponding adjacent normal tissues (Figure 1A). As illustrated in Figure 1B, B166 mRNA expression level was significantly upregulated in GBM tissues compared with normal cortex tissues (*P* < 0.0001). In the LGGGBM cohort, B166 expression level was higher in GBM tissues than that in lower‐grade gliomas, such as astrocytoma (mean difference = 1.6, 95% CI [1.2, 2.0], *p*  < 0.0001), oligoastrocytoma (mean difference = 1.8, 95% CI [1.4, 2.2], *p*  < 0.0001), and oligodendroglioma (mean difference = 2.1, 95% CI [1.7, 2.5], *p*  < 0.0001) (Figure 1C). Analysis of tumor tissues from GBM patients also revealed that the B166 protein level was significantly elevated compared with healthy brain tissues from patients who suffered from car accidents (fold change = 3.2, 95% CI [2.4, 4.0], *p*  < 0.001) (Figure 1D).

Figure 1B166 is a valuable predictive and prognostic biomarker for patients with GBM. (A) Boxplot analysis of B166 expression level in tumor tissues versus adjacent normal tissues in patients with BRCA (mean difference = 1.8, 95% CI [1.4, 2.2], *p*  < 0.0001, Cohen’s *d* = 0.85), CHOL (mean difference = 1.5, 95% CI [1.1, 1.9], *p*  < 0.0001, *d* = 0.73), ESCA (mean difference = 1.6, 95% CI [1.2, 2.0], *p*  < 0.05, Cohen’s *d* = 0.78), KIRP (mean difference = 1.3, 95% CI [0.9, 1.7], *p*  < 0.05, Cohen’s *d* = 0.65), LIHC (mean difference = 2.0, 95% CI [1.6, 2.4], *p*  < 0.0001, Cohen’s *d* = 0.95), and UCEC (mean difference = 1.4, 95% CI [1.0, 1.8], *p*  < 0.001, Cohen’s *d* = 0.72). (B) Analysis of B166 mRNA expression level in human GBM tumor tissues (*n* = 153) and normal cortex brain tissues (*n* = 105) obtained from TCGA and GTEX databases (mean difference = 2.4, 95% CI [2.0, 2.8], *p*  < 0.0001). (C) Analysis of B166 mRNA expression level in glioma tissues in the LGGGBM cohort from the TCGA database. As, *n* = 197; Oa, *n* = 134; Od, *n* = 198; GBM, *n* = 164. (D) Representative Western blot images of B166 from normal brain tissues of 4 car‐accident patients and GBM tumor tissues from 4 patients in WFPH. Actin was used as a loading control. (E) and (F) IHC analysis of B166, Ki‐67, EGFR, and PD‐L1 expression levels in glioma tissues in the TMA (PHG‐1 cohort, *n* = 88). Scale bar, 20 µm. Grade I, *n* = 14; grade II, *n* = 36; grade III, *n* = 26; grade IV, GBM, *n* = 12. (G) Kaplan–Meier plot of OS based on IHC *H*‐scores in the PHG‐1 cohort (*n* = 88, *p* = 0.0039). An optimal cutoff was applied to stratify high versus low groups. (H) Kaplan‒Meier plot of OS based on B166 mRNA level in the LGGGBM cohort from the TCGA database (*p* = 0.001). An optimal cutoff was applied to stratify high versus low groups. GBM, glioblastoma multiforme; BRCA, breast cancer; CHLO, cholangiocarcinoma; ESCA, endometrioid carcinoma; KIRP, renal chromophobia; LIHC, liver cancer; UCEC, uterine corpus endometrial carcinoma; As, astrocytoma; Oa, oligoastrocytoma; Od, oligodendroglioma.

**Figure:**
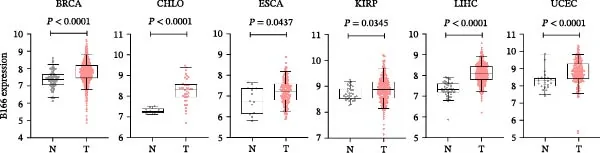
(A)

**Figure:**
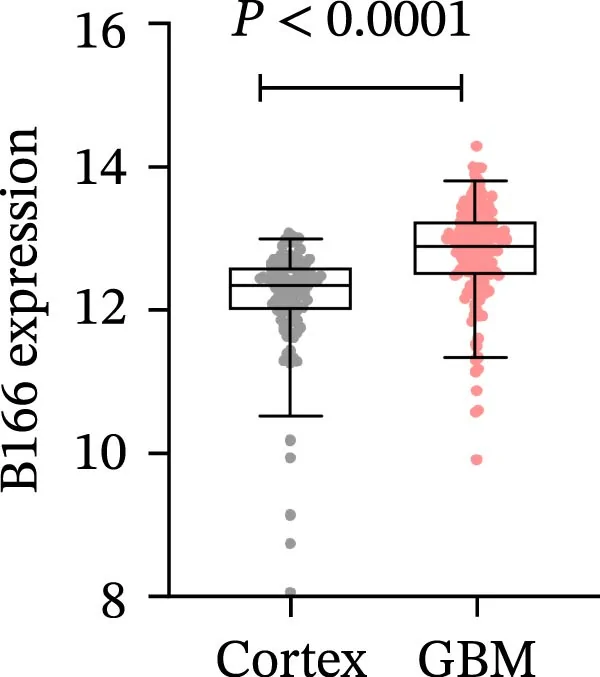
(B)

**Figure:**
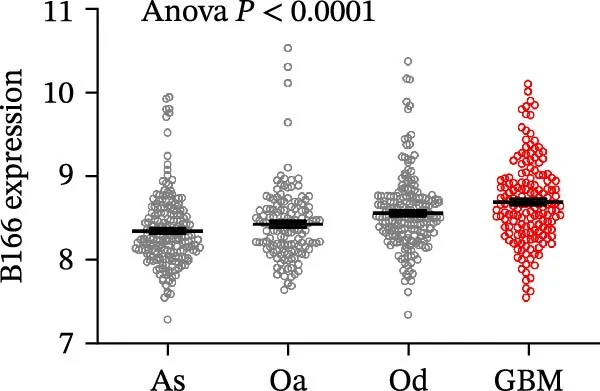
(C)

**Figure:**
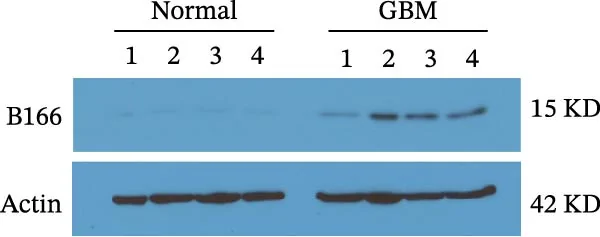
(D)

**Figure:**
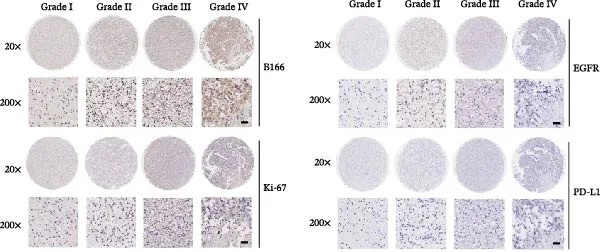
(E)

**Figure:**
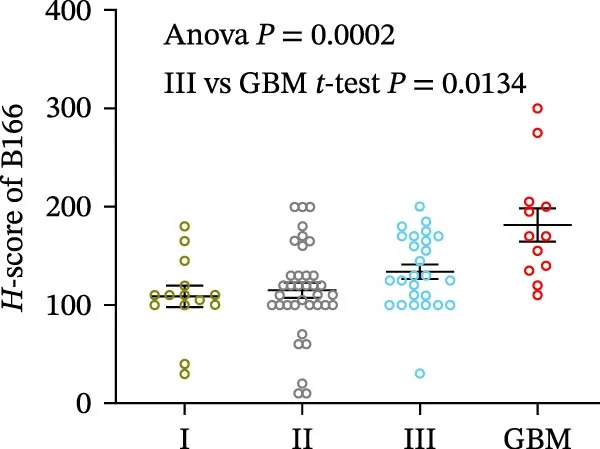
(F)

**Figure:**
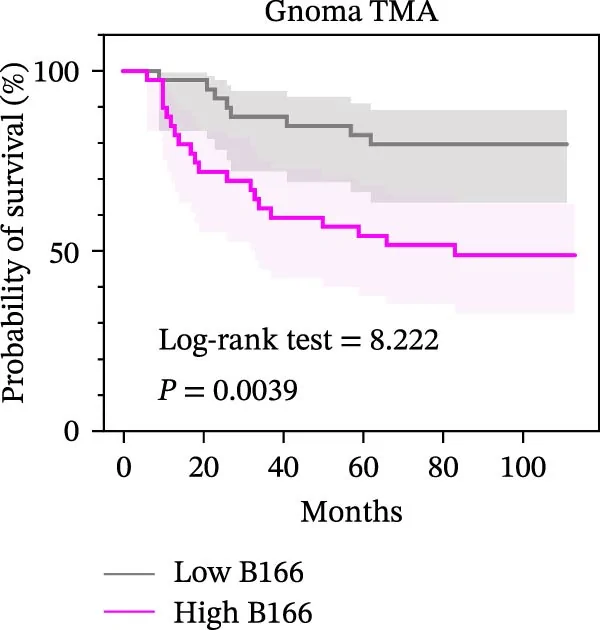
(G)

**Figure:**
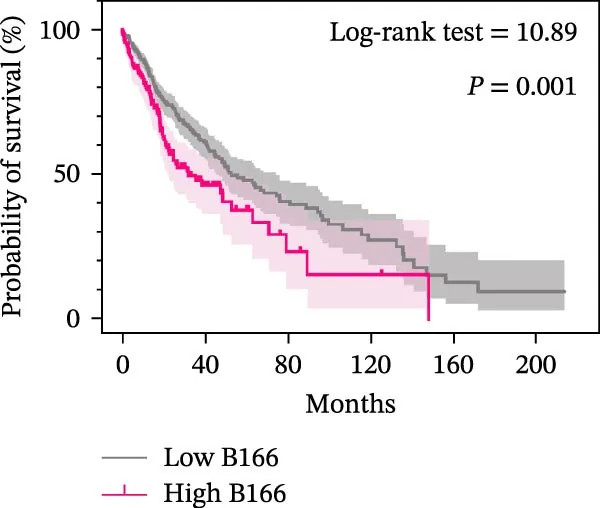
(H)

To extend the clinical investigation, the expression level of B166 in 88 biopsy tissues from the PHG‐1 cohort was further analyzed using TMA. A significantly higher level of B166 protein in GBM was found compared with WHO grade I–III gliomas. Moreover, IHC results of B166 level were more accurate and consistent with histological classification compared with classical protein tumor markers, such as Ki‐67, EGFR, and PD‐L1, indicating that B166 may serve as a potential target for GBM treatment (Figure 1E,F). The association between B166 expression level and GBM prognosis was also explored. As displayed in Figure 1G, patients in the PHG‐1 cohort with high‐B166 gliomas, stratified according to *H*‐scores, exhibited a substantial difference in OS (HR = 2.6, 95% CI [1.4, 4.8], *p* = 0.0039). The median disease‐free survival (DFS) for high‐B166 gliomas was 49 months (76 months for low‐B166 gliomas) (Table 1). The analysis of the LGGGBM cohort further confirmed an inverse correlation between high‐B166 expression level and glioma patients’ OS (HR = 1.8, 95% CI [1.3, 2.5], *p* = 0.001) (Figure 1H).

**Table 1:** Overview of the 88 patients with different grades of glioma from the PHG‐1 cohort.

Clinical and pathological parameters	N	B166 high level *H*‐score > 120 (*n* = 41)	B166 low level *H*‐score ≤ 120 (*n* = 47)	*p*‐Value
Age at diagnosis (years)				
Median	41	45	37	—
> 55	23	15	8	0.0657
≤ 55	65	26	39	—
Gender				
Male	55	27	28	0.6993
Female	33	14	19	—
Tumor location				0.2806
Frontal	22	12	10	—
Temporal	28	13	15	—
Parietal	5	0	5	—
Occipital	5	3	2	—
Multifocal	9	6	3	—
Ventricle	5	2	3	—
Others	14	5	9	—
WHO grade				0.01017 ^∗^
I	14	3	11	—
II	36	13	23	—
III	26	16	10	—
IV	12	9	3	—
EGFR IHC status				0.8158
Positive	63	30	33	—
Negative	25	11	14	—
Ki‐67 IHC status				0.003499 ^∗^
Positive	58	34	24	—
Negative	30	7	23	—
PD‐L1 IHC status				6.089ee–05 ^∗^
Positive	24	27	10	—
Negative	64	14	37	—
Recurrent status				0.03013 ^∗^
Nonrecurrent	42	14	28	—
Recurrent	46	27	19	—
Survival (months)				
Median OS	81.5	75	84	—
Median DFS	71	49	76	—
Follow‐up range	9–120	—	—	—

*Note:* The significance of B166 expression level in clinicopathologic parameters was analyzed by Pearson’s chi‐squared test. If the expected counts were less than 5, Fisher’s exact test was used ( ^∗^ *P* < 0.05).

Abbreviations: DFS, disease‐free survival; EGFR, epidermal growth factor receptor; IHC, immunohistochemistry; OS, overall survival.

### 3.2. High B166 Expression Level Was Highly Correlated With SREBP1 Expression Level in GBM Patients

As shown in Figure 2A, RNA expression levels of SREBP1 and B166 were analyzed in 152 primary GBM tissues from the TCGA GBM cohort (Pearson’s *r* = 0.44, 95% CI [0.31, 0.55], *p*  < 0.0001). G6PD, SCD, and FASN, the known targets of SREBP1, showed Pearson’s coefficients of 0.62 (95% CI [0.52, 0.70]), 0.63 (95% CI [0.53, 0.71]), and 0.39 (95% CI [0.25, 0.51]), respectively (all *p*  < 0.0001). GBM tissues were then divided into two groups based on SREBP1 expression level. Patients with high SREBP1 glioblastomas exhibited increased expression levels of B166 (55/82, 67% vs 34/70, 48%; risk difference RD = 0.19, 95% CI [0.02, 0.36], *p* = 0.032; OR = 2.19, 95% CI [1.07, 4.48]), FASN (54/82, 66% vs 31/70, 44%; RD = 0.22, 95% CI [0.05, 0.39], *p* = 0.012; OR = 2.48, 95% CI [1.21, 5.08]), and SCD (61/82, 74% vs 21/70, 30%; RD = 0.44, 95% CI [0.29, 0.59], *p*  < 0.0001; OR = 6.73, 95% CI [3.32, 13.66]) compared with patients with low SREBP1 glioblastomas (Figure 2B). Patients with high SREBP1 expression levels also showed statistically higher expression levels of B166 (mean difference = 0.8, 95% CI [0.4, 1.2], Cohen’s *d* = 0.65, *p*  < 0.01), FASN (mean difference = 1.0, 95% CI [0.6, 1.4], *d* = 0.78, *p*  < 0.01), and SCD (mean difference = 1.4, 95% CI [1.0, 1.8], *d* = 1.05, *p*  < 0.01) than those with low SREBP1 expression levels (Figure 2C). Meanwhile, a transcriptional signature was derived from an unsupervised heatmap, confirming correlations among B166, SREBP1, G6PD, SCD, and FASN. The clinical relevance of B166 and SREBP1 was further supported by unsupervised clustering analysis, compared with FASN and SCD (Figure 2D). The dendrogram indicated that B166 was likely regulated by SREBP1, similar to FASN and SCD (Figure 2E). The correlation between B166 and SREBP1 was also evaluated in 12 glioblastoma cell lines using RNA‐seq data from the American Type Culture Collection. Pearson’s correlation analysis confirmed a significant correlation (*r* = 0.47, 95% CI [−0.05, 0.80], *p* = 0.002, Figure 2F). In 12 GBM specimens from the PHG‐1 cohort, a significant correlation between B166 and SREBP1 expression levels was identified (Figure 2G,H). The association of B166 with other known or potential glioma biomarkers, such as Ki‐67, EGFR, and PD‐L1, was examined (Figure 2G). Pearson’s correlation coefficient of the IHC *H*‐score was 0.67 (95% CI [0.15, 0.90], *p* = 0.012) (Figure 2I). Additionally, SREBP1 expression level exhibited a moderate correlation with G6PD expression level, an essential antioxidant enzyme and a known SREBP1 target, with a Pearson’s correlation coefficient of 0.82 (95% CI [0.48, 0.95], *p* = 0.0006) (Figure 2I).

Figure 2The high expression of B166 is highly correlated with SREBP1 in GBM patients. (A) The correlation between B166, G6PD, SCD, FASN, and SREBP1. (B) The mRNA levels of B166, FASN, and SCD were associated with SREBF1 mRNA levels, as analyzed by Pearson’s chi‐squared test. (C) Boxplot analysis of gene expression level shows that the mRNA levels of B166, FASN, and SCD were higher in the high SREBF1 group (red) than in the low SREBF1 group (blue), as assessed by the Wilcoxon rank‐sum test. (D) The clinical relevance of B166 and SREBP1 was further supported by unsupervised clustering analysis compared with FASN and SCD. (E) Two lipogenesis genes, *FASN* and *SCD1*, were significantly positively correlated with B166 in GBM patients from the TCGA database. They found possible downstream targets of SREBP1, as shown by hierarchical clustering. (F) The correlation between B166 and SREBP1 in 12 glioblastoma cell lines. (G) and (H) Representative IHC images of anti‐B166 and SREBP1 staining in 12 GBM tumor tissues in TMA. The levels of B166 and SREBP1 staining were quantified by neuropathologists. Scale bar, 200 µm in G; 20 µm in H. (I) The correlation between B166, G6PD, and SREBP1. GBM, glioblastoma multiforme; G6PD, glucose‐6‐phosphate dehydrogenase; SREBP1, sterol regulatory element‐binding protein 1; SCD1, stearoyl‐CoA desaturase 1; FASN, fatty acid synthase.

**Figure:**
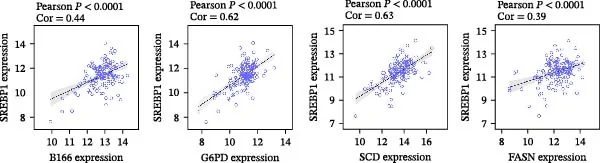
(A)

**Figure:**
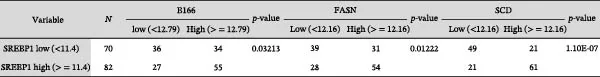
(B)

**Figure:**
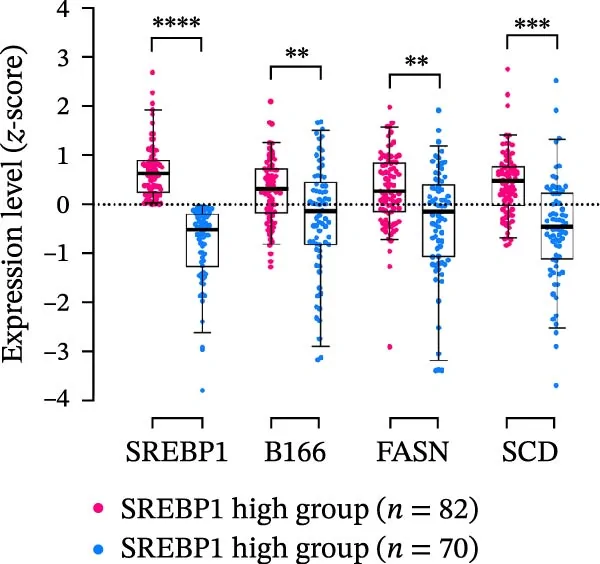
(C)

**Figure:**
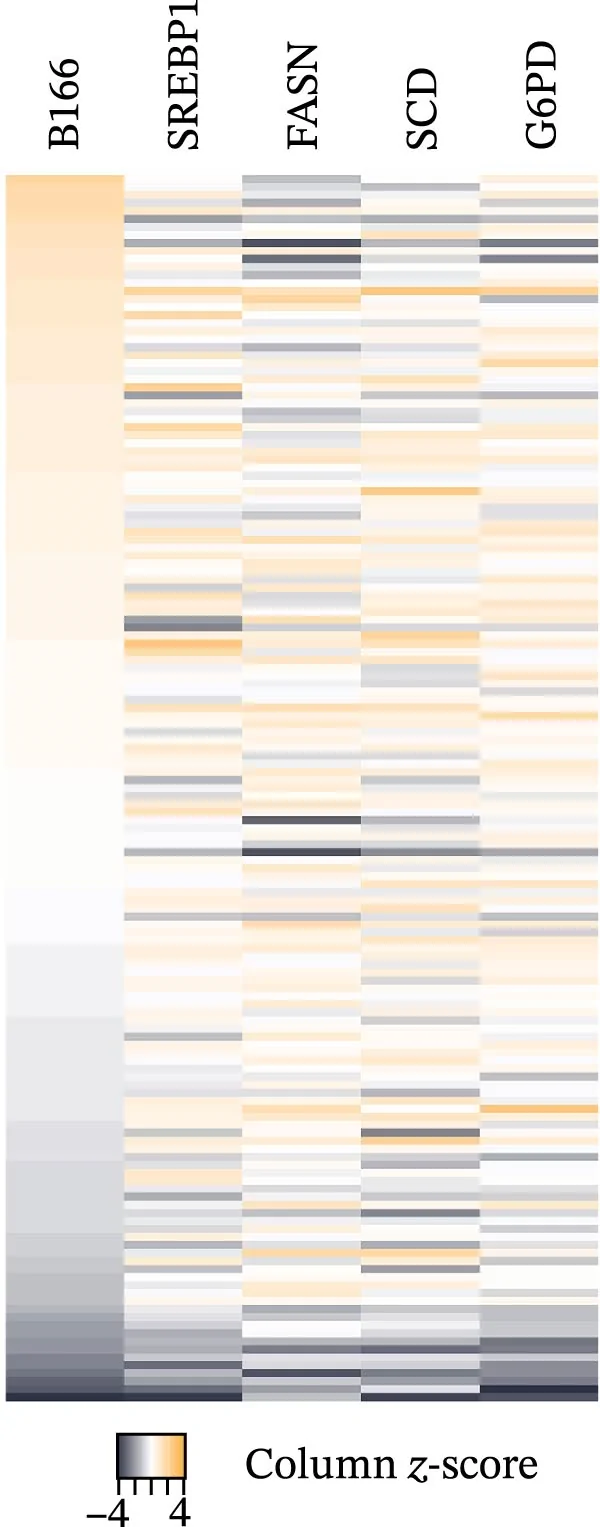
(D)

**Figure:**
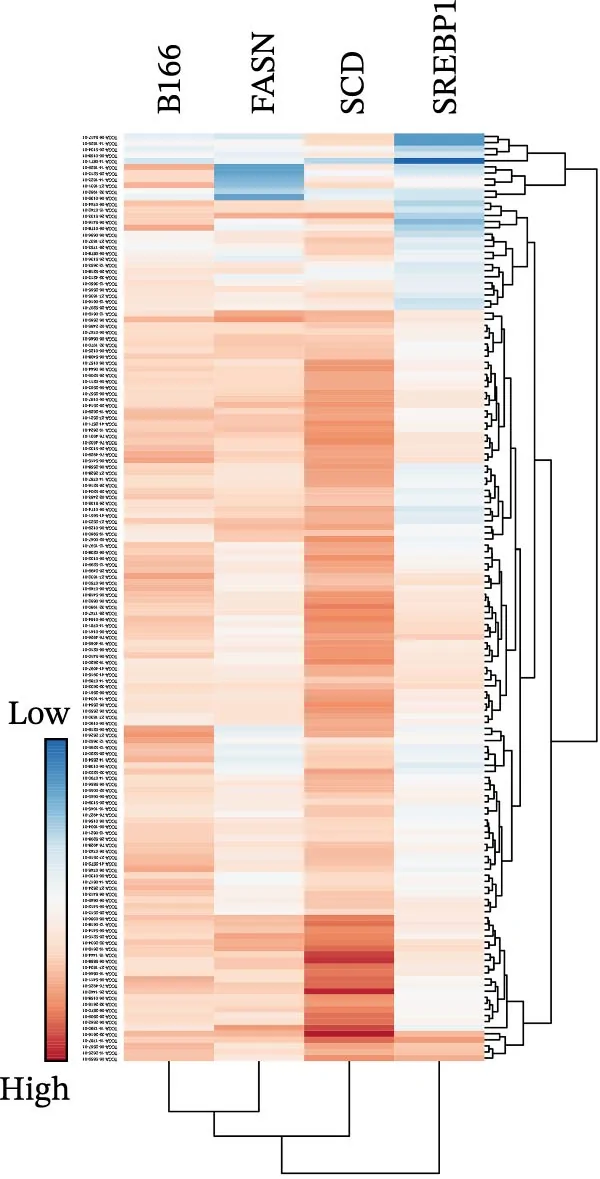
(E)

**Figure:**
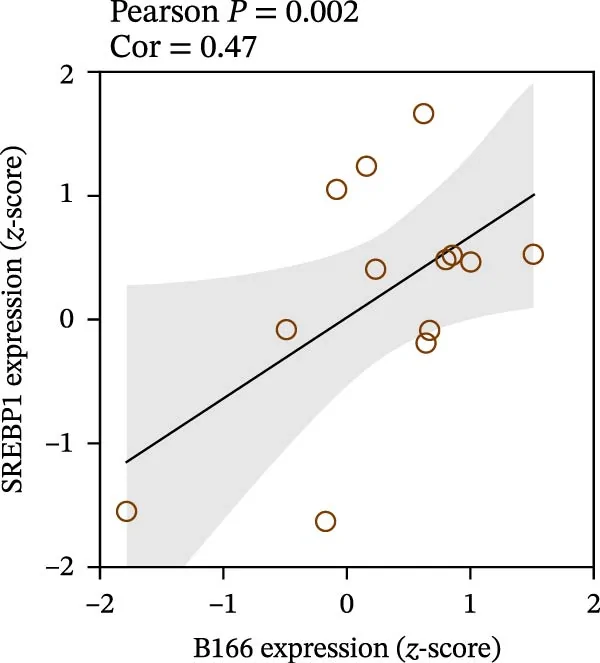
(F)

**Figure:**
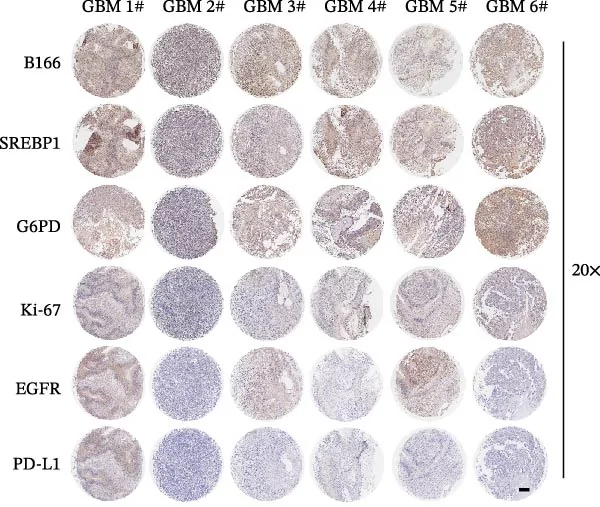
(G)

**Figure:**
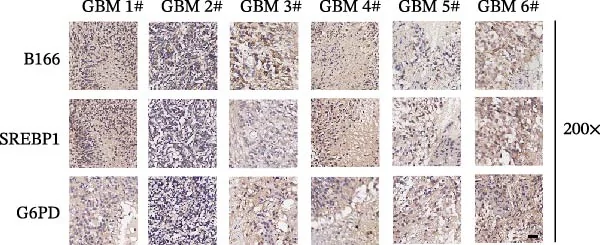
(H)

**Figure:**
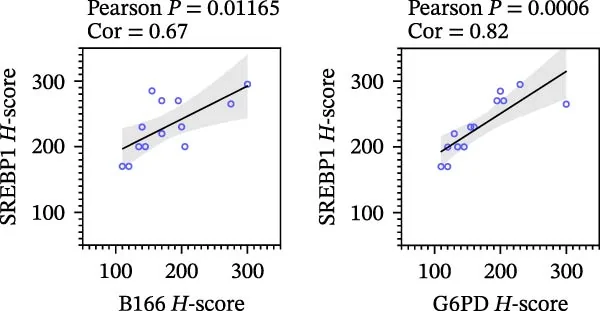
(I)

### 3.3. B166 Was Primarily Regulated by the Active N‐Terminal Form of SREBP1‐a

Adenoviruses expressing the *N*‐terminal active forms of SREBP isoforms (Ad‐SREBP1‐aN, Ad‐SREBP1‐cN, and Ad‐SREBP2‐N) were used to determine which isoform could regulate B166 expression level. RT‐qPCR revealed a significant increase in B166 and FASN RNA levels upon expression of active SREBPs, suggesting transcriptional regulation by SREBPs. Ad‐SREBP1‐aN was noted as the primary regulator of B166 and FASN, while active *N*‐terminal SREBP1‐c or SREBP2 exerted only minor transcriptional effects on B166 and FASN (Figure 3A). The B166 protein level was elevated upon SREBP1‐a expression, corresponding to RNA levels, whereas SREBP1‐c and SREBP2 weakly regulated B166 protein level (Figure 3B). These data supported that the active *N*‐terminal form of SREBP1‐a was the most potent isoform for upregulating B166 expression level. Stable knockdown GBM cell lines were generated using lentivirus‐mediated short hairpin RNAs (shRNAs). RT‐qPCR revealed a significant reduction in the RNA levels of B166, FASN, and SCD1, along with a notable decrease in SREBP1‐a and SREBP1‐c RNA levels in SREBP1 knockdown cells (Figure 3C). Western blotting confirmed modest downregulation of B166 and FASN protein levels after SREBP1 depletion, as well as strong reduction of SCD1 protein level upon SREBP1 knockdown (Figure 3D). These findings are in line with the correlation analysis results, demonstrating a modest correlation between B166 and SREBP1 in GBM cells.

Figure 3B166 is primarily regulated by the active N‐terminal form of SREBP1‐a. (A) RT‐qPCR of FASN, B166, and SCD1 in U251 and U87 cells after adenoviral overexpression of the *N*‐terminal form of SREBP1‐a, SREBP1‐c, or SREBP2 for 48 h (mean ± SD, *n* = 3). SREBP1‐aN vs. Control: B166: Fold change = 3.2, 95% CI [2.6, 3.8], *p*  < 0.01, Cohen’s *d* = 1.85; FASN: Fold change = 4.1, 95% CI [3.3, 4.9], *p*  < 0.01, Cohen’s *d* = 2.15; SCD1: Fold change = 3.8, 95% CI [3.1, 4.5], *p*  < 0.01, Cohen’s *d* = 2.02. SREBP1‐cN vs. Control: B166: Fold change = 1.5, 95% CI [1.2, 1.8], *p*  < 0.01, Cohen’s *d* = 0.75; FASN: Fold change = 1.8, 95% CI [1.4, 2.2], *p*  < 0.01, Cohen’s *d* = 0.95. SREBP2‐N vs. Control: B166: Fold change = 1.3, 95% CI [1.0, 1.6], *p*  < 0.01, Cohen’s *d* = 0.45. Five replicates were used per reaction, and each experiment was repeated at least four times. (B) Representative Western blots (*n* = 3 blots) of SREBP1‐a or SREBP1‐c (*N*,*N*‐terminal cleavage form), SREBP2 (*N*,*N*‐terminal cleavage form), B166, SCD1, and FASN after adenovirus infection with the overexpressed *N*‐terminal cleavage form of SREBP1‐a or ‐c or SREBP2 for 48 h. (C) RT‐qPCR of SREBF1‐a, SREBF1‐c, FASN, B166, and SCD1 mRNA expression levels (mean ± SD, *n* = 3) in GBM U251 and U87 cells after shRNA‐mediated SREBP1 knockdown for 48 h. shSREBP1 vs. Control: SREBP1‐a RNA: Fold change = 0.25, 95% CI [0.18, 0.32], *p*  < 0.01, Cohen’s *d* = 2.35. SREBP1‐c RNA: Fold change = 0.30, 95% CI [0.22, 0.38], *p*  < 0.01, Cohen’s *d* = 2.10. B166 RNA: Fold change = 0.45, 95% CI [0.35, 0.55], *p*  < 0.01, Cohen’s *d* = 1.45. FASN RNA: Fold change = 0.40, 95% CI [0.31, 0.49], *p*  < 0.01, Cohen’s *d* = 1.65. SCD1 RNA: Fold change = 0.35, 95% CI [0.26, 0.44], *p*  < 0.01, Cohen’s *d* = 1.85. Five replicates were used per reaction, and each experiment was repeated at least four times. (D) Representative Western blots (*n* = 3 blots) of SREBP1 (P, SREBP1 precursor, the full length of SREBP1 protein; *N*,*N*‐terminal cleavage form), B166, SCD1, and FASN expression levels after shRNA‐mediated SREBP1 knockdown for 48 h. (E) U251 and U87 cells were treated with the SREBP1 inhibitors fatostatin (upper panel) or PF429242 (lower panel), respectively, at a range of doses as indicated for 2 days. 10 µM Fatostatin: U251 cell death rate = 77% (95% CI [70%, 84%], *p*  < 0.001), vs. Control: Risk difference = 0.75, OR = 12.5 (95% CI [8.2, 19.1]). U87 cell death rate = 83.6% (95% CI [77%, 90%], *p*  < 0.001), vs. Control: Risk difference = 0.81, OR = 24.3 (95% CI [14.5, 40.8]). 2 µM PF429242: U251 cell death rate = 81.2% (95% CI [75%, 87%], *p*  < 0.001), vs. Control: Risk difference = 0.79, OR = 18.7 (95% CI [11.2, 31.2]). U87 cell death rate = 88.8% (95% CI [83%, 94%], *p*  < 0.001), vs. Control: Risk difference = 0.86, OR = 45.2 (95% CI [24.8, 82.3]). Representative images show cell morphology after drug treatment; then, cells were counted using the trypan blue assay. Scale bar, 20 μm. (F) Colony‐forming ability of U251 and U87 cells that silenced SREBP1 (*n* = 3). Colony formation of GBM cells treated with increasing doses of fatostatin. 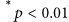. (G) RT‐qPCR analysis of FASN and B166 mRNA expression levels (mean ± SD, *n* = 3) in U87 cells after drug treatment for 48 h. Treatment with 2 µM PF429242 resulted in a relative B166 RNA level of 30% (95% CI [25%, 35%],*p*  < 0.001) and a relative FASN RNA level of 20% (95% CI [15%, 25%], *p*  < 0.001). Treatment with 10 µM Fatostatin resulted in a relative B166 RNA level of 10% (95% CI [8%, 12%], *p*  < 0.001). Five replicates were used per reaction, and each experiment was repeated at least four times. (H) Representative Western blots (*n* = 3 blots) of SREBP1 (P, SREBP1 precursor, the full length of SREBP1 protein; *N*,*N*‐terminal cleavage form), B166, SCD1, and FASN expression levels after drug treatment for 48 h. G6PD, glucose‐6‐phosphate dehydrogenase; SREBP1, sterol regulatory element‐binding protein 1; SCD1, stearoyl‐CoA desaturase 1; FASN, fatty acid synthase.

**Figure:**
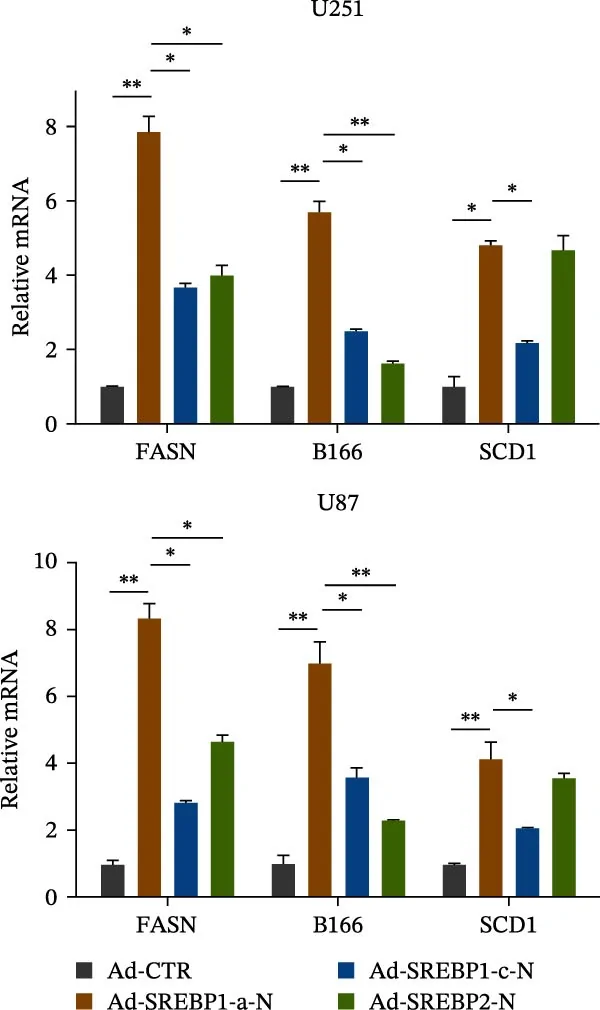
(A)

**Figure:**
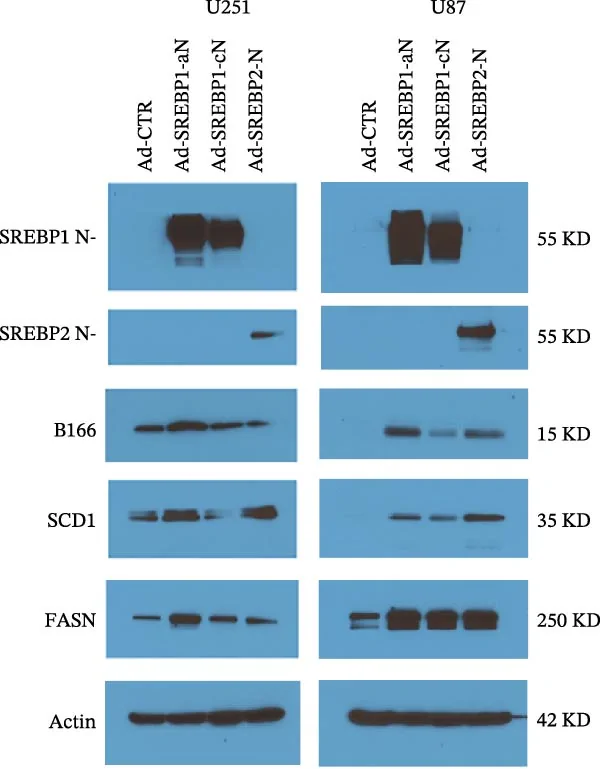
(B)

**Figure:**
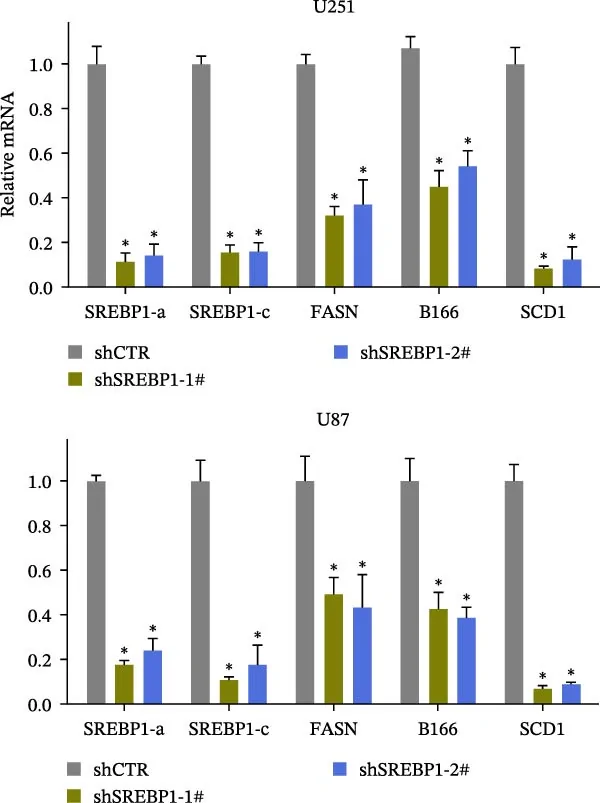
(C)

**Figure:**
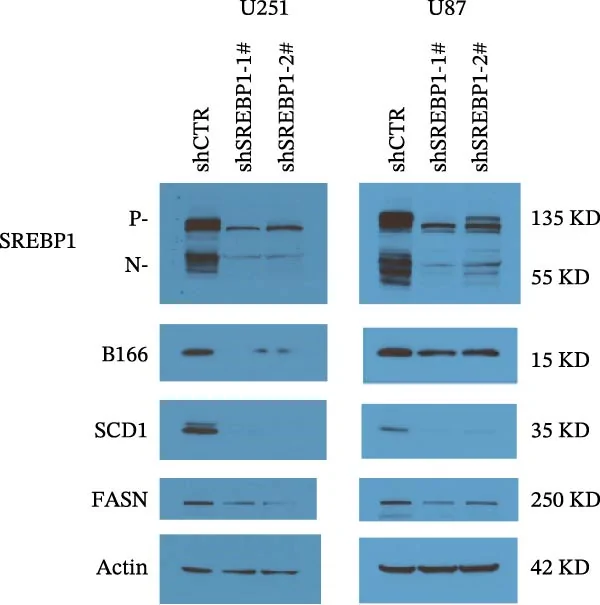
(D)

**Figure:**
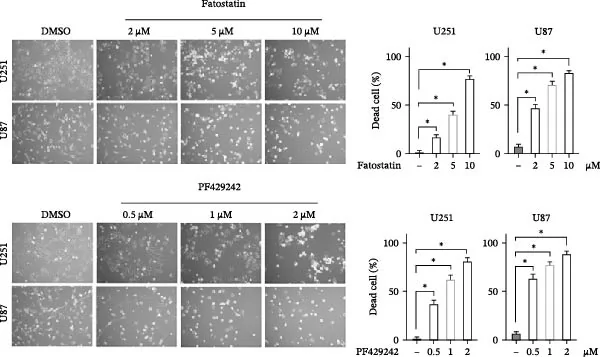
(E)

**Figure:**
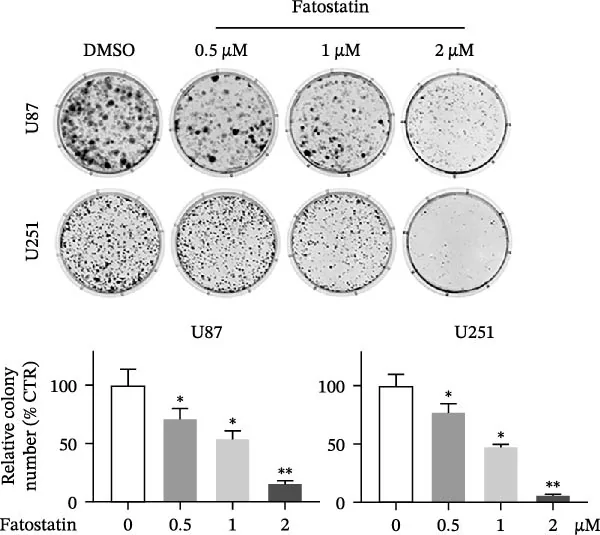
(F)

**Figure:**
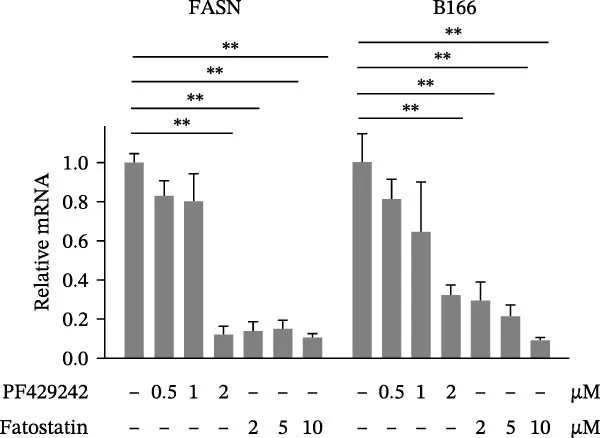
(G)

**Figure:**
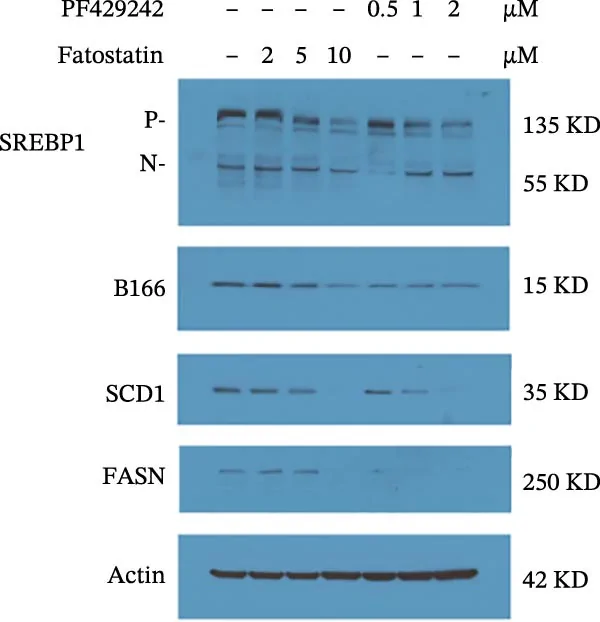
(H)

GBM cells were then treated with fatostatin and PF429242, recognized as two potent SREBP1 inhibitors that have been shown to suppress SREBP1 activity in tumor cells [25, 26]. This study indicated that both fatostatin and PF429242 effectively induced cell death after 48 hr of treatment, with most cells dying at 10 µM fatostatin (77% U251 cells, 83.6% U87 cells) and 2 µM PF429242 (81.2% U251 cells, 88.8% U87 cells) (Figure 3E). To validate the role of SREBP1 in GBM progression, SREBP1 was inhibited using fatostatin, and a colony formation assay was conducted. Expectedly, inhibiting SREBP1 significantly reduced GBM cell proliferation (Figure 3F), with dose‐dependent reductions in colony formation in both U87 and U251 cells. Colony number and average colony size were remarkably reduced in 2 µM fatostatin‐treated cells compared to control cells, in which only 20% of colonies were alive in U87 cells and 10% were alive in U251 cells. RT‐qPCR analysis showed a dose‐dependent decrease in B166 expression level. Specifically, B166 RNA level decreased to ~30% of the control cells when treated with 2 µM PF429242 and reduced to only 10% when treated with 10 µM fatostatin. FASN expression level was significantly blocked by administering 2 µM of fatostatin or 2 µM of PF429242, with the RNA levels decreasing to 20% of the controls (Figure 3G). Western blotting revealed that treatment with fatostatin and PF429242 decreased active N‐terminal SREBP1 level, resulting in reduced levels of B166, FASN, and SCD1 compared with untreated controls. The B166 protein level was gradually downregulated with increasing drug concentration, consistent with RNA expression level (Figure 3H).

### 3.4. B166‐V1 and B166‐V5 Expression Levels Were Upregulated by SREBP1‐aN, Confirming Nuclear Localization of B166‐V5 Protein

IHC was performed using the B166 antibody to investigate the cellular distribution of B166 in GBM cells. As illustrated in Figure 4A, B166 was mainly localized in the nucleus and mitochondria in both U251 and U87 cells. Comparison of the genomic sequence of the *B166* gene with the cDNA sequences of its transcripts revealed that the *B166* gene consists of six exons and has five transcript variants. B166‐V1 is the full‐length transcript, consisting of all six exons. In the endoplasmic reticulum, B166‐V1 is translated into protein isoform 1, which has a mitochondrial targeting sequence (MTS) at the N‐terminus that is cleaved upon entry into the mitochondria (Figure 4B). Compared with B166‐V1, B166‐V2 lacks the third exon, B166‐V3 lacks the second and third exons, and B166‐V4 lacks the second exon. B166‐V5 uses an in‐frame start codon of V1, resulting in a shorter first exon, and its remaining sequence is identical to that of V1, except for the MTS sequence (Figure 4B). RT‐qPCR analysis was carried out to explore which transcripts were regulated by SREBP1‐a. GBM cells were treated with PF429242, an inhibitor of SREBP1, at varying concentrations for 24 hr, or transduced with an adenovirus containing the active N‐terminal form of SREBP1‐a. Treatment with 0.2 µM of PF429242 had no effect on the B166 transcript level in GBM cells. However, treatment with 0.5 µM of PF429242 significantly suppressed B166‐V1 and B166‐V5 RNA levels and slightly suppressed B166‐V1 RNA level after 24 hr. The transcript levels of B166‐V2, ‐V3, and ‐V4 slightly varied after treatment with 0.5 µM of PF429242 (Figure 4C). Overexpression of SREBP1‐aN for 24 hr led to significantly higher transcript expression levels of B166‐V1 and B166‐V5, with a slight increase in B166‐V1 transcript expression level compared with control cells. The transcript levels of B166‐V2, ‐V3, and ‐V4 were not altered after SREBP1‐aN overexpression (Figure 4C).

Figure 4B166‐V1 and B166‐V5 are upregulated by SREBP1‐aN, confirming nuclear localization of B166‐V5 protein. (A) Representative confocal microscopy images of GBM U251 (upper panels) and U87 cells (lower panels) stained with B166 antibody (green) and DAPI (blue). Scale bar, 10 µm. (B) Schematic illustration of B166 transcript variants. Colored boxes represent six exons, and black lines represent introns. Blue lines on the left represent 5′UTR sequences, while brown lines on the right represent 3′UTR sequences. Primer 1: forward, GGGCTATATACTCGTCGGT; reverse, TGTGAGATGATATTGGGTGC. Primer 2: forward, GCAGTGGAGGTGTTTGAAG; reverse, GACGTCGATTCCCAAAGATG. Primer 3: forward, GGCTATATACTCGTCGGTGG; reverse, TCATTAACACTCAGACAGGC. (C) B166 variant amplifications using Primers 1, 2, and 3 via RT‐qPCR after PF429242 treatment for 24 h. Five transcript variants were amplified using drug‐treated or untreated U87 (upper panel) or U251 (lower panel) cells. After cycling, the products of RT‐qPCR were subjected to 2% agarose gel electrophoresis. Bands represent different transcript variants. B166 transcript variant amplification using Primers 1, 2, and 3 via RT‒qPCR after adenovirus‐mediated SREBP1‐aN overexpression for 24 h. The 5 transcript variants were amplified after adenovirus infection in U251 (upper panel) and U87 (lower panel) cells. After cycling, the products of RT‐qPCR were subjected to 2% agarose gel electrophoresis. Bands represent different transcript variants. (D) Representative confocal microscopy images of the same U251 cells infected by adenovirus, as shown in the left panel, and stained with B166 antibody (green) and DAPI (blue) (left panel). Scale bar, 10 µm. Representative Western blots (*n* = 3 blots) of U251 cells infected by adenovirus. SREBP1‐aN was overexpressed, and the B166 expression level was upregulated (right panel). (E) Left panel: Representative immunofluorescence images of anti‐flag staining (red) in U251 and U87 cells after transient expression of B166‐V5‐flag. Mitochondria were stained with anti‐COX IV (green). Nuclei were stained with DAPI (blue). B166‐V5‐flag was stained with anti‐flag staining (red), and B166‐V5 was mainly localized in the nucleus. Scale bar, 10 µm. (F) Representative Western blots (*n* = 3) of U251 cells with transient expression of B166‐V5‐flag. (G) Models of transcript variants, protein isoforms, and cellular localizations of B166. Two transcripts of B166, transcript variants 1 and 5, are transcribed from the *B166* gene. Protein isoform 1, translated from transcript variant 1, contains a mitochondrial targeting sequence (MTS) that directs it to the mitochondria, where it is subsequently cleaved to form the functional B166. In contrast, protein isoform 5, translated from transcript variant 5, lacks the MTS, utilizes the in‐frame start codon of the full‐length transcript, and localizes to the nucleus to form the functional B166. SREBP1, sterol regulatory element‐binding protein 1.

**Figure:**
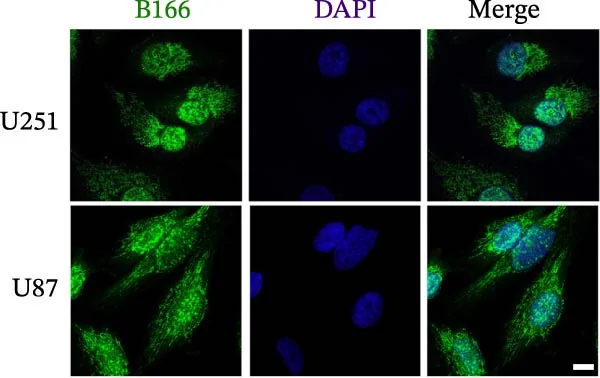
(A)

**Figure:**
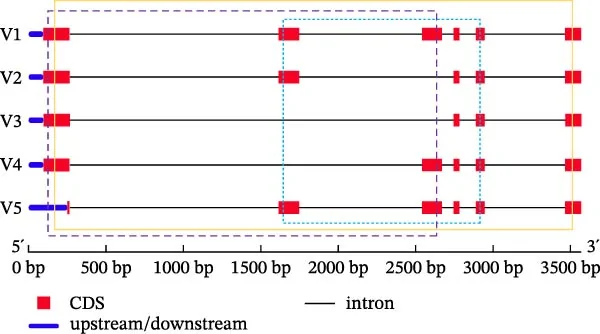
(B)

**Figure:**
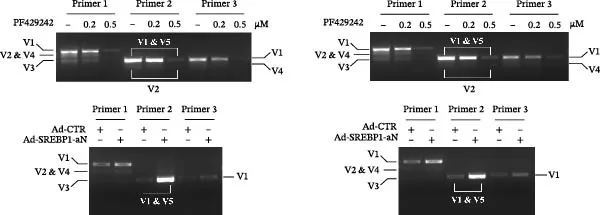
(C)

**Figure:**
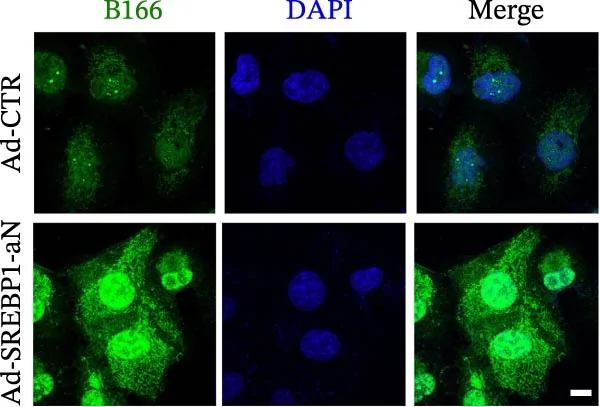
(D)

**Figure:**
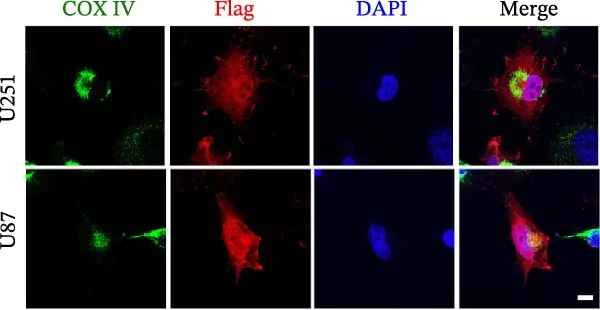
(E)

**Figure:**
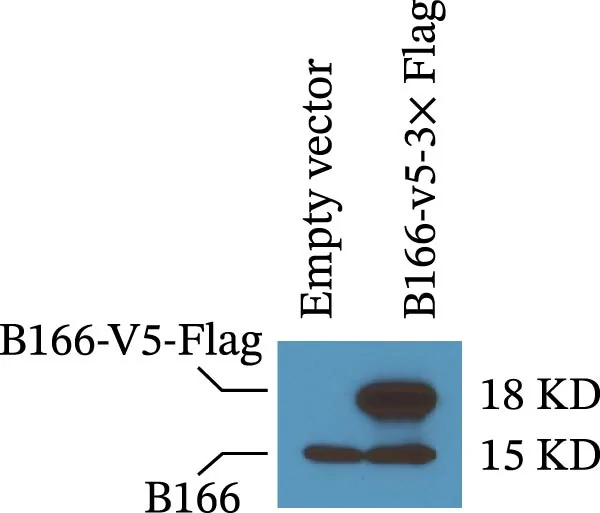
(F)

**Figure:**
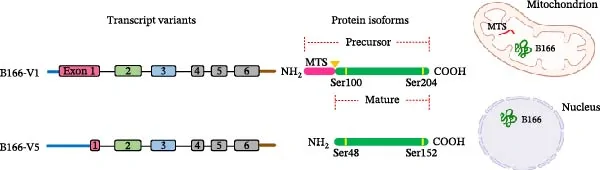
(G)

Immunostaining analysis was conducted to examine the transcript regulation of B166 by SREBP1‐aN in GBM cells. Fluorescence imaging revealed that SREBP1‐aN overexpression significantly elevated B166 expression level in both mitochondria and nuclei compared with control cells infected with empty adenoviruses (Figure 4D). The fluorescence signal was markedly accumulated in the nucleus upon the overexpression of B166‐V5‐Flag (tag), indicating nuclear localization of B166‐V5 in GBM cells. This was further validated by co‐localization of the DAPI signal (nucleus marker, blue) with B166‐V5‐Flag. In contrast, the mitochondrial marker COX IV exhibited no co‐localization with B166‐V5‐Flag after 48 hr of transient expression (Figure 4E,F). These findings collectively support that SREBP1‐aN regulates B166‐V1 and B166‐V5 expression levels and confirm the presence of B166‐V5 protein in the nucleus (Figure 4G).

### 3.5. B166 Suppression Induced Cell Death in GBM Through Mitochondrial Damage and Increased ROS

Two siRNAs were designed and synthesized to suppress B166‐V1 or B166‐V5 separately. si3′UTR targets the 3′UTR sequence shared by all transcripts (V1–V5), while si5′UTR targets the shared sequence upstream of the first four transcripts of B166 (V1–V4). Depletion of B166 by si5′UTR or si3′UTR induced significant, time‐dependent cell death in GBM cells, and almost no viable cells were detected after 3 days of transfection (Figure 5A,B). Confocal imaging revealed that mitochondrial B166‐V1 protein was significantly reduced after 2 days of knockdown by si5′UTR, compared with control cells treated with siCTR (scramble siRNA). Inhibition of B166 by si3′UTR resulted in a remarkable reduction in B166‐V1 protein level, and the fluorescence signal in mitochondria was almost completely diminished after 48 hr. B166‐V5 protein level was also significantly reduced by si3′UTR, with a significantly weaker nuclear fluorescence signal compared with control cells or si5′UTR knockdown cells (Figure 5C).

Figure 5B166 suppression induces cell death in GBM via mitochondrial damage and increased ROS. (A) Representative Western blots (*n* = 3 blots) of U87 cells treated with 20 nM 5′UTR or 3′UTR siRNAs for 48 h. (B) The percentage of dead cells was counted after 5′UTR or 3′UTR siRNA knockdown for 3 days (mean ± SD, *n* = 3) (left panel: U87 cells; right panel: U251 cells). si5′UTR: U87 cell death rate = 85% (95% CI [78%, 92%]), RD = 0.83, OR = 28.3. U251 cell death rate = 88% (95% CI [82%, 94%]), RD = 0.86, OR = 38.5. si3′UTR: U87 cell death rate = 92% (95% CI [87%, 97%]), RD = 0.90, OR = 57.6. U251 cell death rate = 95% (95% CI [90%, 100%]), RD = 0.93, OR = 76.8.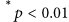. (C) Left panel: Representative fluorescence images (*n* = 15 images) of B166 staining in U87 cells treated with 20 nM 5′UTR or 3′UTR siRNAs for 48 h. B166: green; DAPI: blue. Scale bar, 10 µm. Middle panel: More than 100 cells were quantified to determine the fluorescence intensity of B166‐V1 in the mitochondria after U87 cells were treated with 20 nM 5′UTR or 3′UTR siRNAs for 48 h (mean ± SEM). 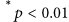. Right panel: More than 100 cells were quantified to determine the fluorescence intensity of B166‐V5 in the nucleus after U87 cells were treated with 20 nM 5′UTR or 3′UTR siRNAs for 48 h (mean ± SEM). For si3′UTR treatment, mitochondrial B166‐V1 fluorescence intensity decreased by 85% (95% CI [78%, 92%], Cohen’s *d* = 2.45), while nuclear B166‐V5 fluorescence intensity decreased by 75% (95% CI [68%, 82%], Cohen’s *d* = 2.15).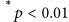. (D)–(G) Representative fluorescence imaging (*n* = 20 images) of ROS (green) detected with CellROX and mitochondria (red) detected with MitoTracker in U251 cells treated with/without PF429242 or 3′UTR siRNA. Scale bar, 10 µm. ROS levels and mitochondrial morphologies were quantified in more than 100 cells (mean ± SEM). 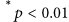. GBM, glioblastoma multiforme.

**Figure:**
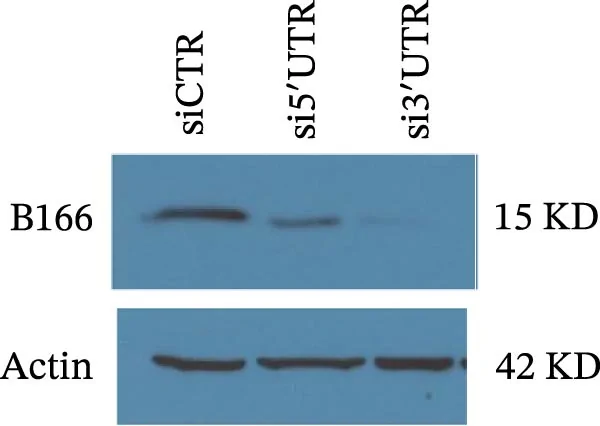
(A)

**Figure:**
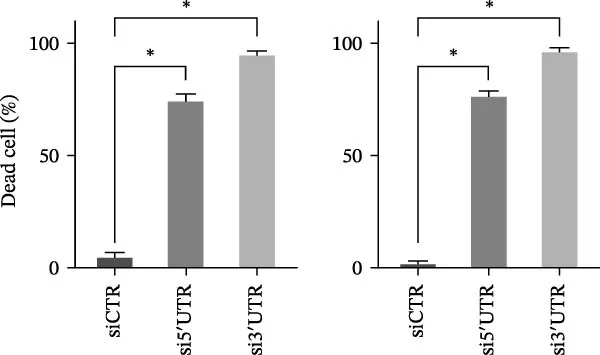
(B)

**Figure:**
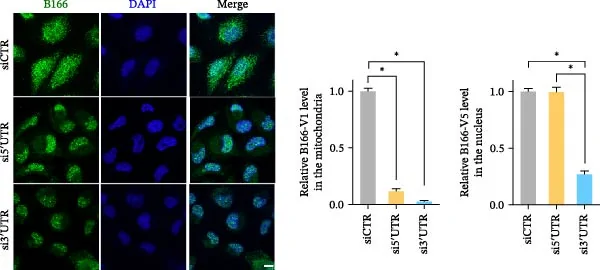
(C)

**Figure:**
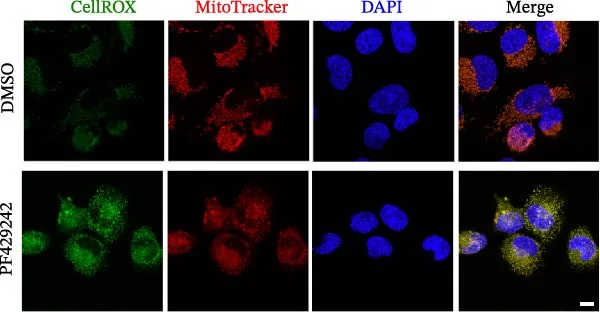
(D)

**Figure:**
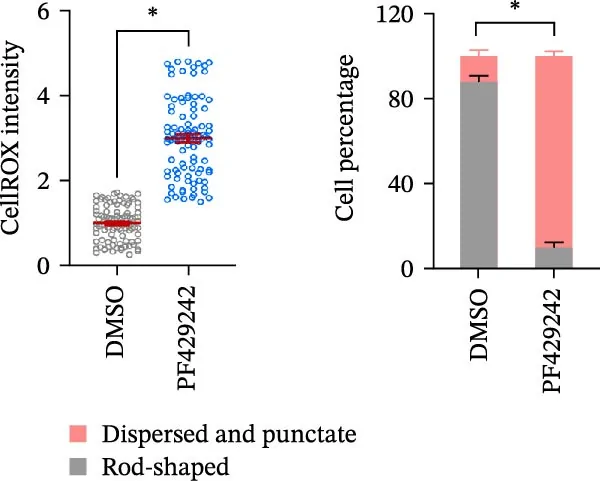
(E)

**Figure:**
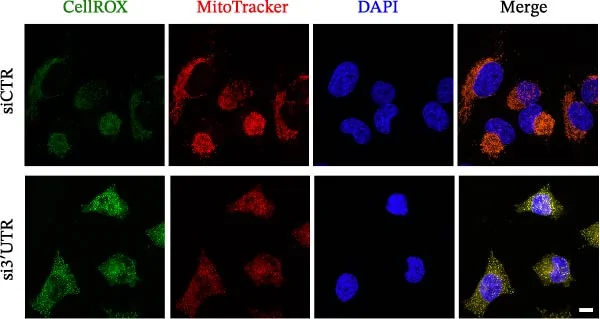
(F)

**Figure:**
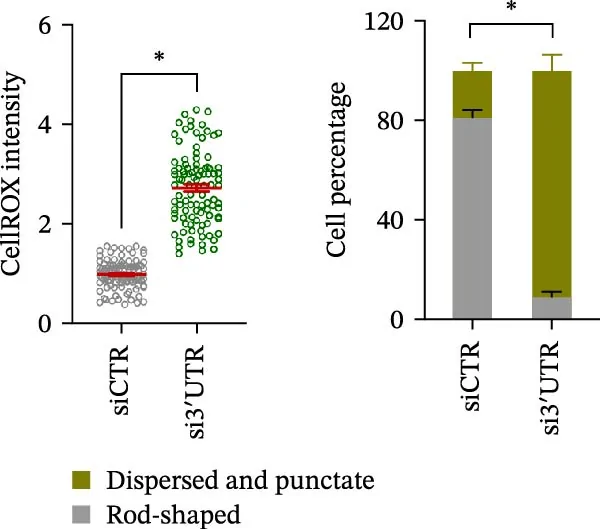
(G)

As displayed in Figure 5D, treatment of U251 cells with PF429242 for 48 hr led to a significant increase in mitochondrial ROS levels, accompanied by severely damaged mitochondrial structure. A moderate increase in fluorescence signal intensity was found after CellROX staining (mitochondrial ROS levels increased by +220%, 95% CI [180%, 260%], *p*  < 0.001, Cohen’s *d* = 2.35) (Figure 5D). Mitochondrial morphology was significantly altered, exhibiting a more dispersed or punctate structure compared to control cells, as observed in MitoTracker staining (mitochondrial fragmentation ratio: 65%, 95% CI [58%, 72%] vs. Control 15% [10%, 20%]) (Figure 5E). Similarly, ROS levels were moderately elevated in mitochondria after B166 knockdown by si3′UTR for 48 hr, and mitochondria were severely damaged, with numerous dispersed or punctate structures (mitochondrial ROS increase: +180%, 95% CI [150%, 210%], Cohen’s *d* = 2.05; mitochondrial fragmentation ratio: 58%, 95% CI [51%, 65%], OR = 8.2, 95% CI [5.1, 13.2]) (Figure 5F,G).

## 4. Discussion

The development of novel therapies to cure or attenuate GBM progression remains a critical priority, as existing treatments have demonstrated limited disease‐modifying effects. This study is the first to propose that targeting B166 to disrupt redox homeostasis in GBM may represent an effective therapeutic approach for this highly aggressive malignancy. In this study, the B166 protein level was significantly elevated in GBM tissues compared with both lower‐grade gliomas and normal brain tissues. Meanwhile, the prevalence of B166 elevation in GBM patients was significantly higher than that of PD‐L1 and EGFR, demonstrating that B166 may be a predictive and prognostic indicator. Importantly, the findings revealed that SREBP1‐a scavenged detrimental ROS by increasing B166 expression level in mitochondria and nuclei, which may represent a promising pathway for GBM therapy.

Fatty acid synthesis, uptake, and oxidation are markedly upregulated in GBM to meet the dramatic requirements of lipids and fuels that drive rapid proliferation and progression. SREBP1 is a crucial target of the EGFR/PI3K/Akt signaling pathway, which regulates lipid metabolism by elevating the expression levels of important enzymes, such as FASN, SCD, and ACC, as well as other critical proteins, including the mitochondrial citrate transporter SLC25A1 [27]. The results of the present study also indicated that SREBP1 expression level was upregulated in patients with GBM compared to those with lower‐grade gliomas, and its high expression level was closely associated with patients’ poor prognosis. The SREBP family has three isoforms, including SREBP1‐a, SREBP1‐c, and SREBP2, which are first synthesized as inactive precursors and then sequentially cleaved by site‐1 and site‐2 proteases to release their active N‐terminus, which can be translocated into the nucleus to regulate the transcription of other key enzymes and factors. Evidence from mouse hepatocytes indicated that the genes regulated by SREBP‐1 and SREBP‐2 overlap by only 11%, with the remaining 89% of the gene sets being distinct between the two isoforms [28]. A recent study demonstrated that SREBP‐1 was directly involved in the autophagy process of GBM cells, while the secondary role of SREBP‐2 in this process appeared noteworthy [29]. Consistently, the findings in GBM revealed that SREBP1‐a is the primary regulator of B166 and FASN, whereas the active N‐terminal SREBP1‐c or SREBP2 may have only minor transcriptional effects on B166 and FASN. Notably, this study revealed a previously unknown role of SREBP1‐a in scavenging detrimental ROS by increasing the expression levels of B166‐V1 and B166‐V5 proteins in both mitochondria and nuclei, thereby suggesting a connection between altered lipid metabolism and ROS clearance simultaneously.

During GBM malignancy, certain crucial regulators may, on one hand, modulate the expression levels of lipid synthesis‐related enzymes to provide abundant fatty acids for rapid proliferation, and on the other hand, improve antioxidant mechanisms to maintain ROS levels below the toxic thresholds for GBM cells. It has been reported that B166 also plays a fundamental role in scavenging excessive ROS and maintaining intracellular redox homeostasis [30]. The present study revealed significant correlations among B166, G6PD, and SREBP1 in GBM patients from both the PHG‐1 and TCGA GBM cohorts. Pharmacological inhibition of SREBP1 or genetic suppression of B166 markedly increased mitochondrial ROS levels, which in turn led to substantial damage to the mitochondrial structure, resulting in numerous dispersed and punctate textures. Moreover, inhibition of mitochondrial ROS could promote GBM progression and therapeutic resistance [31], further indicating that B166 may be an attractive target for GBM treatment.

Furthermore, overexpression of SREBP1‐aN confirmed the presence of the shorter isoform of B166 protein (B166‐V5) in the nucleus, which has an identical amino acid sequence and molecular weight to the longer isoform without MTS in the mitochondria (B166‐V1). B166‐V1 is initially synthesized as a precursor and subsequently cleaved to remove the MTS after translocation to the mitochondria. It then folds into the mature functional enzyme with three cysteine residues located at amino acids 100, 125, and 204, while Cys100 and Cys204 participate in the degradation of mitochondrial H_2_O_2_ (Figure 5G) [20, 32]. The detoxification capacity of B166‐V1 can be regulated via the S‐palmitoylation of Cys100, which is modulated by ABHD10 and attacks mitochondrial H_2_O_2_ as the nucleophilic active residue [33]. In the present study, B166‐V5 was upregulated to a greater extent versus B166‐V1 at both RNA and protein levels due to SREBP1‐aN overexpression. This study proposed a potential mechanism in GBM where increased lipid levels, primarily regulated by SREBP1‐a, led to both attenuation of elevated mitochondrial ROS via upregulation of B166‐V1 and elimination of excessive ROS in the nucleus via upregulation of B166‐V5. Therefore, B166 may be an effective target for disrupting normal mitochondrial function/morphology, providing multiple therapeutic options for GBM patients.

The present study revealed that B166, regulated by SREBP1‐a, is an attractive therapeutic target for GBM, while several questions remain. For instance, due to the lack of technical and experimental conditions, no in vivo intracranial orthotopic tumor model was established, and the currently more mature subcutaneous tumor model cannot fully simulate the growth and specific physiological conditions of orthotopic tumors, including lipid metabolism and ROS. In the future, an in vivo intracranial orthotopic tumor model will be developed to assess whether targeting B166 provides a therapeutic benefit for GBM treatment. In addition, although a significant correlation was identified between SREBP1 and B166, direct transcriptional regulation of B166 by SREBP1 was not confirmed. Future studies using chromatin immunoprecipitation or electrophoresis mobility shift assay are needed to confirm whether SREBP1 directly binds to the B166 promoter. Lastly, this study concentrated on B166‐mediated antioxidant effects, and additional antioxidant pathways (e.g., PRX3, glutathione peroxidase) were not explored. Hence, future studies are needed to perform ROC curve and sensitivity/specificity analyses for more rigorous comparison of B166 with existing biomarkers, such as PD‐L1 and EGFR.

## 5. Conclusions

This study identified a close correlation between B166 and SREBP1 in GBM patients. SREBP1, particularly SREBP1‐a, acts as the primary transcriptional regulator of B166. Notably, B166 mitochondrial isoform 1 and nuclear isoform 5, regulated by SREBP1‐a, may represent promising therapeutic targets for GBM. By enhancing ROS elimination, these isoforms contribute to redox homeostasis in GBM cells. These findings highlight the potential of B166 as a novel biomarker and a therapeutic target, warranting further investigation to improve treatment strategies for GBM.

**Nomenclature**
ACC: Acetyl‐CoA carboxylases
B166‐V1: B166 transcript variant 1
BRCA: Breast cancer
CHLO: Cholangiocarcinoma
DFS: Disease‐free survival
DMEM: Dulbecco’s modified Eagle’s medium
ESCA: Endometrioid carcinoma
FASN: Fatty acid synthase
G6PD: Glucose‐6‐phosphate dehydrogenase
GBM: Glioblastoma multiforme
IHC: Immunohistochemistry
KIRP: Renal chromophobia
LIHC: Liver cancer
MTS: Mitochondrial targeting sequence
OS: Overall survival
PBS: Phosphate‐buffered saline
PRX: Peroxiredoxin
ROS: Reactive oxygen species
SCD: Stearoyl‐CoA desaturase
siRNA: Small interfering RNAs
SREBPs: Sterol regulatory element‐binding proteins
TMA: Tissue microarray
WHO: World Health Organization

## Author Contributions

All authors, including Cindy Cheung, Weiqiang Liu, Bingwu Wang, and Fanhua Kong, made a significant contribution to the study, including in the conception, study design, execution, data acquisition, analysis, and interpretation. All authors contributed to drafting, revising, or critically reviewing the article.

## Funding

This study was supported by a Start‐Up Grant for Senior Research Associate from the First Affiliated Hospital of Shandong Second Medical University and the Human Spine Biomechanics Laboratory of the First Affiliated Hospital, Shandong Second Medical University. This study was also supported by the Natural Science Foundation of Shandong Province (Grant ZR2022MH273).

## Disclosure

All authors have approved the final version for publication, agreed on the journal to which the article was submitted, and are accountable for all aspects of the research.

Note: The first version of this manuscript was published as a preprint by Research Square. The title is “Modulation of antioxidant enzyme B166 isoforms 1 and 5 expressions by SREBP1‐a links ROS elimination to lipid metabolism in glioblastoma cells,” and the DOI can be obtained via the following link: https://doi.org/10.21203/rs.3.rs-3542956/v1. This is an updated version.

## Ethics Statement

All procedures involving human participants in this study were approved by the Institutional Review Board of the First Affiliated Hospital Affiliated to Shandong Second Medical University (Approval Number: KYLL20240219‐2). Informed consent was granted by the patients before the use of their clinical specimens for research purposes.

## Consent

The authors have nothing to report.

## Conflicts of Interest

The authors declare no conflicts of interest.

## Supporting Information

Additional supporting information can be found online in the Supporting Information section.

## Supporting information


**Supporting Information** Table S1 Glioma tissue microarray (TMA) cohort PHG‐1. Table S2 Primer sequences used in quantitative real‐time PCR. Table S3 The normalized B166 expression of the GBM tissues and cortex tissues. Table S4 The normalized B166 expression and overall‐ survival outcomes of the patients in TCGA LGG GBM cohort. Table S5 The histological types, IDH1 mutation statuses and MGMT methylation statuses of the patients in TCGA LGG GBM cohort. **Table S6** The comprehensive information of IDH1 mutation of the patients in TCGA LGG GBM cohort.

## Data Availability

Data are provided within the manuscript or in the supporting information files.
