# Supplementary material for: Identification of the Antioxidant Enzyme B166 as a Novel Biomarker for Patients With Glioblastoma Multiforme
Source: Anal Cell Pathol (Amst). 2026 Feb 17;2026:6120748. doi: 10.1155/ancp/6120748 (PMC13580799; doi:10.1155/ancp/6120748)
Supplement: Supplementary file 1 [file ANCP-2026-6120748-s001.docx]

**Table S1 Glioma tissue microarray (TMA) cohort PHG-1**

| **Tumor patient tissue number** | **Location of the tissue point** | **Survival status of patients** | **Tumour recurrent status** | **Disease-free survival (months)** | **Overall survival (months)** | **Pathologic grades** | **Gender** | **Age at diagnosis in years** | **Primary or metastatic tumour** | **Tumour Pathology Types** | **Tumour location** | **Patient's surgery time** |
| --- | --- | --- | --- | --- | --- | --- | --- | --- | --- | --- | --- | --- |
| P01A0684 | J04 | Death | Recurrent | 10 | 10 | IV | Male | 45 | Primary | Glioma | Frontal | 20110729 |
| P01A0681 | I13 | Death | Recurrent | 10 | 10 | IV | Male | 62 | Primary | Glioma | Others | 20110502 |
| P01A0670 | G02 | Death | Recurrent | 10 | 10 | IV | Male | 60 | Primary | Glioma | Occipital | 20100506 |
| P01A0530 | B10 | Survival | Recurrent | 59 | 105 | II | Female | 49 | Primary | Glioma | Others | 20081016 |
| P01A0531 | B11 | Survival | Non-recurrent | 105 | 105 | II | Male | 61 | Primary | Glioma | Temporal | 20081022 |
| P01A0555 | C18 | Death | Recurrent | 58 | 66 | IV | Female | 41 | Primary | Glioma | Temporal | 20090413 |
| P02A0004 | H09 | Survival | Non-recurrent | 79 | 79 | III | Female | 59 | Primary | Glioma | Others | 20101207 |
| P01A0639 | I08 | Survival | Non-recurrent | 75 | 75 | II | Male | 48 | Primary | Glioma | Temporal | 20110404 |
| P01A0666 | E01 | Death | Recurrent | 41 | 50 | IV | Male | 41 | Primary | Glioma | Temporal | 20090902 |
| P01A0558 | D02 | Survival | Recurrent | 48 | 99 | III | Male | 68 | Primary | Glioma | Temporal | 20090424 |
| P01A0538 | B18 | Survival | Non-recurrent | 103 | 103 | I | Female | 37 | Primary | Glioma | Multifocal | 20081225 |
| P01A0598 | F10 | Survival | Recurrent | 41 | 88 | II | Male | 32 | Primary | Glioma | Ventricle | 20100312 |
| P01A0624 | H04 | Survival | Recurrent | 33 | 81 | III | Male | 47 | Primary | Glioma | Frontal | 20101020 |
| P01A0608 | G03 | Death | Recurrent | 33 | 33 | III | Female | 33 | Primary | Glioma | Frontal | 20100506 |
| P01A0519 | A17 | Death | Recurrent | 17 | 72 | III | Female | 56 | Primary | Glioma | Temporal | 20080722 |
| P01A0575 | E03 | Survival | Non-recurrent | 94 | 94 | II | Female | 36 | Primary | Glioma | Frontal | 20090907 |
| P01A0585 | E14 | Survival | Non-recurrent | 92 | 92 | III | Male | 35 | Primary | Glioma | Frontal | 20091109 |
| P01A0604 | F16 | Death | Recurrent | 35 | 55 | III | Female | 45 | Primary | Glioma | Frontal | 20100415 |
| P01A0680 | H17 | Death | Recurrent | 6 | 6 | IV | Male | 78 | Primary | Glioma | Frontal | 20110131 |
| P01A0685 | J10 | Death | Recurrent | 14 | 14 | IV | Male | 41 | Primary | Glioma | Temporal | 20110824 |
| P01A0541 | C03 | Survival | Non-recurrent | 102 | 102 | II | Male | 6 | Primary | Glioma | Frontal | 20090120 |
| P01A0547 | C09 | Survival | Non-recurrent | 101 | 101 | I | Female | 61 | Primary | Glioma | Occipital | 20090225 |
| P01A0560 | D04 | Death | Recurrent | 55 | 59 | II | Male | 63 | Primary | Glioma | Temporal | 20090518 |
| P01A0640 | I09 | Death | Recurrent | 18 | 18 | III | Male | 30 | Primary | Glioma | Multifocal | 20110418 |
| P01A0510 | A05 | Survival | Recurrent | 27 | 112 | III | Female | 63 | Primary | Glioma | Multifocal | 20080304 |
| P01A0537 | B17 | Survival | Non-recurrent | 103 | 103 | II | Male | 66 | Primary | Glioma | Temporal | 20081218 |
| P01A0676 | H12 | Death | Recurrent | 23 | 34 | IV | Male | 65 | Primary | Glioma | Temporal | 20101227 |
| P01A0682 | I16 | Death | Recurrent | 19 | 19 | III | Male | 67 | Primary | Glioma | Temporal | 20110601 |
| P01A0630 | H16 | Death | Recurrent | 26 | 26 | III | Male | 49 | Primary | Glioma | Occipital | 20110128 |
| P01A0614 | G11 | Survival | Non-recurrent | 85 | 85 | I | Female | 49 | Primary | Glioma | Frontal | 20100627 |
| P01A0677 | H13 | Death | Recurrent | 17 | 17 | IV | Male | 9 | Primary | Glioma | Ventricle | 20110105 |
| P01A0678 | H14 | Death | Recurrent | 12 | 12 | IV | Male | 41 | Primary | Glioma | Temporal | 20110112 |
| P01A0506 | A01 | Survival | Non-recurrent | 113 | 113 | II | Female | 37 | Primary | Glioma | Multifocal | 20080202 |
| P01A0548 | C10 | Survival | Non-recurrent | 100 | 100 | II | Male | 34 | Primary | Glioma | Multifocal | 20090304 |
| P01A0559 | D03 | Survival | Recurrent | 49 | 98 | II | Male | 31 | Primary | Glioma | Others | 20090504 |
| P01A0563 | D07 | Survival | Recurrent | 55 | 97 | II | Male | 57 | Primary | Glioma | Frontal | 20090604 |
| P01A0658 | J14 | Death | Recurrent | 37 | 37 | III | Male | 57 | Primary | Glioma | Temporal | 20111006 |
| P01A0660 | J17 | Survival | Recurrent | 69 | 69 | III | Female | 36 | Primary | Glioma | Multifocal | 20111011 |
| P01A0515 | A13 | Survival | Non-recurrent | 109 | 109 | III | Male | 17 | Primary | Glioma | Frontal | 20080604 |
| P01A0602 | F14 | Survival | Non-recurrent | 87 | 87 | II | Male | 3 | Primary | Glioma | Others | 20100408 |
| P01A0643 | I12 | Survival | Recurrent | 75 | 75 | III | Female | 26 | Primary | Glioma | Frontal | 20110427 |
| P01A0524 | B04 | Death | Recurrent | 83 | 83 | II | Male | 66 | Primary | Glioma | Frontal | 20080912 |
| P01A0542 | C04 | Survival | Non-recurrent | 102 | 102 | I | Male | 5 | Primary | Glioma | Others | 20090123 |
| P01A0572 | D15 | Survival | Recurrent | 54 | 96 | II | Female | 42 | Primary | Glioma | parietal | 20090723 |
| P01A0597 | F09 | Survival | Non-recurrent | 88 | 88 | II | Female | 36 | Primary | Glioma | Temporal | 20100309 |
| P01A0637 | I06 | Survival | Non-recurrent | 76 | 76 | II | Male | 54 | Primary | Glioma | Others | 20110318 |
| P01A0651 | J06 | Death | Recurrent | 11 | 11 | III | Female | 69 | Primary | Glioma | Frontal | 20110801 |
| P01A0653 | J07 | Survival | Non-recurrent | 71 | 71 | II | Female | 19 | Primary | Glioma | Temporal | 20110801 |
| P01A0652 | J08 | Survival | Non-recurrent | 71 | 71 | II | Male | 49 | Primary | Glioma | parietal | 20110802 |
| P01A0686 | J16 | Death | Recurrent | 27 | 27 | IV | Female | 28 | Primary | Glioma | Frontal | 20111010 |
| P01A0512 | A09 | Survival | Non-recurrent | 111 | 111 | I | Female | 11 | Primary | Glioma | parietal | 20080425 |
| P01A0539 | C01 | Survival | Non-recurrent | 102 | 102 | I | Male | 4 | Primary | Glioma | Multifocal | 20090112 |
| P01A0545 | C06 | Survival | Non-recurrent | 101 | 101 | I | Male | 32 | Primary | Glioma | Temporal | 20090216 |
| P01A0577 | E06 | Survival | Non-recurrent | 93 | 93 | II | Male | 36 | Primary | Glioma | Occipital | 20091002 |
| P01A0613 | G10 | Survival | Non-recurrent | 85 | 85 | II | Male | 36 | Primary | Glioma | Occipital | 20100625 |
| P01A0616 | G13 | Survival | Non-recurrent | 84 | 84 | II | Male | 19 | Primary | Glioma | Others | 20100713 |
| P01A0621 | H01 | Survival | Non-recurrent | 82 | 82 | III | Male | 33 | Primary | Glioma | parietal | 20100919 |
| P01A0628 | H08 | Death | Recurrent | 34 | 34 | III | Female | 58 | Primary | Glioma | Temporal | 20101124 |
| P01A0646 | I17 | Survival | Non-recurrent | 72 | 72 | I | Male | 30 | Primary | Glioma | Ventricle | 20110713 |
| P01A0683 | J03 | Death | Recurrent | 9 | 9 | IV | Male | 63 | Primary | Glioma | Others | 20110729 |
| P01A0536 | B16 | Survival | Non-recurrent | 103 | 103 | I | Male | 12 | Primary | Glioma | Others | 20081211 |
| P01A0642 | I11 | Death | Recurrent | 23 | 23 | III | Male | 52 | Primary | Glioma | Temporal | 20110426 |
| P01A0521 | B01 | Survival | Recurrent | 34 | 108 | II | Male | 80 | Primary | Glioma | Temporal | 20080728 |
| P01A0528 | B08 | Survival | Recurrent | 72 | 105 | III | Male | 29 | Primary | Glioma | Frontal | 20081008 |
| P01A0532 | B12 | Survival | Recurrent | 52 | 105 | II | Male | 44 | Primary | Glioma | Temporal | 20081031 |
| P01A0557 | D01 | Survival | Non-recurrent | 99 | 99 | II | Male | 41 | Primary | Glioma | Frontal | 20090421 |
| P01A0580 | E09 | Survival | Non-recurrent | 93 | 93 | III | Female | 42 | Primary | Glioma | Ventricle | 20091030 |
| P01A0589 | F01 | Survival | Recurrent | 43 | 91 | III | Female | 47 | Primary | Glioma | Frontal | 20091221 |
| P01A0592 | F04 | Death | Recurrent | 21 | 48 | III | Male | 42 | Primary | Glioma | Temporal | 20100125 |
| P01A0609 | G05 | Survival | Non-recurrent | 86 | 86 | I | Female | 14 | Primary | Glioma | Multifocal | 20100518 |
| P01A0610 | G06 | Survival | Non-recurrent | 86 | 86 | II | Male | 28 | Primary | Glioma | Frontal | 20100525 |
| P01A0623 | H03 | Survival | Non-recurrent | 81 | 81 | I | Female | 38 | Primary | Glioma | Others | 20101006 |
| P01A0629 | H10 | Death | Recurrent | 23 | 23 | III | Female | 30 | Primary | Glioma | Temporal | 20101209 |
| P01A0634 | I02 | Survival | Non-recurrent | 77 | 77 | II | Male | 40 | Primary | Glioma | Temporal | 20110223 |
| P01A0635 | I03 | Survival | Recurrent | 76 | 76 | II | Male | 41 | Primary | Glioma | Frontal | 20110310 |
| P01A0644 | I14 | Survival | Non-recurrent | 74 | 74 | II | Male | 56 | Primary | Glioma | Others | 20110509 |
| P01A0649 | J02 | Death | Recurrent | 26 | 26 | II | Male | 41 | Primary | Glioma | Temporal | 20110728 |
| P01A0654 | J09 | Survival | Non-recurrent | 71 | 71 | I | Female | 66 | Primary | Glioma | Others | 20110815 |
| P01A0655 | J11 | Death | Recurrent | 21 | 21 | III | Male | 56 | Primary | Glioma | Multifocal | 20110909 |
| P01A0522 | B02 | Survival | Non-recurrent | 108 | 108 | II | Female | 28 | Primary | Glioma | Temporal | 20080730 |
| P01A0552 | C14 | Survival | Non-recurrent | 100 | 100 | II | Male | 46 | Primary | Glioma | parietal | 20090327 |
| P01A0638 | I07 | Survival | Non-recurrent | 76 | 76 | II | Female | 32 | Primary | Glioma | Frontal | 20110328 |
| P01A0517 | A15 | Survival | Non-recurrent | 108 | 108 | I | Female | 34 | Primary | Glioma | Temporal | 20080702 |
| P01A0591 | F03 | Death | Recurrent | 23 | 44 | III | Female | 37 | Primary | Glioma | Temporal | 20100119 |
| P01A0687 | I05 | Survival | Non-recurrent | 97 | 97 | I | Female | 6 | Primary | Glioma | Others | 20090619 |
| P01A0645 | I15 | Survival | Non-recurrent | 74 | 74 | II | Male | 15 | Primary | Glioma | Frontal | 20110520 |
| P01A0578 | E07 | Survival | Recurrent | 13 | 93 | II | Female | 23 | Primary | Glioma | Temporal | 20091012 |
| P01A0586 | E16 | Survival | Recurrent | 26 | 92 | II | Male | 40 | Primary | Glioma | Ventricle | 20091123 |

**Table S2 Primer sequences used in quantitative real-time PCR**

|  | Sequence Name | Sequence |
| --- | --- | --- |
| B166 | B166(F) | TGGGAAGGAGACAGACTTAT |
|  | B166(R) | TTCACTATGCCATCCTGTAC |
| SLC7A11 | SLC7A11(F) | TTAGTACAGGGATTGGCTTC |
|  | SLC7A11(R) | CTGGTTATTTTCTCCGACATT |
| SLC3A2 | SLC3A2(F) | GGACCTTACTCCCAACTAC |
|  | SLC3A2(R) | TCAGATTCTCTATGTCCCGA |
| SLC7A5 | SLC7A5(F) | GCACCACCATCTCCAAATC |
|  | SLC7A5(R) | CGATGTACTGCGATGAAGG |
| Nrf2 | Nrf2(F) | GAGAGCCCAGTCTTCATTGC |
|  | Nrf2(R) | TTGGCTTCTGGACTTGGAAC |
| NQO1 | NQO1(F) | TGAAGAAGAAAGGATGGGAGGT |
|  | NQO1(R) | GGCCTTCTTTATAAGCCAGAACA |
| Gclc | Gclc(F) | GGAAGTGGATGTGGACACCAGA |
|  | Gclc(R) | GCTTGTAGTCAGGATGGTTTGCG |
| GPX4 | GPX4(F) | ACAAGAACGGCTGCGTGGTGAA |
|  | GPX4(R) | GCCACACACTTGTGGAGCTAGA |
| Prdx1 | Prdx1(F) | CGCACCATTGCTCAGGAT |
|  | Prdx1(R) | GCCAACAGGGAGGTCATT |
| HMOX1 | HMOX1(F) | CCAGGCAGAGAATGCTGAGTTC |
|  | HMOX1(R) | AAGACTGGGCTCTCCTTGTTGC |
| TXN | TXN(F) | CTTGGACGCTGCAGGTGATA |
|  | TXN(R) | TCTGAAGCAACATCCTGACAGT |
| Gclm | Gclm(F) | TCAACCCAGATTTGGTCAGGGAGT |
|  | Gclm(R) | TCCAGCTGTGCAACTCCAAGGA |
| G6PD | G6PD(F) | GGCAACAGATACAAGAACG |
|  | G6PD(R) | GCAGTGGGGTGAAAATACG |

**Table S3 The normalized B166 expression of the GBM tissues and cortex tissues**

| **Sample** | **Study** | **B166** | **Primary disease or tissue** | **Sample type** |
| --- | --- | --- | --- | --- |
| GTEX-X585-3026-SM-46MWF | GTEX | 8.928 | Cortex | Normal Tissue |
| GTEX-14BMV-3026-SM-5S2PQ | GTEX | 8.672 | Cortex | Normal Tissue |
| GTEX-WVLH-3026-SM-3MJG9 | GTEX | 8.655 | Cortex | Normal Tissue |
| GTEX-WL46-2926-SM-3LK82 | GTEX | 8.6 | Cortex | Normal Tissue |
| GTEX-X4XX-3026-SM-3NMB2 | GTEX | 8.57 | Cortex | Normal Tissue |
| GTEX-13SLX-3126-SM-5S2Q5 | GTEX | 8.552 | Cortex | Normal Tissue |
| GTEX-12WSC-3026-SM-5GCNF | GTEX | 8.539 | Cortex | Normal Tissue |
| GTEX-13VXU-2926-SM-5LU5C | GTEX | 8.529 | Cortex | Normal Tissue |
| GTEX-11DXY-3226-SM-5GIDE | GTEX | 8.516 | Cortex | Normal Tissue |
| GTEX-WWYW-3126-SM-3NB39 | GTEX | 8.505 | Cortex | Normal Tissue |
| GTEX-13OW6-3026-SM-5J2MI | GTEX | 8.504 | Cortex | Normal Tissue |
| GTEX-WZTO-2926-SM-3NM9I | GTEX | 8.499 | Cortex | Normal Tissue |
| GTEX-1192X-3126-SM-5N9BY | GTEX | 8.497 | Cortex | Normal Tissue |
| GTEX-WHSE-3026-SM-3P5ZH | GTEX | 8.496 | Cortex | Normal Tissue |
| GTEX-1313W-3126-SM-5LZUI | GTEX | 8.493 | Cortex | Normal Tissue |
| GTEX-13NYC-2826-SM-5K7WR | GTEX | 8.483 | Cortex | Normal Tissue |
| GTEX-117XS-3026-SM-5N9CA | GTEX | 8.476 | Cortex | Normal Tissue |
| GTEX-145MG-3026-SM-5RQJA | GTEX | 8.444 | Cortex | Normal Tissue |
| GTEX-13O3Q-2926-SM-5KM45 | GTEX | 8.443 | Cortex | Normal Tissue |
| GTEX-14ASI-3026-SM-5S2PN | GTEX | 8.425 | Cortex | Normal Tissue |
| GTEX-12ZZY-3026-SM-5GCOU | GTEX | 8.424 | Cortex | Normal Tissue |
| GTEX-12ZZX-2926-SM-5GCOQ | GTEX | 8.415 | Cortex | Normal Tissue |
| GTEX-ZYFD-3026-SM-5E44C | GTEX | 8.407 | Cortex | Normal Tissue |
| GTEX-11GSO-2926-SM-5HL73 | GTEX | 8.395 | Cortex | Normal Tissue |
| GTEX-12126-1026-SM-5P9JJ | GTEX | 8.391 | Cortex | Normal Tissue |
| GTEX-NPJ8-1526-SM-2D7VU | GTEX | 8.389 | Cortex | Normal Tissue |
| GTEX-13FHO-3026-SM-5J1O9 | GTEX | 8.385 | Cortex | Normal Tissue |
| GTEX-Z93S-2926-SM-57WB9 | GTEX | 8.374 | Cortex | Normal Tissue |
| GTEX-13NYS-3126-SM-5KLYV | GTEX | 8.358 | Cortex | Normal Tissue |
| GTEX-13FLW-1426-SM-5K7YE | GTEX | 8.357 | Cortex | Normal Tissue |
| GTEX-145MH-3026-SM-5Q5DZ | GTEX | 8.355 | Cortex | Normal Tissue |
| GTEX-145LS-3126-SM-5Q5BY | GTEX | 8.354 | Cortex | Normal Tissue |
| GTEX-13OVH-3026-SM-5MR4N | GTEX | 8.349 | Cortex | Normal Tissue |
| GTEX-ZE9C-3026-SM-4WKHB | GTEX | 8.331 | Cortex | Normal Tissue |
| GTEX-ZE7O-3126-SM-5HL5X | GTEX | 8.329 | Cortex | Normal Tissue |
| GTEX-ZAK1-3026-SM-5S2MJ | GTEX | 8.328 | Cortex | Normal Tissue |
| GTEX-Q2AG-2926-SM-2HMJ3 | GTEX | 8.316 | Cortex | Normal Tissue |
| GTEX-Y8DK-0826-SM-4TT3T | GTEX | 8.309 | Cortex | Normal Tissue |
| GTEX-T6MN-2626-SM-32PMQ | GTEX | 8.304 | Cortex | Normal Tissue |
| GTEX-13NYB-3026-SM-5IJD7 | GTEX | 8.302 | Cortex | Normal Tissue |
| GTEX-11NUK-2926-SM-5A5MD | GTEX | 8.301 | Cortex | Normal Tissue |
| GTEX-1399T-3026-SM-5KLZC | GTEX | 8.294 | Cortex | Normal Tissue |
| GTEX-XOTO-3026-SM-4B65M | GTEX | 8.293 | Cortex | Normal Tissue |
| GTEX-XLM4-3026-SM-4AT6L | GTEX | 8.29 | Cortex | Normal Tissue |
| GTEX-NPJ8-1526-SM-26GMH | GTEX | 8.283 | Cortex | Normal Tissue |
| GTEX-ZVT3-3026-SM-5E43N | GTEX | 8.259 | Cortex | Normal Tissue |
| GTEX-ZUA1-3026-SM-59HJC | GTEX | 8.246 | Cortex | Normal Tissue |
| GTEX-R55F-1326-SM-5S2V4 | GTEX | 8.245 | Cortex | Normal Tissue |
| GTEX-13O3O-3126-SM-5KM3H | GTEX | 8.235 | Cortex | Normal Tissue |
| GTEX-12ZZW-2926-SM-5LZUP | GTEX | 8.233 | Cortex | Normal Tissue |
| GTEX-RNOR-2326-SM-2TF4I | GTEX | 8.233 | Cortex | Normal Tissue |
| GTEX-131YS-3126-SM-5KLYT | GTEX | 8.211 | Cortex | Normal Tissue |
| GTEX-144GL-3026-SM-5Q5CW | GTEX | 8.21 | Cortex | Normal Tissue |
| GTEX-ZF28-3026-SM-4WKHP | GTEX | 8.209 | Cortex | Normal Tissue |
| GTEX-132Q8-3026-SM-5PNVG | GTEX | 8.185 | Cortex | Normal Tissue |
| GTEX-11DXW-1126-SM-5H12Q | GTEX | 8.157 | Cortex | Normal Tissue |
| GTEX-11ONC-2926-SM-5P9JM | GTEX | 8.156 | Cortex | Normal Tissue |
| GTEX-11ZUS-2926-SM-5FQSL | GTEX | 8.154 | Cortex | Normal Tissue |
| GTEX-RU72-3026-SM-5SI7Y | GTEX | 8.153 | Cortex | Normal Tissue |
| GTEX-145MF-2726-SM-5O995 | GTEX | 8.137 | Cortex | Normal Tissue |
| GTEX-T2IS-3026-SM-32QPM | GTEX | 8.114 | Cortex | Normal Tissue |
| GTEX-R55F-1326-SM-2TF5F | GTEX | 8.082 | Cortex | Normal Tissue |
| GTEX-N7MT-1126-SM-2YUNQ | GTEX | 8.062 | Cortex | Normal Tissue |
| GTEX-1128S-2726-SM-5H12C | GTEX | 8.051 | Cortex | Normal Tissue |
| GTEX-ZAJG-3126-SM-5HL9J | GTEX | 8.048 | Cortex | Normal Tissue |
| GTEX-139UC-3126-SM-5J1OY | GTEX | 8.047 | Cortex | Normal Tissue |
| GTEX-QVUS-2826-SM-3GADB | GTEX | 8.038 | Cortex | Normal Tissue |
| GTEX-T5JC-2426-SM-3NMDB | GTEX | 7.991 | Cortex | Normal Tissue |
| GTEX-144FL-3026-SM-5O99C | GTEX | 7.988 | Cortex | Normal Tissue |
| GTEX-13FHP-3026-SM-5IJBS | GTEX | 7.977 | Cortex | Normal Tissue |
| GTEX-13OVJ-2826-SM-5L3GW | GTEX | 7.971 | Cortex | Normal Tissue |
| GTEX-11EI6-3026-SM-5GZZO | GTEX | 7.955 | Cortex | Normal Tissue |
| GTEX-111FC-3126-SM-5GZZ2 | GTEX | 7.952 | Cortex | Normal Tissue |
| GTEX-11NV4-2126-SM-5N9DS | GTEX | 7.949 | Cortex | Normal Tissue |
| GTEX-11GS4-3126-SM-5A5LH | GTEX | 7.948 | Cortex | Normal Tissue |
| GTEX-12ZZZ-3026-SM-5BC67 | GTEX | 7.943 | Cortex | Normal Tissue |
| GTEX-14A5I-2926-SM-5Q5CQ | GTEX | 7.943 | Cortex | Normal Tissue |
| GTEX-13QIC-2926-SM-5J2NF | GTEX | 7.934 | Cortex | Normal Tissue |
| GTEX-13X6J-3026-SM-5Q5CU | GTEX | 7.928 | Cortex | Normal Tissue |
| GTEX-12WSH-3026-SM-5CVNI | GTEX | 7.897 | Cortex | Normal Tissue |
| GTEX-11EMC-3226-SM-5EGKW | GTEX | 7.873 | Cortex | Normal Tissue |
| GTEX-PVOW-2526-SM-2XCF7 | GTEX | 7.823 | Cortex | Normal Tissue |
| GTEX-13PL6-3126-SM-5LUAR | GTEX | 7.814 | Cortex | Normal Tissue |
| GTEX-12WSD-3126-SM-5HL7P | GTEX | 7.812 | Cortex | Normal Tissue |
| GTEX-ZYY3-3126-SM-5SI9L | GTEX | 7.777 | Cortex | Normal Tissue |
| GTEX-N7MS-2425-SM-26GMD | GTEX | 7.743 | Cortex | Normal Tissue |
| GTEX-13S7M-3126-SM-5RQJQ | GTEX | 7.737 | Cortex | Normal Tissue |
| GTEX-ZZPT-3026-SM-5GZXH | GTEX | 7.73 | Cortex | Normal Tissue |
| GTEX-1117F-3226-SM-5N9CT | GTEX | 7.714 | Cortex | Normal Tissue |
| GTEX-11WQK-3026-SM-5EQL6 | GTEX | 7.713 | Cortex | Normal Tissue |
| GTEX-13G51-3026-SM-5IJB8 | GTEX | 7.681 | Cortex | Normal Tissue |
| GTEX-11TTK-2926-SM-5PNYP | GTEX | 7.606 | Cortex | Normal Tissue |
| GTEX-11GSP-3226-SM-5986O | GTEX | 7.561 | Cortex | Normal Tissue |
| GTEX-XMD1-2926-SM-4AT42 | GTEX | 7.53 | Cortex | Normal Tissue |
| GTEX-NPJ7-2726-SM-2I3FT | GTEX | 7.522 | Cortex | Normal Tissue |
| GTEX-TSE9-3026-SM-3DB76 | GTEX | 7.46 | Cortex | Normal Tissue |
| GTEX-13IVO-2926-SM-5L3CZ | GTEX | 7.39 | Cortex | Normal Tissue |
| GTEX-QDT8-2926-SM-32PKC | GTEX | 7.367 | Cortex | Normal Tissue |
| GTEX-11TUW-3126-SM-5GU4Y | GTEX | 7.301 | Cortex | Normal Tissue |
| GTEX-13OW7-3026-SM-5L3GY | GTEX | 7.299 | Cortex | Normal Tissue |
| GTEX-12696-2926-SM-5FQTG | GTEX | 7.273 | Cortex | Normal Tissue |
| GTEX-1445S-3026-SM-5O9BR | GTEX | 7.2 | Cortex | Normal Tissue |
| GTEX-11O72-2926-SM-5BC4V | GTEX | 7.144 | Cortex | Normal Tissue |
| GTEX-12WSA-2926-SM-5EQ4D | GTEX | 7.046 | Cortex | Normal Tissue |
| GTEX-NPJ8-1526-SM-26GMY | GTEX | 5.057 | Cortex | Normal Tissue |
| TCGA-76-4929-01 | TCGA | 9.536 | GBM | Primary Tumor |
| TCGA-27-2526-01 | TCGA | 9.454 | GBM | Primary Tumor |
| TCGA-28-5213-01 | TCGA | 9.363 | GBM | Primary Tumor |
| TCGA-12-3652-01 | TCGA | 9.346 | GBM | Primary Tumor |
| TCGA-06-5411-01 | TCGA | 9.181 | GBM | Primary Tumor |
| TCGA-27-1832-01 | TCGA | 9.151 | GBM | Primary Tumor |
| TCGA-41-2572-01 | TCGA | 9.146 | GBM | Primary Tumor |
| TCGA-06-0178-01 | TCGA | 9.108 | GBM | Primary Tumor |
| TCGA-27-1831-01 | TCGA | 9.101 | GBM | Primary Tumor |
| TCGA-06-5416-01 | TCGA | 9.081 | GBM | Primary Tumor |
| TCGA-06-0219-01 | TCGA | 9.042 | GBM | Primary Tumor |
| TCGA-26-5132-01 | TCGA | 9.019 | GBM | Primary Tumor |
| TCGA-76-4931-01 | TCGA | 9.017 | GBM | Primary Tumor |
| TCGA-27-2519-01 | TCGA | 9.013 | GBM | Primary Tumor |
| TCGA-08-0386-01 | TCGA | 8.985 | GBM | Primary Tumor |
| TCGA-14-1829-01 | TCGA | 8.983 | GBM | Primary Tumor |
| TCGA-28-5220-01 | TCGA | 8.981 | GBM | Primary Tumor |
| TCGA-06-0749-01 | TCGA | 8.973 | GBM | Primary Tumor |
| TCGA-06-2567-01 | TCGA | 8.959 | GBM | Primary Tumor |
| TCGA-12-5295-01 | TCGA | 8.943 | GBM | Primary Tumor |
| TCGA-06-2569-01 | TCGA | 8.915 | GBM | Primary Tumor |
| TCGA-06-5859-01 | TCGA | 8.914 | GBM | Primary Tumor |
| TCGA-06-0168-01 | TCGA | 8.902 | GBM | Primary Tumor |
| TCGA-26-5136-01 | TCGA | 8.882 | GBM | Primary Tumor |
| TCGA-27-2521-01 | TCGA | 8.834 | GBM | Primary Tumor |
| TCGA-76-4925-01 | TCGA | 8.816 | GBM | Primary Tumor |
| TCGA-28-2499-01 | TCGA | 8.807 | GBM | Primary Tumor |
| TCGA-06-0745-01 | TCGA | 8.795 | GBM | Primary Tumor |
| TCGA-14-2554-01 | TCGA | 8.795 | GBM | Primary Tumor |
| TCGA-27-2524-01 | TCGA | 8.781 | GBM | Primary Tumor |
| TCGA-28-5204-01 | TCGA | 8.776 | GBM | Primary Tumor |
| TCGA-19-2629-01 | TCGA | 8.774 | GBM | Primary Tumor |
| TCGA-06-5408-01 | TCGA | 8.771 | GBM | Primary Tumor |
| TCGA-06-0132-01 | TCGA | 8.769 | GBM | Primary Tumor |
| TCGA-06-5415-01 | TCGA | 8.765 | GBM | Primary Tumor |
| TCGA-32-2616-01 | TCGA | 8.757 | GBM | Primary Tumor |
| TCGA-06-5413-01 | TCGA | 8.745 | GBM | Primary Tumor |
| TCGA-12-3650-01 | TCGA | 8.735 | GBM | Primary Tumor |
| TCGA-76-4932-01 | TCGA | 8.712 | GBM | Primary Tumor |
| TCGA-06-5418-01 | TCGA | 8.708 | GBM | Primary Tumor |
| TCGA-26-5133-01 | TCGA | 8.7 | GBM | Primary Tumor |
| TCGA-12-5299-01 | TCGA | 8.696 | GBM | Primary Tumor |
| TCGA-14-0787-01 | TCGA | 8.696 | GBM | Primary Tumor |
| TCGA-06-1804-01 | TCGA | 8.691 | GBM | Primary Tumor |
| TCGA-06-0750-01 | TCGA | 8.67 | GBM | Primary Tumor |
| TCGA-06-0744-01 | TCGA | 8.661 | GBM | Primary Tumor |
| TCGA-06-0882-01 | TCGA | 8.646 | GBM | Primary Tumor |
| TCGA-14-0790-01 | TCGA | 8.624 | GBM | Primary Tumor |
| TCGA-32-1980-01 | TCGA | 8.606 | GBM | Primary Tumor |
| TCGA-06-0743-01 | TCGA | 8.598 | GBM | Primary Tumor |
| TCGA-14-1825-01 | TCGA | 8.591 | GBM | Primary Tumor |
| TCGA-26-1442-01 | TCGA | 8.591 | GBM | Primary Tumor |
| TCGA-28-5208-01 | TCGA | 8.587 | GBM | Primary Tumor |
| TCGA-06-2558-01 | TCGA | 8.577 | GBM | Primary Tumor |
| TCGA-28-2513-01 | TCGA | 8.576 | GBM | Primary Tumor |
| TCGA-32-1970-01 | TCGA | 8.573 | GBM | Primary Tumor |
| TCGA-28-2510-01 | TCGA | 8.569 | GBM | Primary Tumor |
| TCGA-19-2624-01 | TCGA | 8.566 | GBM | Primary Tumor |
| TCGA-76-4927-01 | TCGA | 8.561 | GBM | Primary Tumor |
| TCGA-28-1747-01 | TCGA | 8.557 | GBM | Primary Tumor |
| TCGA-15-1444-01 | TCGA | 8.556 | GBM | Primary Tumor |
| TCGA-76-4926-01 | TCGA | 8.55 | GBM | Primary Tumor |
| TCGA-06-0156-01 | TCGA | 8.547 | GBM | Primary Tumor |
| TCGA-12-1597-01 | TCGA | 8.54 | GBM | Primary Tumor |
| TCGA-32-2634-01 | TCGA | 8.538 | GBM | Primary Tumor |
| TCGA-06-0184-01 | TCGA | 8.531 | GBM | Primary Tumor |
| TCGA-12-0821-01 | TCGA | 8.529 | GBM | Primary Tumor |
| TCGA-27-2523-01 | TCGA | 8.521 | GBM | Primary Tumor |
| TCGA-06-0646-01 | TCGA | 8.509 | GBM | Primary Tumor |
| TCGA-19-2625-01 | TCGA | 8.504 | GBM | Primary Tumor |
| TCGA-28-5216-01 | TCGA | 8.492 | GBM | Primary Tumor |
| TCGA-06-5412-01 | TCGA | 8.488 | GBM | Primary Tumor |
| TCGA-15-0742-01 | TCGA | 8.485 | GBM | Primary Tumor |
| TCGA-28-5209-01 | TCGA | 8.483 | GBM | Primary Tumor |
| TCGA-19-2619-01 | TCGA | 8.482 | GBM | Primary Tumor |
| TCGA-06-0211-01 | TCGA | 8.477 | GBM | Primary Tumor |
| TCGA-28-2514-01 | TCGA | 8.476 | GBM | Primary Tumor |
| TCGA-32-5222-01 | TCGA | 8.464 | GBM | Primary Tumor |
| TCGA-14-1034-01 | TCGA | 8.454 | GBM | Primary Tumor |
| TCGA-14-0781-01 | TCGA | 8.453 | GBM | Primary Tumor |
| TCGA-27-2528-01 | TCGA | 8.448 | GBM | Primary Tumor |
| TCGA-14-0817-01 | TCGA | 8.443 | GBM | Primary Tumor |
| TCGA-12-0618-01 | TCGA | 8.439 | GBM | Primary Tumor |
| TCGA-32-2632-01 | TCGA | 8.431 | GBM | Primary Tumor |
| TCGA-41-4097-01 | TCGA | 8.428 | GBM | Primary Tumor |
| TCGA-06-0878-01 | TCGA | 8.412 | GBM | Primary Tumor |
| TCGA-06-0644-01 | TCGA | 8.406 | GBM | Primary Tumor |
| TCGA-76-4928-01 | TCGA | 8.403 | GBM | Primary Tumor |
| TCGA-26-5139-01 | TCGA | 8.401 | GBM | Primary Tumor |
| TCGA-26-5135-01 | TCGA | 8.395 | GBM | Primary Tumor |
| TCGA-19-1787-01 | TCGA | 8.384 | GBM | Primary Tumor |
| TCGA-14-1823-01 | TCGA | 8.381 | GBM | Primary Tumor |
| TCGA-06-0649-01 | TCGA | 8.378 | GBM | Primary Tumor |
| TCGA-12-3653-01 | TCGA | 8.362 | GBM | Primary Tumor |
| TCGA-02-2483-01 | TCGA | 8.358 | GBM | Primary Tumor |
| TCGA-06-0157-01 | TCGA | 8.355 | GBM | Primary Tumor |
| TCGA-06-0130-01 | TCGA | 8.346 | GBM | Primary Tumor |
| TCGA-32-1982-01 | TCGA | 8.329 | GBM | Primary Tumor |
| TCGA-06-0138-01 | TCGA | 8.32 | GBM | Primary Tumor |
| TCGA-27-1837-01 | TCGA | 8.319 | GBM | Primary Tumor |
| TCGA-06-2559-01 | TCGA | 8.315 | GBM | Primary Tumor |
| TCGA-27-1834-01 | TCGA | 8.314 | GBM | Primary Tumor |
| TCGA-02-0055-01 | TCGA | 8.309 | GBM | Primary Tumor |
| TCGA-02-2485-01 | TCGA | 8.309 | GBM | Primary Tumor |
| TCGA-28-5215-01 | TCGA | 8.307 | GBM | Primary Tumor |
| TCGA-06-5410-01 | TCGA | 8.291 | GBM | Primary Tumor |
| TCGA-19-2620-01 | TCGA | 8.282 | GBM | Primary Tumor |
| TCGA-06-0174-01 | TCGA | 8.271 | GBM | Primary Tumor |
| TCGA-06-0187-01 | TCGA | 8.27 | GBM | Primary Tumor |
| TCGA-12-0616-01 | TCGA | 8.259 | GBM | Primary Tumor |
| TCGA-28-2509-01 | TCGA | 8.25 | GBM | Primary Tumor |
| TCGA-41-5651-01 | TCGA | 8.249 | GBM | Primary Tumor |
| TCGA-06-0158-01 | TCGA | 8.246 | GBM | Primary Tumor |
| TCGA-06-5856-01 | TCGA | 8.24 | GBM | Primary Tumor |
| TCGA-06-0238-01 | TCGA | 8.229 | GBM | Primary Tumor |
| TCGA-06-2562-01 | TCGA | 8.226 | GBM | Primary Tumor |
| TCGA-06-5414-01 | TCGA | 8.203 | GBM | Primary Tumor |
| TCGA-28-5218-01 | TCGA | 8.197 | GBM | Primary Tumor |
| TCGA-06-2563-01 | TCGA | 8.187 | GBM | Primary Tumor |
| TCGA-06-2561-01 | TCGA | 8.179 | GBM | Primary Tumor |
| TCGA-27-1835-01 | TCGA | 8.161 | GBM | Primary Tumor |
| TCGA-14-0789-01 | TCGA | 8.151 | GBM | Primary Tumor |
| TCGA-06-0686-01 | TCGA | 8.135 | GBM | Primary Tumor |
| TCGA-06-5858-01 | TCGA | 8.123 | GBM | Primary Tumor |
| TCGA-16-0846-01 | TCGA | 8.123 | GBM | Primary Tumor |
| TCGA-16-1045-01 | TCGA | 8.12 | GBM | Primary Tumor |
| TCGA-06-0645-01 | TCGA | 8.117 | GBM | Primary Tumor |
| TCGA-06-0125-01 | TCGA | 8.111 | GBM | Primary Tumor |
| TCGA-06-2570-01 | TCGA | 8.107 | GBM | Primary Tumor |
| TCGA-06-0747-01 | TCGA | 8.097 | GBM | Primary Tumor |
| TCGA-06-0141-01 | TCGA | 8.093 | GBM | Primary Tumor |
| TCGA-28-5207-01 | TCGA | 8.089 | GBM | Primary Tumor |
| TCGA-32-2615-01 | TCGA | 8.084 | GBM | Primary Tumor |
| TCGA-06-2564-01 | TCGA | 8.075 | GBM | Primary Tumor |
| TCGA-06-2557-01 | TCGA | 8.042 | GBM | Primary Tumor |
| TCGA-06-0210-01 | TCGA | 7.985 | GBM | Primary Tumor |
| TCGA-06-0139-01 | TCGA | 7.983 | GBM | Primary Tumor |
| TCGA-41-2571-01 | TCGA | 7.981 | GBM | Primary Tumor |
| TCGA-06-5417-01 | TCGA | 7.947 | GBM | Primary Tumor |
| TCGA-06-2565-01 | TCGA | 7.944 | GBM | Primary Tumor |
| TCGA-02-0047-01 | TCGA | 7.917 | GBM | Primary Tumor |
| TCGA-28-1753-01 | TCGA | 7.902 | GBM | Primary Tumor |
| TCGA-06-0129-01 | TCGA | 7.886 | GBM | Primary Tumor |
| TCGA-12-0619-01 | TCGA | 7.886 | GBM | Primary Tumor |
| TCGA-41-3915-01 | TCGA | 7.854 | GBM | Primary Tumor |
| TCGA-14-0871-01 | TCGA | 7.84 | GBM | Primary Tumor |
| TCGA-19-4065-01 | TCGA | 7.789 | GBM | Primary Tumor |
| TCGA-19-1390-01 | TCGA | 7.678 | GBM | Primary Tumor |
| TCGA-27-1830-01 | TCGA | 7.67 | GBM | Primary Tumor |
| TCGA-19-5960-01 | TCGA | 7.637 | GBM | Primary Tumor |
| TCGA-06-0190-01 | TCGA | 7.615 | GBM | Primary Tumor |
| TCGA-32-4213-01 | TCGA | 7.585 | GBM | Primary Tumor |
| TCGA-26-5134-01 | TCGA | 5.665 | GBM | Primary Tumor |

**Table S4 The normalized B166 expression and overall- survival outcomes of the patients in TCGA LGG GBM cohort**

| **Samples** | **B166** | **OS** | **OS (months)** |
| --- | --- | --- | --- |
| TCGA-FG-A6IZ-01 | 10.37 | 0 | 15.23333333 |
| TCGA-QH-A65X-01 | 10.3 | 0 | 16.06666667 |
| TCGA-FG-A6J1-01 | 10.16 | 0 | 16.66666667 |
| TCGA-HT-A74K-01 | 10.15 | 0 | 15.56666667 |
| TCGA-DB-5274-01 | 10.11 | 0 | 76.3 |
| TCGA-27-2526-01 | 10.01 | 1 | 2.9 |
| TCGA-FG-A60L-01 | 9.942 | 0 | 21.83333333 |
| TCGA-RY-A83Z-01 | 9.923 | 0 | 10.03333333 |
| TCGA-27-1831-01 | 9.896 | 1 | 16.83333333 |
| TCGA-R8-A73M-01 | 9.885 | 0 | 60.2 |
| TCGA-12-3652-01 | 9.844 | 1 | 35.4 |
| TCGA-P5-A5F6-01 | 9.84 | 0 | 1.3 |
| TCGA-06-0178-01 | 9.827 | 1 | 89.36666667 |
| TCGA-HT-A4DS-01 | 9.802 | 0 | 0.233333333 |
| TCGA-28-5213-01 | 9.795 | 0 | 31.7 |
| TCGA-FG-A6J3-01 | 9.795 | 0 | 22.56666667 |
| TCGA-VM-A8C9-01 | 9.752 | 0 | 43.8 |
| TCGA-06-0219-01 | 9.735 | 1 | 0.733333333 |
| TCGA-14-1829-01 | 9.724 | 0 | 7.266666667 |
| TCGA-VM-A8CF-01 | 9.706 | 0 | 20.3 |
| TCGA-HT-7684-01 | 9.638 | 0 | 6.133333333 |
| TCGA-76-4929-01 | 9.578 | 1 | 3.7 |
| TCGA-41-2572-01 | 9.54 | 1 | 13.53333333 |
| TCGA-DU-7290-01 | 9.516 | 1 | 10.5 |
| TCGA-27-1832-01 | 9.504 | 1 | 10 |
| TCGA-RY-A840-01 | 9.487 | 0 | 28.46666667 |
| TCGA-S9-A6TW-01 | 9.453 | 0 | 41.66666667 |
| TCGA-RY-A83Y-01 | 9.438 | 0 | 5.533333333 |
| TCGA-12-5295-01 | 9.417 | 1 | 15.13333333 |
| TCGA-VM-A8CA-01 | 9.376 | 0 | 13.7 |
| TCGA-06-0749-01 | 9.371 | 1 | 2.733333333 |
| TCGA-VM-A8CB-01 | 9.371 | 0 | 0.1 |
| TCGA-06-5411-01 | 9.346 | 1 | 8.466666667 |
| TCGA-06-5416-01 | 9.345 | 0 | 6.8 |
| TCGA-27-2524-01 | 9.328 | 1 | 7.7 |
| TCGA-12-1597-01 | 9.288 | 1 | 22.5 |
| TCGA-26-5136-01 | 9.273 | 1 | 19.23333333 |
| TCGA-06-0745-01 | 9.271 | 1 | 7.966666667 |
| TCGA-06-2569-01 | 9.266 | 0 | 0.433333333 |
| TCGA-32-4213-01 | 9.259 | 0 | 20.13333333 |
| TCGA-HT-7603-01 | 9.246 | 0 | 23.5 |
| TCGA-27-2519-01 | 9.239 | 1 | 18.33333333 |
| TCGA-HW-7490-01 | 9.239 | 0 | 46.23333333 |
| TCGA-DU-A6S8-01 | 9.228 | 0 | 22.6 |
| TCGA-DH-5141-01 | 9.222 | 0 | 32.26666667 |
| TCGA-28-5220-01 | 9.217 | 1 | 12.93333333 |
| TCGA-26-5132-01 | 9.203 | 0 | 9.533333333 |
| TCGA-QH-A6CU-01 | 9.191 | 0 | 15.03333333 |
| TCGA-14-2554-01 | 9.188 | 1 | 17.73333333 |
| TCGA-06-0168-01 | 9.182 | 1 | 19.93333333 |
| TCGA-06-0750-01 | 9.173 | 1 | 0.933333333 |
| TCGA-HT-7693-01 | 9.162 | 0 | 17.76666667 |
| TCGA-06-0132-01 | 9.154 | 1 | 25.7 |
| TCGA-06-0744-01 | 9.141 | 1 | 47.53333333 |
| TCGA-P5-A72W-01 | 9.136 | 0 | 10.56666667 |
| TCGA-HT-7681-01 | 9.098 | 0 | 45.3 |
| TCGA-06-2567-01 | 9.092 | 1 | 4.433333333 |
| TCGA-WY-A859-01 | 9.073 | 0 | 40.43333333 |
| TCGA-DH-A7UR-01 | 9.068 | 0 | 55 |
| TCGA-27-2521-01 | 9.064 | 1 | 17 |
| TCGA-DU-5874-01 | 9.054 | 0 | 15.36666667 |
| TCGA-14-0817-01 | 9.042 | 1 | 5.466666667 |
| TCGA-DU-7018-01 | 9.03 | 1 | 31.1 |
| TCGA-06-0743-01 | 9.028 | 1 | 26.76666667 |
| TCGA-28-2499-01 | 9.022 | 0 | 3.166666667 |
| TCGA-02-2486-01 | 9.009 | 1 | 20.6 |
| TCGA-76-4931-01 | 9.009 | 1 | 9.3 |
| TCGA-DU-A7TC-01 | 9.005 | 0 | 37.9 |
| TCGA-S9-A6TY-01 | 9.003 | 0 | 35.86666667 |
| TCGA-KT-A74X-01 | 9.002 | 0 | 14.6 |
| TCGA-12-5299-01 | 8.983 | 1 | 3.266666667 |
| TCGA-QH-A65Z-01 | 8.976 | 0 | 14.23333333 |
| TCGA-06-5859-01 | 8.974 | 0 | 4.633333333 |
| TCGA-06-5418-01 | 8.972 | 1 | 2.766666667 |
| TCGA-HT-A4DV-01 | 8.972 | 0 | 26.4 |
| TCGA-08-0386-01 | 8.969 | 1 | 18.26666667 |
| TCGA-QH-A6CZ-01 | 8.967 | 0 | 9.3 |
| TCGA-P5-A730-01 | 8.964 | 0 | 11.1 |
| TCGA-VW-A7QS-01 | 8.954 | 0 | 23.53333333 |
| TCGA-DB-A64L-01 | 8.951 | 0 | 24.53333333 |
| TCGA-HT-A74O-01 | 8.948 | 0 | 0.1 |
| TCGA-QH-A65S-01 | 8.947 | 0 | 2.566666667 |
| TCGA-S9-A6WH-01 | 8.944 | 0 | 39.1 |
| TCGA-06-5415-01 | 8.944 | 0 | 8.666666667 |
| TCGA-12-3650-01 | 8.938 | 1 | 11.1 |
| TCGA-14-1823-01 | 8.937 | 1 | 18.1 |
| TCGA-P5-A5F1-01 | 8.936 | 0 | 5.966666667 |
| TCGA-F6-A8O3-01 | 8.936 | 0 | 0.233333333 |
| TCGA-06-5413-01 | 8.921 | 0 | 8.933333333 |
| TCGA-76-4925-01 | 8.92 | 1 | 4.866666667 |
| TCGA-06-0184-01 | 8.916 | 1 | 70.86666667 |
| TCGA-QH-A6XC-01 | 8.913 | 0 | 16.93333333 |
| TCGA-DU-A7T6-01 | 8.91 | 1 | 18.23333333 |
| TCGA-S9-A6TV-01 | 8.906 | 0 | 19.03333333 |
| TCGA-19-2629-01 | 8.9 | 1 | 24.56666667 |
| TCGA-CS-4944-01 | 8.894 | 0 | 60.93333333 |
| TCGA-41-2571-01 | 8.893 | 1 | 0.866666667 |
| TCGA-76-4932-01 | 8.886 | 1 | 48.6 |
| TCGA-FG-5964-01 | 8.886 | 0 | 52.93333333 |
| TCGA-QH-A65V-01 | 8.885 | 0 | 12.8 |
| TCGA-14-0787-01 | 8.884 | 1 | 2.266666667 |
| TCGA-S9-A6WI-01 | 8.882 | 0 | 86.16666667 |
| TCGA-QH-A6X4-01 | 8.88 | 0 | 14.73333333 |
| TCGA-QH-A6X5-01 | 8.87 | 0 | 16.56666667 |
| TCGA-14-0790-01 | 8.858 | 1 | 13.96666667 |
| TCGA-DB-A4X9-01 | 8.858 | 0 | 47.06666667 |
| TCGA-32-2616-01 | 8.857 | 1 | 7.466666667 |
| TCGA-VM-A8C8-01 | 8.857 | 0 | 46.56666667 |
| TCGA-28-5204-01 | 8.855 | 1 | 15.13333333 |
| TCGA-QH-A65R-01 | 8.846 | 0 | 15.36666667 |
| TCGA-DU-6394-01 | 8.842 | 1 | 22.73333333 |
| TCGA-32-1980-01 | 8.842 | 1 | 1.2 |
| TCGA-26-1442-01 | 8.84 | 0 | 31.76666667 |
| TCGA-DB-A4XA-01 | 8.839 | 0 | 19.1 |
| TCGA-26-5133-01 | 8.835 | 0 | 15.06666667 |
| TCGA-S9-A6WD-01 | 8.832 | 0 | 76.3 |
| TCGA-06-0882-01 | 8.83 | 1 | 21.06666667 |
| TCGA-32-5222-01 | 8.829 | 1 | 19.5 |
| TCGA-19-2625-01 | 8.828 | 1 | 4.133333333 |
| TCGA-HT-7606-01 | 8.826 | 0 | 17.53333333 |
| TCGA-28-2513-01 | 8.826 | 0 | 7.4 |
| TCGA-HT-A617-01 | 8.82 | 0 | 16.36666667 |
| TCGA-DB-A75P-01 | 8.818 | 0 | 16.4 |
| TCGA-FG-A710-01 | 8.804 | 0 | 37.16666667 |
| TCGA-P5-A735-01 | 8.804 | 0 | 9.733333333 |
| TCGA-FG-5962-01 | 8.802 | 0 | 48.43333333 |
| TCGA-06-5412-01 | 8.796 | 1 | 4.6 |
| TCGA-06-0138-01 | 8.795 | 1 | 24.56666667 |
| TCGA-06-5408-01 | 8.789 | 1 | 11.9 |
| TCGA-HT-A74H-01 | 8.786 | 0 | 2.466666667 |
| TCGA-DB-A75L-01 | 8.779 | 0 | 11.4 |
| TCGA-P5-A77X-01 | 8.779 | 0 | 3.5 |
| TCGA-DU-A76R-01 | 8.777 | 1 | 21.6 |
| TCGA-DU-A76O-01 | 8.776 | 0 | 17.4 |
| TCGA-P5-A5EV-01 | 8.771 | 0 | 0.233333333 |
| TCGA-HW-A5KJ-01 | 8.767 | 1 | 32.06666667 |
| TCGA-S9-A6U5-01 | 8.765 | 0 | 33.06666667 |
| TCGA-HT-7476-01 | 8.762 | 0 | 6.633333333 |
| TCGA-HT-7605-01 | 8.762 | 0 | 4.633333333 |
| TCGA-S9-A6TX-01 | 8.76 | 0 | 41.5 |
| TCGA-R8-A6MO-01 | 8.76 | 0 | 33.1 |
| TCGA-E1-A7YY-01 | 8.759 | 1 | 148.1666667 |
| TCGA-QH-A6CV-01 | 8.759 | 0 | 14.73333333 |
| TCGA-HT-7608-01 | 8.756 | 0 | 22.36666667 |
| TCGA-DB-A64V-01 | 8.755 | 0 | 29.63333333 |
| TCGA-06-0649-01 | 8.754 | 1 | 2.133333333 |
| TCGA-DB-A64P-01 | 8.754 | 0 | 30.53333333 |
| TCGA-TM-A84R-01 | 8.752 | 0 | 18.86666667 |
| TCGA-S9-A7QY-01 | 8.751 | 0 | 28.76666667 |
| TCGA-S9-A6UB-01 | 8.75 | 0 | 125.3666667 |
| TCGA-02-2483-01 | 8.75 | 0 | 15.53333333 |
| TCGA-06-1804-01 | 8.748 | 1 | 13.8 |
| TCGA-HT-8018-01 | 8.743 | 1 | 38.4 |
| TCGA-28-5208-01 | 8.743 | 1 | 18.13333333 |
| TCGA-06-0238-01 | 8.743 | 1 | 13.5 |
| TCGA-DU-6393-01 | 8.742 | 1 | 52.83333333 |
| TCGA-P5-A5F4-01 | 8.742 | 0 | 7.7 |
| TCGA-DU-A76K-01 | 8.739 | 1 | 11.56666667 |
| TCGA-FG-8181-01 | 8.738 | 0 | 28.73333333 |
| TCGA-FG-A4MX-01 | 8.736 | 0 | 18.96666667 |
| TCGA-E1-5318-01 | 8.734 | 1 | 79.3 |
| TCGA-15-0742-01 | 8.734 | 1 | 13.96666667 |
| TCGA-HT-7692-01 | 8.733 | 0 | 3 |
| TCGA-DU-A5TU-01 | 8.731 | 0 | 26.53333333 |
| TCGA-DB-A75O-01 | 8.729 | 0 | 31.16666667 |
| TCGA-DU-6400-01 | 8.728 | 1 | 1.233333333 |
| TCGA-06-0156-01 | 8.727 | 1 | 5.933333333 |
| TCGA-HT-A61C-01 | 8.724 | 1 | 17.9 |
| TCGA-DH-5144-01 | 8.724 | 0 | 24.93333333 |
| TCGA-DB-A64X-01 | 8.724 | 0 | 18.76666667 |
| TCGA-FG-7636-01 | 8.718 | 0 | 18.13333333 |
| TCGA-P5-A5EX-01 | 8.718 | 0 | 8.1 |
| TCGA-19-2624-01 | 8.714 | 1 | 0.166666667 |
| TCGA-P5-A731-01 | 8.714 | 0 | 9.133333333 |
| TCGA-S9-A6TS-01 | 8.713 | 1 | 63.03333333 |
| TCGA-28-1747-01 | 8.712 | 1 | 2.566666667 |
| TCGA-E1-A7Z2-01 | 8.71 | 1 | 13.26666667 |
| TCGA-HT-7471-01 | 8.708 | 0 | 0.133333333 |
| TCGA-32-1982-01 | 8.707 | 1 | 4.733333333 |
| TCGA-06-0141-01 | 8.705 | 1 | 10.43333333 |
| TCGA-F6-A8O4-01 | 8.705 | 0 | 0.2 |
| TCGA-TQ-A7RU-01 | 8.699 | 0 | 34.4 |
| TCGA-HT-8019-01 | 8.695 | 0 | 33.46666667 |
| TCGA-DU-A6S3-01 | 8.694 | 0 | 21.86666667 |
| TCGA-S9-A7IQ-01 | 8.691 | 0 | 36.63333333 |
| TCGA-DU-5872-01 | 8.691 | 0 | 17.73333333 |
| TCGA-R8-A6ML-01 | 8.69 | 0 | 95.33333333 |
| TCGA-TQ-A7RF-01 | 8.69 | 0 | 21.66666667 |
| TCGA-S9-A6WP-01 | 8.69 | 0 | 18.9 |
| TCGA-HT-7690-01 | 8.689 | 0 | 0.1 |
| TCGA-S9-A6U6-01 | 8.687 | 0 | 35.63333333 |
| TCGA-P5-A5ET-01 | 8.683 | 0 | 0 |
| TCGA-FG-7638-01 | 8.678 | 0 | 22.86666667 |
| TCGA-TM-A84S-01 | 8.678 | 0 | 15.13333333 |
| TCGA-27-2528-01 | 8.677 | 1 | 16 |
| TCGA-FG-8185-01 | 8.672 | 0 | 14.43333333 |
| TCGA-06-5410-01 | 8.67 | 1 | 3.6 |
| TCGA-WH-A86K-01 | 8.669 | 0 | 13.5 |
| TCGA-76-4927-01 | 8.667 | 1 | 17.83333333 |
| TCGA-02-0055-01 | 8.667 | 1 | 2.533333333 |
| TCGA-14-0781-01 | 8.666 | 1 | 0.966666667 |
| TCGA-DU-A6S6-01 | 8.665 | 0 | 96.43333333 |
| TCGA-TQ-A7RN-01 | 8.664 | 0 | 34.2 |
| TCGA-HT-7694-01 | 8.661 | 0 | 7 |
| TCGA-06-2558-01 | 8.66 | 1 | 12.66666667 |
| TCGA-P5-A736-01 | 8.66 | 0 | 7.666666667 |
| TCGA-32-2632-01 | 8.658 | 1 | 8.966666667 |
| TCGA-E1-5319-01 | 8.657 | 1 | 96.9 |
| TCGA-28-5216-01 | 8.657 | 0 | 13.83333333 |
| TCGA-FG-A4MT-01 | 8.656 | 0 | 38.8 |
| TCGA-14-1825-01 | 8.655 | 1 | 7.733333333 |
| TCGA-QH-A6X8-01 | 8.651 | 0 | 16.96666667 |
| TCGA-TQ-A7RS-01 | 8.649 | 0 | 0 |
| TCGA-TM-A84G-01 | 8.647 | 0 | 41.2 |
| TCGA-S9-A7IY-01 | 8.647 | 0 | 23.83333333 |
| TCGA-HT-7616-01 | 8.646 | 1 | 0.233333333 |
| TCGA-HT-7691-01 | 8.644 | 0 | 0.1 |
| TCGA-DU-7008-01 | 8.639 | 0 | 158.4 |
| TCGA-32-1970-01 | 8.638 | 1 | 15.6 |
| TCGA-12-0821-01 | 8.638 | 1 | 10.76666667 |
| TCGA-HT-A5R7-01 | 8.636 | 0 | 20.5 |
| TCGA-RY-A847-01 | 8.635 | 0 | 31.1 |
| TCGA-EZ-7264-01 | 8.633 | 0 | 40.03333333 |
| TCGA-S9-A6WE-01 | 8.632 | 0 | 137.1 |
| TCGA-FG-7643-01 | 8.632 | 0 | 20.36666667 |
| TCGA-DB-A75M-01 | 8.632 | 0 | 18.13333333 |
| TCGA-P5-A733-01 | 8.631 | 0 | 13.8 |
| TCGA-28-2510-01 | 8.629 |  | 0 |
| TCGA-FG-A711-01 | 8.626 | 1 | 49.36666667 |
| TCGA-FG-8186-01 | 8.626 | 0 | 16.23333333 |
| TCGA-76-4926-01 | 8.624 | 1 | 4.6 |
| TCGA-DU-A5TW-01 | 8.623 | 0 | 25.33333333 |
| TCGA-P5-A781-01 | 8.619 | 0 | 4.466666667 |
| TCGA-S9-A7QW-01 | 8.618 | 0 | 15.26666667 |
| TCGA-12-0616-01 | 8.614 | 1 | 14.93333333 |
| TCGA-QH-A6CX-01 | 8.61 | 1 | 12.4 |
| TCGA-32-2634-01 | 8.609 | 0 | 23.1 |
| TCGA-HT-7676-01 | 8.608 | 0 | 0.166666667 |
| TCGA-CS-5394-01 | 8.607 | 0 | 0.266666667 |
| TCGA-FG-7637-01 | 8.606 | 0 | 40.63333333 |
| TCGA-CS-6186-01 | 8.603 | 1 | 17.93333333 |
| TCGA-DB-A64W-01 | 8.602 | 1 | 14.6 |
| TCGA-CS-6670-01 | 8.594 | 0 | 47.53333333 |
| TCGA-QH-A6CY-01 | 8.594 | 0 | 13.6 |
| TCGA-FG-6692-01 | 8.593 | 1 | 18.7 |
| TCGA-76-4928-01 | 8.592 | 1 | 3.133333333 |
| TCGA-DH-A66F-01 | 8.592 | 0 | 17.43333333 |
| TCGA-06-0130-01 | 8.59 | 1 | 13.13333333 |
| TCGA-DB-5279-01 | 8.589 | 0 | 45.13333333 |
| TCGA-19-1787-01 | 8.588 | 1 | 12.83333333 |
| TCGA-DB-5270-01 | 8.588 | 0 | 124.4333333 |
| TCGA-DU-A5TR-01 | 8.587 | 0 | 30.26666667 |
| TCGA-DB-A64O-01 | 8.585 | 1 | 25.83333333 |
| TCGA-VM-A8CD-01 | 8.584 | 1 | 8 |
| TCGA-TM-A7C5-01 | 8.584 | 0 | 50 |
| TCGA-S9-A89Z-01 | 8.584 | 0 | 20.76666667 |
| TCGA-HT-7607-01 | 8.581 | 1 | 3.2 |
| TCGA-26-5135-01 | 8.579 | 1 | 9 |
| TCGA-HW-7487-01 | 8.578 | 0 | 46.06666667 |
| TCGA-HW-7489-01 | 8.577 | 1 | 42.06666667 |
| TCGA-HT-8113-01 | 8.571 | 0 | 30 |
| TCGA-R8-A6MK-01 | 8.562 | 0 | 90.06666667 |
| TCGA-FG-A70Z-01 | 8.56 | 0 | 10.93333333 |
| TCGA-WY-A85A-01 | 8.558 | 0 | 44 |
| TCGA-15-1444-01 | 8.557 | 1 | 51.23333333 |
| TCGA-32-2638-01 | 8.556 | 1 | 25.53333333 |
| TCGA-DU-7292-01 | 8.555 | 1 | 8.066666667 |
| TCGA-DH-5142-01 | 8.553 | 0 | 64.76666667 |
| TCGA-P5-A5EY-01 | 8.55 | 0 | 2.4 |
| TCGA-DU-6395-01 | 8.548 | 1 | 49.7 |
| TCGA-DU-5849-01 | 8.547 | 0 | 14.76666667 |
| TCGA-06-0646-01 | 8.544 | 1 | 5.833333333 |
| TCGA-DU-5847-01 | 8.542 | 0 | 18.26666667 |
| TCGA-W9-A837-01 | 8.541 | 0 | 51.76666667 |
| TCGA-RY-A845-01 | 8.54 | 0 | 19.86666667 |
| TCGA-P5-A5F2-01 | 8.54 | 0 | 8.066666667 |
| TCGA-06-0644-01 | 8.537 | 1 | 12.8 |
| TCGA-06-0211-01 | 8.537 | 1 | 12 |
| TCGA-HT-7688-01 | 8.537 | 0 | 32.13333333 |
| TCGA-RY-A83X-01 | 8.536 | 0 | 31.3 |
| TCGA-TM-A7C4-01 | 8.533 | 0 | 49 |
| TCGA-27-2523-01 | 8.532 | 1 | 16.3 |
| TCGA-CS-5397-01 | 8.531 | 1 | 6.466666667 |
| TCGA-28-2514-01 | 8.531 | 0 | 5.333333333 |
| TCGA-19-2620-01 | 8.527 | 1 | 4.933333333 |
| TCGA-FG-6688-01 | 8.526 | 0 | 19.03333333 |
| TCGA-27-1835-01 | 8.524 | 1 | 21.6 |
| TCGA-DU-7309-01 | 8.523 | 0 | 2.8 |
| TCGA-DU-6397-01 | 8.522 | 1 | 46.7 |
| TCGA-27-1837-01 | 8.522 | 1 | 14.23333333 |
| TCGA-FG-A713-01 | 8.522 | 0 | 20.73333333 |
| TCGA-HT-A615-01 | 8.521 | 0 | 17.16666667 |
| TCGA-26-5139-01 | 8.517 | 0 | 1.6 |
| TCGA-HT-7481-01 | 8.514 | 0 | 97.26666667 |
| TCGA-28-2509-01 | 8.513 | 0 | 4.833333333 |
| TCGA-E1-A7Z3-01 | 8.512 | 1 | 74.5 |
| TCGA-DU-5870-01 | 8.512 | 0 | 184.8666667 |
| TCGA-DH-5143-01 | 8.509 | 0 | 46.7 |
| TCGA-41-4097-01 | 8.508 | 1 | 0.2 |
| TCGA-P5-A737-01 | 8.508 | 0 | 12.4 |
| TCGA-DU-A5TS-01 | 8.506 | 0 | 32.13333333 |
| TCGA-FG-8189-01 | 8.505 | 0 | 22.83333333 |
| TCGA-HT-8105-01 | 8.505 | 0 | 6.333333333 |
| TCGA-DU-8164-01 | 8.504 | 0 | 21.7 |
| TCGA-P5-A72Z-01 | 8.504 | 0 | 11.8 |
| TCGA-HT-8010-01 | 8.504 | 0 | 1.666666667 |
| TCGA-DB-A75K-01 | 8.503 | 0 | 12.26666667 |
| TCGA-P5-A5F0-01 | 8.5 | 0 | 9.566666667 |
| TCGA-HT-A61A-01 | 8.5 | 0 | 6.466666667 |
| TCGA-DB-5278-01 | 8.497 | 0 | 92.4 |
| TCGA-06-0878-01 | 8.497 | 0 | 7.266666667 |
| TCGA-06-5417-01 | 8.495 | 0 | 5.166666667 |
| TCGA-DU-7304-01 | 8.494 | 1 | 23.63333333 |
| TCGA-DH-A66B-01 | 8.494 | 0 | 42.63333333 |
| TCGA-TQ-A8XE-01 | 8.493 | 1 | 31.8 |
| TCGA-S9-A7R2-01 | 8.493 | 1 | 10.53333333 |
| TCGA-E1-A7YU-01 | 8.493 | 1 | 0.766666667 |
| TCGA-HW-7491-01 | 8.493 | 0 | 69.26666667 |
| TCGA-DB-5275-01 | 8.493 | 0 | 48.6 |
| TCGA-HT-A5R9-01 | 8.488 | 0 | 0.066666667 |
| TCGA-CS-6667-01 | 8.48 | 0 | 48.96666667 |
| TCGA-26-5134-01 | 8.477 | 0 | 5.566666667 |
| TCGA-CS-5396-01 | 8.473 | 0 | 54.36666667 |
| TCGA-S9-A6WQ-01 | 8.473 | 0 | 14.26666667 |
| TCGA-HT-A616-01 | 8.473 | 0 | 1.833333333 |
| TCGA-DU-7007-01 | 8.472 | 1 | 63.83333333 |
| TCGA-S9-A7IZ-01 | 8.472 | 0 | 20.26666667 |
| TCGA-HT-7475-01 | 8.47 | 0 | 17.66666667 |
| TCGA-DB-A64S-01 | 8.469 | 0 | 27.83333333 |
| TCGA-IK-8125-01 | 8.466 | 0 | 43.36666667 |
| TCGA-DU-7294-01 | 8.465 | 0 | 95.63333333 |
| TCGA-HT-7474-01 | 8.464 | 0 | 3.8 |
| TCGA-FG-A87Q-01 | 8.463 | 0 | 5.8 |
| TCGA-DB-A4XG-01 | 8.461 | 0 | 73.96666667 |
| TCGA-HT-8015-01 | 8.461 | 0 | 0.1 |
| TCGA-14-1034-01 | 8.46 | 1 | 16.16666667 |
| TCGA-S9-A7IX-01 | 8.459 | 1 | 27.3 |
| TCGA-VV-A829-01 | 8.458 | 0 | 37.56666667 |
| TCGA-19-2619-01 | 8.457 | 0 | 9.8 |
| TCGA-S9-A7J0-01 | 8.456 | 0 | 20.3 |
| TCGA-P5-A77W-01 | 8.452 | 0 | 19.46666667 |
| TCGA-28-5209-01 | 8.452 | 0 | 14.73333333 |
| TCGA-06-0157-01 | 8.451 | 1 | 3.233333333 |
| TCGA-DU-7009-01 | 8.446 | 1 | 156.5 |
| TCGA-DU-A5TT-01 | 8.446 | 0 | 24.76666667 |
| TCGA-CS-6666-01 | 8.443 | 0 | 47.6 |
| TCGA-S9-A7R1-01 | 8.442 | 1 | 172.2 |
| TCGA-HW-7486-01 | 8.438 | 0 | 57.36666667 |
| TCGA-WY-A858-01 | 8.438 | 0 | 44.56666667 |
| TCGA-HT-7695-01 | 8.437 | 0 | 14.73333333 |
| TCGA-DU-A7TG-01 | 8.436 | 1 | 45.03333333 |
| TCGA-FG-8187-01 | 8.436 | 0 | 20.36666667 |
| TCGA-DH-A669-01 | 8.435 | 1 | 30.63333333 |
| TCGA-DU-7306-01 | 8.435 | 0 | 42.56666667 |
| TCGA-DH-A7US-01 | 8.435 | 0 | 23.53333333 |
| TCGA-HT-7483-01 | 8.434 | 0 | 175.1666667 |
| TCGA-S9-A7R3-01 | 8.434 | 0 | 100.4333333 |
| TCGA-DB-A4XE-01 | 8.431 | 0 | 37.96666667 |
| TCGA-DU-7302-01 | 8.429 | 0 | 61.13333333 |
| TCGA-QH-A6XA-01 | 8.427 | 0 | 16.73333333 |
| TCGA-DB-A64Q-01 | 8.426 | 0 | 6.066666667 |
| TCGA-FG-A4MW-01 | 8.425 | 1 | 18.63333333 |
| TCGA-S9-A6U0-01 | 8.424 | 1 | 24.73333333 |
| TCGA-HT-7856-01 | 8.424 | 0 | 39.63333333 |
| TCGA-FG-6689-01 | 8.422 | 0 | 15.13333333 |
| TCGA-HT-8558-01 | 8.421 | 0 | 13.93333333 |
| TCGA-TM-A84O-01 | 8.42 | 1 | 33.7 |
| TCGA-12-0618-01 | 8.42 | 1 | 13.16666667 |
| TCGA-06-0187-01 | 8.417 | 1 | 27.6 |
| TCGA-HT-7874-01 | 8.416 | 0 | 37.66666667 |
| TCGA-P5-A72X-01 | 8.415 | 0 | 13.43333333 |
| TCGA-DU-6399-01 | 8.411 | 1 | 66.66666667 |
| TCGA-02-2485-01 | 8.411 | 0 | 15.66666667 |
| TCGA-E1-5305-01 | 8.41 | 1 | 81.1 |
| TCGA-CS-4942-01 | 8.406 | 1 | 44.5 |
| TCGA-WY-A85D-01 | 8.406 | 0 | 38.23333333 |
| TCGA-HT-7880-01 | 8.406 | 0 | 5.4 |
| TCGA-S9-A7QX-01 | 8.403 | 0 | 18.8 |
| TCGA-DU-7299-01 | 8.397 | 1 | 44.63333333 |
| TCGA-HT-8104-01 | 8.396 | 0 | 12.4 |
| TCGA-06-2563-01 | 8.394 | 0 | 31.06666667 |
| TCGA-S9-A6WL-01 | 8.391 | 0 | 31.53333333 |
| TCGA-CS-4943-01 | 8.39 | 1 | 36.86666667 |
| TCGA-HT-7687-01 | 8.39 | 0 | 0.1 |
| TCGA-IK-7675-01 | 8.389 | 1 | 19.26666667 |
| TCGA-12-3653-01 | 8.387 | 1 | 14.73333333 |
| TCGA-41-3915-01 | 8.384 | 1 | 12 |
| TCGA-14-0871-01 | 8.383 | 1 | 29.33333333 |
| TCGA-HT-7602-01 | 8.381 | 0 | 30.26666667 |
| TCGA-P5-A72U-01 | 8.379 | 0 | 0.233333333 |
| TCGA-E1-5302-01 | 8.373 | 1 | 50.83333333 |
| TCGA-DU-5871-01 | 8.371 | 0 | 19.2 |
| TCGA-DU-6402-01 | 8.368 | 1 | 7.133333333 |
| TCGA-DB-A64R-01 | 8.365 | 0 | 31.83333333 |
| TCGA-DU-7300-01 | 8.364 | 1 | 62.86666667 |
| TCGA-HT-7677-01 | 8.362 | 0 | 16.46666667 |
| TCGA-E1-A7YK-01 | 8.36 | 1 | 12.6 |
| TCGA-CS-6669-01 | 8.358 | 0 | 49.8 |
| TCGA-TQ-A7RG-01 | 8.358 | 0 | 19.96666667 |
| TCGA-HT-A5R5-01 | 8.357 | 0 | 0.133333333 |
| TCGA-27-1834-01 | 8.356 | 1 | 41.1 |
| TCGA-06-0645-01 | 8.355 | 1 | 5.833333333 |
| TCGA-HT-8012-01 | 8.355 | 0 | 9.533333333 |
| TCGA-DU-6392-01 | 8.354 | 0 | 214.1 |
| TCGA-S9-A7QZ-01 | 8.354 | 0 | 27.53333333 |
| TCGA-HT-8107-01 | 8.351 | 0 | 0.466666667 |
| TCGA-DU-8161-01 | 8.35 | 1 | 24.06666667 |
| TCGA-DU-7019-01 | 8.35 | 0 | 26.66666667 |
| TCGA-06-2561-01 | 8.348 | 1 | 17.9 |
| TCGA-DU-A7T8-01 | 8.346 | 1 | 140.9666667 |
| TCGA-S9-A7IS-01 | 8.345 | 1 | 8.033333333 |
| TCGA-CS-4938-01 | 8.344 | 0 | 119.1333333 |
| TCGA-DU-8167-01 | 8.344 | 0 | 15.7 |
| TCGA-TQ-A7RK-01 | 8.342 | 0 | 44.7 |
| TCGA-HT-7881-01 | 8.341 | 0 | 35.96666667 |
| TCGA-DU-6410-01 | 8.341 | 0 | 8.066666667 |
| TCGA-P5-A5EU-01 | 8.341 | 0 | 0 |
| TCGA-TM-A84Q-01 | 8.339 | 0 | 25.73333333 |
| TCGA-HT-7604-01 | 8.338 | 0 | 124.1666667 |
| TCGA-HT-8109-01 | 8.338 | 0 | 5.633333333 |
| TCGA-TM-A84L-01 | 8.337 | 1 | 41.4 |
| TCGA-16-0846-01 | 8.332 | 1 | 3.966666667 |
| TCGA-HT-8011-01 | 8.326 | 0 | 16.46666667 |
| TCGA-06-5856-01 | 8.321 | 1 | 3.8 |
| TCGA-QH-A86X-01 | 8.32 | 0 | 11.23333333 |
| TCGA-S9-A7J2-01 | 8.318 | 0 | 2.066666667 |
| TCGA-QH-A870-01 | 8.317 | 0 | 13.16666667 |
| TCGA-HT-7875-01 | 8.317 | 0 | 0.333333333 |
| TCGA-DU-6408-01 | 8.315 | 1 | 115.6666667 |
| TCGA-06-0686-01 | 8.315 | 1 | 14.4 |
| TCGA-FG-8188-01 | 8.315 | 0 | 15.16666667 |
| TCGA-06-5414-01 | 8.307 | 0 | 9.1 |
| TCGA-E1-A7YM-01 | 8.305 | 1 | 21.6 |
| TCGA-DU-8162-01 | 8.303 | 1 | 14.8 |
| TCGA-TM-A84M-01 | 8.303 | 0 | 25.13333333 |
| TCGA-HT-7467-01 | 8.302 | 0 | 0.1 |
| TCGA-28-5218-01 | 8.3 | 1 | 5.233333333 |
| TCGA-TM-A84F-01 | 8.296 | 0 | 59.86666667 |
| TCGA-VM-A8CE-01 | 8.295 | 0 | 39.7 |
| TCGA-E1-5304-01 | 8.292 | 1 | 41.7 |
| TCGA-06-2559-01 | 8.292 | 1 | 5 |
| TCGA-DU-A7TA-01 | 8.291 | 0 | 85.5 |
| TCGA-DB-5273-01 | 8.29 | 0 | 83.1 |
| TCGA-E1-5307-01 | 8.289 | 1 | 58.73333333 |
| TCGA-HW-7495-01 | 8.289 | 0 | 35.93333333 |
| TCGA-E1-A7YN-01 | 8.285 | 1 | 24.23333333 |
| TCGA-DU-A6S7-01 | 8.285 | 0 | 21.26666667 |
| TCGA-06-2562-01 | 8.284 | 1 | 12.73333333 |
| TCGA-DU-7014-01 | 8.283 | 1 | 119.0333333 |
| TCGA-06-0210-01 | 8.277 | 1 | 7.5 |
| TCGA-DU-7301-01 | 8.275 | 1 | 26.26666667 |
| TCGA-06-0174-01 | 8.274 | 1 | 3.266666667 |
| TCGA-HT-7689-01 | 8.272 | 0 | 15.16666667 |
| TCGA-DU-5852-01 | 8.271 | 1 | 6.833333333 |
| TCGA-HT-A74J-01 | 8.269 | 0 | 10.66666667 |
| TCGA-TM-A84B-01 | 8.268 | 1 | 25.26666667 |
| TCGA-HT-8564-01 | 8.266 | 0 | 15.93333333 |
| TCGA-DU-6401-01 | 8.254 | 1 | 88.66666667 |
| TCGA-FG-A60J-01 | 8.253 | 0 | 14.96666667 |
| TCGA-DH-A7UV-01 | 8.252 | 0 | 18.86666667 |
| TCGA-HT-7877-01 | 8.252 | 0 | 0.133333333 |
| TCGA-32-2615-01 | 8.251 | 1 | 16.16666667 |
| TCGA-WY-A85B-01 | 8.249 | 0 | 46.43333333 |
| TCGA-DU-8168-01 | 8.249 | 0 | 14.36666667 |
| TCGA-06-2570-01 | 8.248 | 0 | 31.93333333 |
| TCGA-HW-7493-01 | 8.247 | 0 | 70.23333333 |
| TCGA-HT-7485-01 | 8.247 | 0 | 4.066666667 |
| TCGA-DU-7011-01 | 8.246 | 1 | 106.6666667 |
| TCGA-HT-7478-01 | 8.245 | 0 | 6.466666667 |
| TCGA-E1-A7YJ-01 | 8.244 | 1 | 19.73333333 |
| TCGA-14-0789-01 | 8.24 | 1 | 11.4 |
| TCGA-41-5651-01 | 8.238 | 1 | 15.33333333 |
| TCGA-HT-7479-01 | 8.238 | 0 | 40.9 |
| TCGA-06-2557-01 | 8.236 | 1 | 1.1 |
| TCGA-E1-A7Z4-01 | 8.235 | 1 | 147.0666667 |
| TCGA-RY-A843-01 | 8.235 | 0 | 2.1 |
| TCGA-HW-8319-01 | 8.232 | 1 | 40.3 |
| TCGA-HT-7468-01 | 8.23 | 0 | 6.766666667 |
| TCGA-VV-A86M-01 | 8.229 | 0 | 16.23333333 |
| TCGA-06-0158-01 | 8.227 | 1 | 10.96666667 |
| TCGA-FG-A70Y-01 | 8.222 | 0 | 28.93333333 |
| TCGA-S9-A7J3-01 | 8.22 | 0 | 20.96666667 |
| TCGA-QH-A6X9-01 | 8.22 | 0 | 1.933333333 |
| TCGA-HT-7477-01 | 8.217 | 0 | 24.6 |
| TCGA-TQ-A7RV-01 | 8.216 | 0 | 62.26666667 |
| TCGA-HT-7480-01 | 8.215 | 0 | 76.23333333 |
| TCGA-HT-A614-01 | 8.214 | 0 | 2.733333333 |
| TCGA-16-1045-01 | 8.212 | 1 | 29.43333333 |
| TCGA-FG-A4MY-01 | 8.212 | 0 | 24.03333333 |
| TCGA-S9-A6TU-01 | 8.211 | 0 | 88.33333333 |
| TCGA-S9-A6U2-01 | 8.211 | 0 | 30.26666667 |
| TCGA-E1-5311-01 | 8.21 | 1 | 136.1333333 |
| TCGA-HT-A5RA-01 | 8.21 | 0 | 27.73333333 |
| TCGA-HT-7620-01 | 8.209 | 0 | 14.46666667 |
| TCGA-HT-8013-01 | 8.207 | 1 | 64.43333333 |
| TCGA-DU-7298-01 | 8.207 | 1 | 19.2 |
| TCGA-TQ-A7RI-01 | 8.204 | 0 | 2.066666667 |
| TCGA-E1-5322-01 | 8.203 | 1 | 132.6 |
| TCGA-VM-A8CH-01 | 8.202 | 0 | 23.8 |
| TCGA-FG-5965-01 | 8.201 | 1 | 37.33333333 |
| TCGA-S9-A7J1-01 | 8.199 | 0 | 8.3 |
| TCGA-HT-8114-01 | 8.198 | 0 | 34.66666667 |
| TCGA-WY-A85E-01 | 8.198 | 0 | 21.1 |
| TCGA-S9-A6U1-01 | 8.197 | 0 | 26.16666667 |
| TCGA-DU-5855-01 | 8.195 | 0 | 6.9 |
| TCGA-DU-A6S2-01 | 8.192 | 0 | 25.9 |
| TCGA-19-4065-01 | 8.192 | 0 | 7.133333333 |
| TCGA-DH-A7UT-01 | 8.191 | 1 | 17.7 |
| TCGA-TM-A7CA-01 | 8.191 | 0 | 35.26666667 |
| TCGA-28-5215-01 | 8.189 | 1 | 11.16666667 |
| TCGA-06-0125-02 | 8.187 | 1 | 48.26666667 |
| TCGA-TQ-A7RO-01 | 8.186 | 0 | 31.63333333 |
| TCGA-HT-8111-01 | 8.185 | 0 | 0.233333333 |
| TCGA-DB-A64U-01 | 8.178 | 0 | 28.2 |
| TCGA-WY-A85C-01 | 8.175 | 0 | 47.53333333 |
| TCGA-TQ-A7RJ-01 | 8.175 | 0 | 40.96666667 |
| TCGA-FG-A60K-01 | 8.175 | 0 | 18.06666667 |
| TCGA-TQ-A7RP-01 | 8.174 | 0 | 30.46666667 |
| TCGA-FG-5963-01 | 8.173 | 1 | 25.83333333 |
| TCGA-HT-7686-01 | 8.172 | 0 | 43.33333333 |
| TCGA-HT-7680-01 | 8.171 | 0 | 0.766666667 |
| TCGA-HT-A618-01 | 8.17 | 0 | 17.06666667 |
| TCGA-DU-6407-01 | 8.169 | 1 | 95.83333333 |
| TCGA-FG-7641-01 | 8.168 | 0 | 20.9 |
| TCGA-TQ-A7RQ-01 | 8.167 | 0 | 26.5 |
| TCGA-HT-A74L-01 | 8.166 | 0 | 11.2 |
| TCGA-VW-A8FI-01 | 8.162 | 1 | 8.166666667 |
| TCGA-HT-7610-01 | 8.161 | 0 | 56.86666667 |
| TCGA-HT-7470-01 | 8.16 | 1 | 40.66666667 |
| TCGA-E1-A7YD-01 | 8.155 | 1 | 14.5 |
| TCGA-06-0747-01 | 8.155 | 1 | 2.733333333 |
| TCGA-S9-A6U9-01 | 8.155 | 0 | 97.26666667 |
| TCGA-CS-6290-01 | 8.152 | 1 | 37.9 |
| TCGA-E1-A7YV-01 | 8.15 | 1 | 32.9 |
| TCGA-E1-A7YQ-01 | 8.149 | 1 | 52.6 |
| TCGA-HT-7611-01 | 8.148 | 0 | 58.4 |
| TCGA-FG-6691-01 | 8.147 | 0 | 41.9 |
| TCGA-S9-A7R7-01 | 8.145 | 0 | 100 |
| TCGA-CS-6188-01 | 8.142 | 1 | 27.13333333 |
| TCGA-DU-6405-01 | 8.142 | 1 | 20.16666667 |
| TCGA-HW-8322-01 | 8.142 | 0 | 25.26666667 |
| TCGA-FG-7634-01 | 8.139 | 0 | 15.56666667 |
| TCGA-28-5207-01 | 8.137 | 1 | 11.43333333 |
| TCGA-R8-A6YH-01 | 8.131 |  | 0 |
| TCGA-TM-A84T-01 | 8.125 | 0 | 24.13333333 |
| TCGA-DB-5280-01 | 8.122 | 0 | 37.06666667 |
| TCGA-CS-5390-01 | 8.12 | 0 | 0 |
| TCGA-S9-A6U8-01 | 8.119 | 1 | 99.6 |
| TCGA-E1-A7YO-01 | 8.119 | 1 | 76.06666667 |
| TCGA-06-0125-01 | 8.118 | 1 | 48.26666667 |
| TCGA-06-5858-01 | 8.118 | 0 | 6.233333333 |
| TCGA-E1-A7Z6-01 | 8.117 | 1 | 32.8 |
| TCGA-DU-8158-01 | 8.112 | 1 | 5.166666667 |
| TCGA-DU-A5TY-01 | 8.111 | 1 | 34.43333333 |
| TCGA-E1-A7YL-01 | 8.11 | 1 | 16.4 |
| TCGA-06-0152-02 | 8.11 | 1 | 12.5 |
| TCGA-CS-4941-01 | 8.109 | 1 | 7.8 |
| TCGA-DU-A7TJ-01 | 8.107 | 0 | 0.566666667 |
| TCGA-DB-5276-01 | 8.106 | 0 | 73.93333333 |
| TCGA-TM-A7C3-01 | 8.104 | 1 | 55.53333333 |
| TCGA-HT-A619-01 | 8.104 | 0 | 21.7 |
| TCGA-DU-5851-01 | 8.102 | 0 | 17.7 |
| TCGA-DU-A5TP-01 | 8.101 | 0 | 33.73333333 |
| TCGA-HT-7482-01 | 8.098 | 0 | 108.4333333 |
| TCGA-HW-8321-01 | 8.094 | 0 | 43.13333333 |
| TCGA-TM-A7CF-01 | 8.092 | 0 | 66.3 |
| TCGA-HT-7601-01 | 8.088 | 0 | 5.1 |
| TCGA-DB-A4XF-01 | 8.083 | 0 | 34.03333333 |
| TCGA-DB-A4XH-01 | 8.08 | 0 | 32.06666667 |
| TCGA-QH-A6X3-01 | 8.08 | 0 | 10.43333333 |
| TCGA-HT-7879-01 | 8.079 | 0 | 3.733333333 |
| TCGA-E1-A7YI-01 | 8.069 | 1 | 3.7 |
| TCGA-DU-5853-01 | 8.069 | 0 | 13.56666667 |
| TCGA-DB-A4XC-01 | 8.067 | 0 | 47.36666667 |
| TCGA-HT-7855-01 | 8.064 | 0 | 19.5 |
| TCGA-28-1753-01 | 8.06 | 0 | 1.233333333 |
| TCGA-TQ-A7RH-01 | 8.059 | 0 | 16.2 |
| TCGA-TQ-A7RR-01 | 8.058 | 0 | 26.23333333 |
| TCGA-HT-7609-01 | 8.057 | 0 | 46.63333333 |
| TCGA-CS-6668-01 | 8.053 | 0 | 50.63333333 |
| TCGA-06-2564-01 | 8.052 | 0 | 6.033333333 |
| TCGA-TM-A84C-01 | 8.047 | 1 | 16.4 |
| TCGA-S9-A6WN-01 | 8.042 | 0 | 26.83333333 |
| TCGA-DU-8165-01 | 8.042 | 0 | 19.4 |
| TCGA-HT-8110-01 | 8.039 | 0 | 13.96666667 |
| TCGA-CS-5395-01 | 8.035 | 1 | 21.3 |
| TCGA-E1-A7YH-01 | 8.032 | 1 | 94.5 |
| TCGA-HT-8563-01 | 8.03 | 0 | 28.66666667 |
| TCGA-E1-5303-01 | 8.028 | 1 | 68.4 |
| TCGA-12-0619-01 | 8.023 | 1 | 35.4 |
| TCGA-HT-7860-01 | 8.019 | 0 | 0.5 |
| TCGA-HT-A61B-01 | 8.018 | 0 | 17.76666667 |
| TCGA-P5-A5EW-01 | 8.014 | 0 | 6.8 |
| TCGA-DU-6404-01 | 8.013 | 1 | 135.6 |
| TCGA-FN-7833-01 | 8.012 | 0 | 27.9 |
| TCGA-DU-6542-01 | 8.011 | 0 | 2.433333333 |
| TCGA-HT-7857-01 | 8.011 | 0 | 0.233333333 |
| TCGA-DB-5281-01 | 8.009 | 0 | 79.36666667 |
| TCGA-06-2565-01 | 8.004 | 1 | 16.86666667 |
| TCGA-S9-A6WG-01 | 8.003 | 0 | 86.73333333 |
| TCGA-DH-A66G-01 | 8.003 | 0 | 17.43333333 |
| TCGA-HT-7469-01 | 7.999 | 1 | 11.7 |
| TCGA-06-0171-02 | 7.997 | 1 | 13.3 |
| TCGA-S9-A7R4-01 | 7.996 | 0 | 30.46666667 |
| TCGA-DU-5854-01 | 7.996 | 0 | 8.566666667 |
| TCGA-S9-A6TZ-01 | 7.995 | 0 | 37.33333333 |
| TCGA-DU-A7TI-01 | 7.992 | 1 | 39.43333333 |
| TCGA-HT-7873-01 | 7.989 | 0 | 23.93333333 |
| TCGA-DB-5277-01 | 7.983 | 1 | 51.56666667 |
| TCGA-P5-A5EZ-01 | 7.981 | 0 | 2.333333333 |
| TCGA-S9-A7R8-01 | 7.978 | 1 | 32.03333333 |
| TCGA-DB-A4XB-01 | 7.973 | 0 | 30.63333333 |
| TCGA-HT-A5RB-01 | 7.968 | 0 | 0.1 |
| TCGA-HW-A5KK-01 | 7.964 | 1 | 12.93333333 |
| TCGA-FG-6690-01 | 7.959 | 0 | 43.13333333 |
| TCGA-DU-7015-01 | 7.955 | 0 | 92.03333333 |
| TCGA-DU-8163-01 | 7.955 | 0 | 20.96666667 |
| TCGA-DU-8166-01 | 7.942 | 0 | 17.2 |
| TCGA-HW-8320-01 | 7.933 | 0 | 40.56666667 |
| TCGA-HW-A5KM-01 | 7.931 | 0 | 21 |
| TCGA-HT-7884-01 | 7.928 | 0 | 11.43333333 |
| TCGA-HT-8108-01 | 7.924 | 0 | 2.533333333 |
| TCGA-E1-A7YE-01 | 7.921 | 1 | 29.53333333 |
| TCGA-CS-5393-01 | 7.909 | 0 | 40.73333333 |
| TCGA-HT-7902-01 | 7.899 | 0 | 31.86666667 |
| TCGA-QH-A6CW-01 | 7.894 | 0 | 13.8 |
| TCGA-HT-7858-01 | 7.892 | 0 | 51.33333333 |
| TCGA-HT-7854-01 | 7.891 | 0 | 40.03333333 |
| TCGA-E1-A7YW-01 | 7.889 | 1 | 37.33333333 |
| TCGA-DH-A66D-01 | 7.887 | 0 | 28.2 |
| TCGA-02-0047-01 | 7.885 | 1 | 14.93333333 |
| TCGA-S9-A6WO-01 | 7.872 | 0 | 18.86666667 |
| TCGA-QH-A6CS-01 | 7.868 | 0 | 19.6 |
| TCGA-DH-A7UU-01 | 7.867 | 0 | 13.9 |
| TCGA-DU-A76L-01 | 7.865 | 1 | 27.13333333 |
| TCGA-DH-5140-01 | 7.863 | 1 | 20.23333333 |
| TCGA-E1-A7YS-01 | 7.858 | 1 | 15.53333333 |
| TCGA-FG-A4MU-01 | 7.858 | 0 | 10.86666667 |
| TCGA-HT-A5RC-01 | 7.851 | 1 | 5.4 |
| TCGA-06-0129-01 | 7.84 | 1 | 34.13333333 |
| TCGA-HT-8106-01 | 7.838 | 0 | 0.1 |
| TCGA-HT-7472-01 | 7.829 | 0 | 0.033333333 |
| TCGA-HT-7473-01 | 7.823 | 0 | 16.76666667 |
| TCGA-S9-A6UA-01 | 7.822 | 1 | 5.933333333 |
| TCGA-FG-8182-01 | 7.821 | 0 | 13.86666667 |
| TCGA-DU-6403-01 | 7.817 | 1 | 11.8 |
| TCGA-S9-A6WM-01 | 7.817 | 0 | 20.13333333 |
| TCGA-KT-A7W1-01 | 7.807 | 0 | 14.6 |
| TCGA-DU-6406-01 | 7.799 | 1 | 17.06666667 |
| TCGA-HT-7882-01 | 7.787 | 1 | 3.766666667 |
| TCGA-TQ-A7RM-01 | 7.787 | 0 | 37.2 |
| TCGA-P5-A780-01 | 7.784 | 0 | 2.4 |
| TCGA-27-1830-01 | 7.776 | 1 | 5.133333333 |
| TCGA-S9-A89V-01 | 7.767 | 0 | 18.96666667 |
| TCGA-06-0190-01 | 7.748 | 1 | 10.56666667 |
| TCGA-CS-6665-01 | 7.745 | 0 | 52.26666667 |
| TCGA-FG-8191-01 | 7.743 | 0 | 33.06666667 |
| TCGA-DU-7012-01 | 7.741 | 1 | 6.633333333 |
| TCGA-TQ-A7RW-01 | 7.739 | 1 | 27.36666667 |
| TCGA-FG-A87N-01 | 7.722 | 0 | 18.9 |
| TCGA-DU-6396-01 | 7.712 | 1 | 76.2 |
| TCGA-DU-7010-01 | 7.691 | 1 | 15.2 |
| TCGA-DU-A7TD-01 | 7.683 | 1 | 7.6 |
| TCGA-19-1390-01 | 7.653 | 1 | 25.73333333 |
| TCGA-TM-A84H-01 | 7.634 | 0 | 30.86666667 |
| TCGA-06-0190-02 | 7.619 | 1 | 10.56666667 |
| TCGA-DU-7013-01 | 7.61 | 1 | 8.966666667 |
| TCGA-DU-A7TB-01 | 7.607 | 0 | 52.23333333 |
| TCGA-HW-A5KL-01 | 7.592 | 0 | 29.26666667 |
| TCGA-DB-A4XD-01 | 7.584 | 0 | 40.33333333 |
| TCGA-DU-7006-01 | 7.543 | 1 | 11.63333333 |
| TCGA-19-5960-01 | 7.541 | 1 | 15.16666667 |
| TCGA-TM-A84J-01 | 7.338 | 0 | 24.5 |
| TCGA-TM-A84I-01 | 7.28 | 0 | 28.46666667 |

**Table S5 The histological types, IDH1 mutation statuses and MGMT methylation statuses of the patients in TCGA LGG GBM cohort**

| **Samples** | **Histological type** | **B166** | **IDH1 (binary**  **non-silent**  **mutation call)** | **MGMT**  **(beta value)** |
| --- | --- | --- | --- | --- |
| TCGA-QH-A65X-01 | Oligoastrocytoma | 10.3 | 0 | 0.8392 |
| TCGA-DB-5274-01 | Oligoastrocytoma | 10.11 | 1 | 0.8449 |
| TCGA-HT-7684-01 | Oligoastrocytoma | 9.638 | 1 | 0.8376 |
| TCGA-HT-7681-01 | Oligoastrocytoma | 9.098 | 0 | 0.8406 |
| TCGA-KT-A74X-01 | Oligoastrocytoma | 9.002 | 1 | 0.8298 |
| TCGA-QH-A6CZ-01 | Oligoastrocytoma | 8.967 | 1 | 0.8423 |
| TCGA-P5-A730-01 | Oligoastrocytoma | 8.964 | 0 | 0.8405 |
| TCGA-QH-A65S-01 | Oligoastrocytoma | 8.947 | 1 | 0.8335 |
| TCGA-S9-A6WH-01 | Oligoastrocytoma | 8.944 | 1 | 0.8509 |
| TCGA-S9-A6TV-01 | Oligoastrocytoma | 8.906 | 1 | 0.7919 |
| TCGA-S9-A6WI-01 | Oligoastrocytoma | 8.882 | 1 | 0.7982 |
| TCGA-QH-A6X4-01 | Oligoastrocytoma | 8.88 | 1 | 0.8294 |
| TCGA-QH-A6X5-01 | Oligoastrocytoma | 8.87 | 1 | 0.8399 |
| TCGA-DB-A4X9-01 | Oligoastrocytoma | 8.858 | 1 | 0.8402 |
| TCGA-DB-A4XA-01 | Oligoastrocytoma | 8.839 | 1 | 0.8333 |
| TCGA-P5-A77X-01 | Oligoastrocytoma | 8.779 | 1 | 0.8319 |
| TCGA-QH-A6CV-01 | Oligoastrocytoma | 8.759 | 0 | 0.8217 |
| TCGA-HT-7608-01 | Oligoastrocytoma | 8.756 | 1 | 0.8596 |
| TCGA-S9-A7QY-01 | Oligoastrocytoma | 8.751 | 0 | 0.8299 |
| TCGA-HT-8018-01 | Oligoastrocytoma | 8.743 | 1 | 0.7967 |
| TCGA-FG-8181-01 | Oligoastrocytoma | 8.738 | 0 | 0.8171 |
| TCGA-HT-7692-01 | Oligoastrocytoma | 8.733 | 1 | 0.8569 |
| TCGA-FG-5965-02 | Oligoastrocytoma | 8.717 |  | 0.6915 |
| TCGA-P5-A731-01 | Oligoastrocytoma | 8.714 | 1 | 0.7995 |
| TCGA-S9-A7IQ-01 | Oligoastrocytoma | 8.691 | 1 | 0.8534 |
| TCGA-DU-5872-01 | Oligoastrocytoma | 8.691 | 1 | 0.7779 |
| TCGA-S9-A6WP-01 | Oligoastrocytoma | 8.69 | 1 | 0.86 |
| TCGA-HT-7690-01 | Oligoastrocytoma | 8.689 | 1 | 0.831 |
| TCGA-DU-A6S6-01 | Oligoastrocytoma | 8.665 | 1 | 0.842 |
| TCGA-S9-A7IY-01 | Oligoastrocytoma | 8.647 | 1 | 0.8457 |
| TCGA-FG-7643-01 | Oligoastrocytoma | 8.632 | 0 | 0.8186 |
| TCGA-FG-8186-01 | Oligoastrocytoma | 8.626 | 1 | 0.8563 |
| TCGA-FG-7637-01 | Oligoastrocytoma | 8.606 | 1 | 0.8278 |
| TCGA-CS-6186-01 | Oligoastrocytoma | 8.603 | 0 | 0.8345 |
| TCGA-DB-A64W-01 | Oligoastrocytoma | 8.602 | 1 | 0.8354 |
| TCGA-QH-A6CY-01 | Oligoastrocytoma | 8.594 | 1 | 0.8482 |
| TCGA-DB-5270-01 | Oligoastrocytoma | 8.588 | 1 | 0.798 |
| TCGA-DU-A5TR-01 | Oligoastrocytoma | 8.587 | 1 | 0.7917 |
| TCGA-DB-A64O-01 | Oligoastrocytoma | 8.585 | 0 | 0.8217 |
| TCGA-TM-A7C5-01 | Oligoastrocytoma | 8.584 | 1 | 0.8323 |
| TCGA-HW-7489-01 | Oligoastrocytoma | 8.577 | 1 | 0.8545 |
| TCGA-FG-A70Z-01 | Oligoastrocytoma | 8.56 | 0 | 0.6911 |
| TCGA-DU-6395-01 | Oligoastrocytoma | 8.548 | 1 | 0.8296 |
| TCGA-RY-A845-01 | Oligoastrocytoma | 8.54 | 1 | 0.8319 |
| TCGA-FG-A713-01 | Oligoastrocytoma | 8.522 | 1 | 0.824 |
| TCGA-DH-5143-01 | Oligoastrocytoma | 8.509 | 1 | 0.8538 |
| TCGA-P5-A737-01 | Oligoastrocytoma | 8.508 | 1 | 0.8481 |
| TCGA-DB-A75K-01 | Oligoastrocytoma | 8.503 | 1 | 0.8459 |
| TCGA-DU-7304-01 | Oligoastrocytoma | 8.494 | 1 | 0.7527 |
| TCGA-DB-5275-01 | Oligoastrocytoma | 8.493 | 1 | 0.8366 |
| TCGA-E1-A7YU-01 | Oligoastrocytoma | 8.493 | 1 | 0.8339 |
| TCGA-S9-A6WQ-01 | Oligoastrocytoma | 8.473 | 1 | 0.8567 |
| TCGA-HT-7475-01 | Oligoastrocytoma | 8.47 | 1 | 0.8507 |
| TCGA-DB-A64S-01 | Oligoastrocytoma | 8.469 | 1 | 0.8298 |
| TCGA-IK-8125-01 | Oligoastrocytoma | 8.466 | 1 | 0.8459 |
| TCGA-HT-7474-01 | Oligoastrocytoma | 8.464 | 1 | 0.8407 |
| TCGA-VV-A829-01 | Oligoastrocytoma | 8.458 | 1 | 0.828 |
| TCGA-P5-A77W-01 | Oligoastrocytoma | 8.452 | 1 | 0.8603 |
| TCGA-FG-8187-01 | Oligoastrocytoma | 8.436 | 1 | 0.8339 |
| TCGA-DU-7306-01 | Oligoastrocytoma | 8.435 | 1 | 0.7558 |
| TCGA-HT-7483-01 | Oligoastrocytoma | 8.434 | 1 | 0.7345 |
| TCGA-DB-A4XE-01 | Oligoastrocytoma | 8.431 | 1 | 0.8459 |
| TCGA-QH-A6XA-01 | Oligoastrocytoma | 8.427 | 1 | 0.8465 |
| TCGA-DB-A64Q-01 | Oligoastrocytoma | 8.426 | 1 | 0.846 |
| TCGA-FG-A4MW-01 | Oligoastrocytoma | 8.425 | 0 | 0.6526 |
| TCGA-WY-A85D-01 | Oligoastrocytoma | 8.406 | 1 | 0.8335 |
| TCGA-HT-7880-01 | Oligoastrocytoma | 8.406 | 1 | 0.8317 |
| TCGA-DU-5871-01 | Oligoastrocytoma | 8.371 | 1 | 0.7964 |
| TCGA-TQ-A7RG-01 | Oligoastrocytoma | 8.358 | 1 | 0.8343 |
| TCGA-DU-7019-01 | Oligoastrocytoma | 8.35 | 1 | 0.8098 |
| TCGA-DU-8161-01 | Oligoastrocytoma | 8.35 | 0 | 0.8288 |
| TCGA-DU-A7T8-01 | Oligoastrocytoma | 8.346 | 1 | 0.8353 |
| TCGA-DU-8167-01 | Oligoastrocytoma | 8.344 | 1 | 0.8196 |
| TCGA-TQ-A7RK-01 | Oligoastrocytoma | 8.342 | 1 | 0.8527 |
| TCGA-TM-A84L-01 | Oligoastrocytoma | 8.337 | 1 | 0.8351 |
| TCGA-QH-A870-01 | Oligoastrocytoma | 8.317 | 1 | 0.8262 |
| TCGA-FG-8188-01 | Oligoastrocytoma | 8.315 | 1 | 0.8225 |
| TCGA-DU-8162-01 | Oligoastrocytoma | 8.303 | 0 | 0.8111 |
| TCGA-DU-5852-01 | Oligoastrocytoma | 8.271 | 0 | 0.6768 |
| TCGA-HT-A74J-01 | Oligoastrocytoma | 8.269 | 1 | 0.8231 |
| TCGA-FG-A60J-01 | Oligoastrocytoma | 8.253 | 1 | 0.8477 |
| TCGA-DU-7011-01 | Oligoastrocytoma | 8.246 | 1 | 0.8247 |
| TCGA-DU-5872-02 | Oligoastrocytoma | 8.217 |  | 0.8231 |
| TCGA-HT-A614-01 | Oligoastrocytoma | 8.214 | 1 | 0.8248 |
| TCGA-FG-A4MY-01 | Oligoastrocytoma | 8.212 | 1 | 0.7685 |
| TCGA-HT-8013-01 | Oligoastrocytoma | 8.207 | 1 | 0.8499 |
| TCGA-E1-5322-01 | Oligoastrocytoma | 8.203 | 1 | 0.7298 |
| TCGA-FG-5965-01 | Oligoastrocytoma | 8.201 | 1 | 0.8027 |
| TCGA-HT-8114-01 | Oligoastrocytoma | 8.198 | 1 | 0.8355 |
| TCGA-WY-A85E-01 | Oligoastrocytoma | 8.198 | 1 | 0.6473 |
| TCGA-DU-5855-01 | Oligoastrocytoma | 8.195 | 1 | 0.8161 |
| TCGA-TQ-A7RO-01 | Oligoastrocytoma | 8.186 | 0 | 0.839 |
| TCGA-HT-8111-01 | Oligoastrocytoma | 8.185 | 1 | 0.8244 |
| TCGA-DB-A64U-01 | Oligoastrocytoma | 8.178 | 1 | 0.8473 |
| TCGA-TQ-A7RJ-01 | Oligoastrocytoma | 8.175 | 1 | 0.7701 |
| TCGA-FG-A60K-01 | Oligoastrocytoma | 8.175 | 1 | 0.8614 |
| TCGA-TQ-A7RP-01 | Oligoastrocytoma | 8.174 | 0 | 0.8232 |
| TCGA-HT-A74L-01 | Oligoastrocytoma | 8.166 | 1 | 0.8227 |
| TCGA-HT-7610-01 | Oligoastrocytoma | 8.161 | 1 | 0.8086 |
| TCGA-E1-A7YV-01 | Oligoastrocytoma | 8.15 | 1 | 0.4702 |
| TCGA-HT-7611-01 | Oligoastrocytoma | 8.148 | 1 | 0.8518 |
| TCGA-TM-A84T-01 | Oligoastrocytoma | 8.125 | 1 | 0.8479 |
| TCGA-DB-5280-01 | Oligoastrocytoma | 8.122 | 1 | 0.8626 |
| TCGA-DB-5276-01 | Oligoastrocytoma | 8.106 | 1 | 0.8255 |
| TCGA-DU-5851-01 | Oligoastrocytoma | 8.102 | 1 | 0.7681 |
| TCGA-HT-7482-01 | Oligoastrocytoma | 8.098 | 1 | 0.8359 |
| TCGA-QH-A6X3-01 | Oligoastrocytoma | 8.08 | 1 | 0.7998 |
| TCGA-DB-A4XH-01 | Oligoastrocytoma | 8.08 | 1 | 0.7506 |
| TCGA-HT-7879-01 | Oligoastrocytoma | 8.079 | 1 | 0.8216 |
| TCGA-DU-5853-01 | Oligoastrocytoma | 8.069 | 1 | 0.7922 |
| TCGA-DB-A4XC-01 | Oligoastrocytoma | 8.067 | 1 | 0.8222 |
| TCGA-TQ-A7RH-01 | Oligoastrocytoma | 8.059 | 1 | 0.7867 |
| TCGA-TQ-A7RR-01 | Oligoastrocytoma | 8.058 | 1 | 0.8171 |
| TCGA-HT-7609-01 | Oligoastrocytoma | 8.057 | 1 | 0.8597 |
| TCGA-FN-7833-01 | Oligoastrocytoma | 8.012 | 1 | 0.8406 |
| TCGA-DU-6542-01 | Oligoastrocytoma | 8.011 | 1 | 0.8577 |
| TCGA-DB-5281-01 | Oligoastrocytoma | 8.009 | 1 | 0.8169 |
| TCGA-TQ-A7RK-02 | Oligoastrocytoma | 7.991 |  | 0.8337 |
| TCGA-HT-7873-01 | Oligoastrocytoma | 7.989 | 1 | 0.8506 |
| TCGA-DU-8163-01 | Oligoastrocytoma | 7.955 | 1 | 0.8385 |
| TCGA-DU-8166-01 | Oligoastrocytoma | 7.942 | 1 | 0.8496 |
| TCGA-HT-7902-01 | Oligoastrocytoma | 7.899 | 1 | 0.7784 |
| TCGA-QH-A6CW-01 | Oligoastrocytoma | 7.894 | 1 | 0.8492 |
| TCGA-E1-A7YW-01 | Oligoastrocytoma | 7.889 | 1 | 0.8394 |
| TCGA-DH-5140-01 | Oligoastrocytoma | 7.863 | 0 | 0.5327 |
| TCGA-FG-A4MU-01 | Oligoastrocytoma | 7.858 | 0 | 0.6907 |
| TCGA-HT-7473-01 | Oligoastrocytoma | 7.823 | 1 | 0.8347 |
| TCGA-DU-6403-01 | Oligoastrocytoma | 7.817 | 0 | 0.7531 |
| TCGA-DU-6406-01 | Oligoastrocytoma | 7.799 | 0 | 0.7684 |
| TCGA-TQ-A7RM-01 | Oligoastrocytoma | 7.787 | 1 | 0.4824 |
| TCGA-DU-6396-01 | Oligoastrocytoma | 7.712 | 1 | 0.7626 |
| TCGA-DU-A7TD-01 | Oligoastrocytoma | 7.683 | 0 | 0.7588 |
| TCGA-TM-A84H-01 | Oligoastrocytoma | 7.634 | 1 | 0.8188 |
| TCGA-FG-A6IZ-01 | Oligodendroglioma | 10.37 | 1 | 0.8704 |
| TCGA-FG-A6J1-01 | Oligodendroglioma | 10.16 | 1 | 0.8513 |
| TCGA-HT-A74K-01 | Oligodendroglioma | 10.15 | 1 | 0.7688 |
| TCGA-R8-A73M-01 | Oligodendroglioma | 9.885 | 1 | 0.8606 |
| TCGA-P5-A5F6-01 | Oligodendroglioma | 9.84 | 0 | 0.795 |
| TCGA-DH-A669-02 | Oligodendroglioma | 9.796 |  | 0.8514 |
| TCGA-RY-A840-01 | Oligodendroglioma | 9.487 | 1 | 0.8514 |
| TCGA-S9-A6TW-01 | Oligodendroglioma | 9.453 | 1 | 0.8309 |
| TCGA-RY-A83Y-01 | Oligodendroglioma | 9.438 | 1 | 0.8638 |
| TCGA-VM-A8CA-01 | Oligodendroglioma | 9.376 | 1 | 0.8363 |
| TCGA-VM-A8CB-01 | Oligodendroglioma | 9.371 | 1 | 0.8338 |
| TCGA-HT-7603-01 | Oligodendroglioma | 9.246 | 1 | 0.8372 |
| TCGA-DU-A6S8-01 | Oligodendroglioma | 9.228 | 1 | 0.8384 |
| TCGA-DH-5141-01 | Oligodendroglioma | 9.222 | 0 | 0.875 |
| TCGA-DU-5870-02 | Oligodendroglioma | 9.196 |  | 0.7949 |
| TCGA-QH-A6CU-01 | Oligodendroglioma | 9.191 | 1 | 0.8453 |
| TCGA-HT-7693-01 | Oligodendroglioma | 9.162 | 1 | 0.82 |
| TCGA-DH-A7UR-01 | Oligodendroglioma | 9.068 | 0 | 0.6257 |
| TCGA-DU-5874-01 | Oligodendroglioma | 9.054 | 1 | 0.8762 |
| TCGA-DU-7018-01 | Oligodendroglioma | 9.03 | 1 | 0.803 |
| TCGA-S9-A6TY-01 | Oligodendroglioma | 9.003 | 0 | 0.8499 |
| TCGA-QH-A65Z-01 | Oligodendroglioma | 8.976 | 1 | 0.8731 |
| TCGA-HT-A4DV-01 | Oligodendroglioma | 8.972 | 1 | 0.837 |
| TCGA-VW-A7QS-01 | Oligodendroglioma | 8.954 | 1 | 0.8121 |
| TCGA-DB-A64L-01 | Oligodendroglioma | 8.951 | 1 | 0.8579 |
| TCGA-F6-A8O3-01 | Oligodendroglioma | 8.936 | 1 | 0.8296 |
| TCGA-DU-A7T6-01 | Oligodendroglioma | 8.91 | 1 | 0.7726 |
| TCGA-FG-5964-01 | Oligodendroglioma | 8.886 | 1 | 0.8406 |
| TCGA-QH-A65V-01 | Oligodendroglioma | 8.885 | 1 | 0.8533 |
| TCGA-VM-A8C8-01 | Oligodendroglioma | 8.857 | 1 | 0.8397 |
| TCGA-QH-A65R-01 | Oligodendroglioma | 8.846 | 1 | 0.7754 |
| TCGA-DU-6394-01 | Oligodendroglioma | 8.842 | 1 | 0.8449 |
| TCGA-S9-A6WD-01 | Oligodendroglioma | 8.832 | 1 | 0.826 |
| TCGA-HT-A617-01 | Oligodendroglioma | 8.82 | 0 | 0.8127 |
| TCGA-FG-A710-01 | Oligodendroglioma | 8.804 | 1 | 0.8245 |
| TCGA-FG-5962-01 | Oligodendroglioma | 8.802 | 1 | 0.8674 |
| TCGA-DU-A76R-01 | Oligodendroglioma | 8.777 | 1 | 0.8364 |
| TCGA-HW-A5KJ-01 | Oligodendroglioma | 8.767 | 1 | 0.8433 |
| TCGA-HT-7605-01 | Oligodendroglioma | 8.762 | 1 | 0.8423 |
| TCGA-R8-A6MO-01 | Oligodendroglioma | 8.76 | 1 | 0.8307 |
| TCGA-S9-A6TX-01 | Oligodendroglioma | 8.76 | 1 | 0.8296 |
| TCGA-E1-A7YY-01 | Oligodendroglioma | 8.759 | 1 | 0.8162 |
| TCGA-DB-A64V-01 | Oligodendroglioma | 8.755 | 1 | 0.8539 |
| TCGA-DB-A64P-01 | Oligodendroglioma | 8.754 | 1 | 0.8582 |
| TCGA-TM-A84R-01 | Oligodendroglioma | 8.752 | 0 | 0.8308 |
| TCGA-S9-A6UB-01 | Oligodendroglioma | 8.75 | 1 | 0.8509 |
| TCGA-P5-A5F4-01 | Oligodendroglioma | 8.742 | 1 | 0.8338 |
| TCGA-DU-6393-01 | Oligodendroglioma | 8.742 | 1 | 0.8581 |
| TCGA-DU-A76K-01 | Oligodendroglioma | 8.739 | 0 | 0.8107 |
| TCGA-E1-5318-01 | Oligodendroglioma | 8.734 | 0 | 0.7905 |
| TCGA-DU-6400-01 | Oligodendroglioma | 8.728 | 1 | 0.8732 |
| TCGA-DH-5144-01 | Oligodendroglioma | 8.724 | 1 | 0.8723 |
| TCGA-HT-A61C-01 | Oligodendroglioma | 8.724 | 0 | 0.6787 |
| TCGA-P5-A5EX-01 | Oligodendroglioma | 8.718 | 1 | 0.8109 |
| TCGA-E1-A7Z2-01 | Oligodendroglioma | 8.71 | 0 | 0.816 |
| TCGA-HT-7471-01 | Oligodendroglioma | 8.708 | 1 | 0.869 |
| TCGA-TQ-A7RU-01 | Oligodendroglioma | 8.699 | 1 | 0.8496 |
| TCGA-HT-8019-01 | Oligodendroglioma | 8.695 |  | 0.8122 |
| TCGA-DU-A6S3-01 | Oligodendroglioma | 8.694 | 1 | 0.8498 |
| TCGA-R8-A6ML-01 | Oligodendroglioma | 8.69 | 1 | 0.8601 |
| TCGA-TQ-A7RF-01 | Oligodendroglioma | 8.69 | 1 | 0.8125 |
| TCGA-P5-A5ET-01 | Oligodendroglioma | 8.683 | 1 | 0.8449 |
| TCGA-FG-7638-01 | Oligodendroglioma | 8.678 | 1 | 0.8417 |
| TCGA-TM-A84S-01 | Oligodendroglioma | 8.678 | 1 | 0.8372 |
| TCGA-TQ-A7RN-01 | Oligodendroglioma | 8.664 | 1 | 0.827 |
| TCGA-HT-7694-01 | Oligodendroglioma | 8.661 | 1 | 0.8597 |
| TCGA-E1-5319-01 | Oligodendroglioma | 8.657 | 1 | 0.8628 |
| TCGA-FG-A4MT-01 | Oligodendroglioma | 8.656 | 1 | 0.8596 |
| TCGA-QH-A6X8-01 | Oligodendroglioma | 8.651 | 1 | 0.6375 |
| TCGA-TQ-A7RS-01 | Oligodendroglioma | 8.649 | 1 | 0.8017 |
| TCGA-TM-A84G-01 | Oligodendroglioma | 8.647 | 1 | 0.8434 |
| TCGA-HT-7616-01 | Oligodendroglioma | 8.646 | 1 | 0.8369 |
| TCGA-DU-7008-01 | Oligodendroglioma | 8.639 | 1 | 0.7295 |
| TCGA-RY-A847-01 | Oligodendroglioma | 8.635 | 1 | 0.8322 |
| TCGA-EZ-7264-01 | Oligodendroglioma | 8.633 | 1 | 0.8679 |
| TCGA-S9-A6WE-01 | Oligodendroglioma | 8.632 | 1 | 0.8338 |
| TCGA-FG-A711-01 | Oligodendroglioma | 8.626 | 1 | 0.7622 |
| TCGA-HT-7676-01 | Oligodendroglioma | 8.608 | 1 | 0.8639 |
| TCGA-CS-6670-01 | Oligodendroglioma | 8.594 | 1 | 0.8528 |
| TCGA-FG-6692-01 | Oligodendroglioma | 8.593 | 0 | 0.6957 |
| TCGA-DH-A66F-01 | Oligodendroglioma | 8.592 | 1 | 0.8658 |
| TCGA-DB-5279-01 | Oligodendroglioma | 8.589 | 1 | 0.7676 |
| TCGA-TQ-A8XE-02 | Oligodendroglioma | 8.587 |  | 0.8134 |
| TCGA-HW-7487-01 | Oligodendroglioma | 8.578 | 1 | 0.8551 |
| TCGA-HT-8113-01 | Oligodendroglioma | 8.571 | 0 | 0.8279 |
| TCGA-DU-6397-02 | Oligodendroglioma | 8.571 |  | 0.8145 |
| TCGA-FG-A4MT-02 | Oligodendroglioma | 8.567 |  | 0.8257 |
| TCGA-R8-A6MK-01 | Oligodendroglioma | 8.562 | 1 | 0.856 |
| TCGA-DU-6407-02 | Oligodendroglioma | 8.554 |  | 0.7217 |
| TCGA-DU-5849-01 | Oligodendroglioma | 8.547 | 0 | 0.8511 |
| TCGA-W9-A837-01 | Oligodendroglioma | 8.541 | 1 | 0.8344 |
| TCGA-HT-7688-01 | Oligodendroglioma | 8.537 | 1 | 0.7642 |
| TCGA-RY-A83X-01 | Oligodendroglioma | 8.536 | 1 | 0.8615 |
| TCGA-DU-7309-01 | Oligodendroglioma | 8.523 | 0 | 0.7375 |
| TCGA-DU-6397-01 | Oligodendroglioma | 8.522 | 1 | 0.8497 |
| TCGA-HT-A615-01 | Oligodendroglioma | 8.521 | 1 | 0.8516 |
| TCGA-HT-7481-01 | Oligodendroglioma | 8.514 | 1 | 0.8502 |
| TCGA-DU-5870-01 | Oligodendroglioma | 8.512 | 1 | 0.8408 |
| TCGA-DU-A5TS-01 | Oligodendroglioma | 8.506 | 1 | 0.7856 |
| TCGA-HT-8105-01 | Oligodendroglioma | 8.505 | 1 | 0.8638 |
| TCGA-FG-8189-01 | Oligodendroglioma | 8.505 | 0 | 0.794 |
| TCGA-DU-8164-01 | Oligodendroglioma | 8.504 | 1 | 0.861 |
| TCGA-HT-8010-01 | Oligodendroglioma | 8.504 | 1 | 0.854 |
| TCGA-P5-A72Z-01 | Oligodendroglioma | 8.504 | 1 | 0.837 |
| TCGA-P5-A5F0-01 | Oligodendroglioma | 8.5 | 1 | 0.8211 |
| TCGA-HT-A61A-01 | Oligodendroglioma | 8.5 | 0 | 0.8277 |
| TCGA-DB-5278-01 | Oligodendroglioma | 8.497 | 1 | 0.8369 |
| TCGA-TQ-A8XE-01 | Oligodendroglioma | 8.493 | 1 | 0.8182 |
| TCGA-HW-7491-01 | Oligodendroglioma | 8.493 | 0 | 0.8613 |
| TCGA-HT-A5R9-01 | Oligodendroglioma | 8.488 | 0 | 0.6362 |
| TCGA-CS-5396-01 | Oligodendroglioma | 8.473 | 1 | 0.8478 |
| TCGA-DU-7294-01 | Oligodendroglioma | 8.465 | 1 | 0.8676 |
| TCGA-DB-A4XG-01 | Oligodendroglioma | 8.461 | 0 | 0.8533 |
| TCGA-S9-A7J0-01 | Oligodendroglioma | 8.456 | 1 | 0.6615 |
| TCGA-DU-7009-01 | Oligodendroglioma | 8.446 | 1 | 0.8627 |
| TCGA-DU-A5TT-01 | Oligodendroglioma | 8.446 | 0 | 0.7893 |
| TCGA-S9-A7R1-01 | Oligodendroglioma | 8.442 | 1 | 0.8679 |
| TCGA-HW-7486-01 | Oligodendroglioma | 8.438 | 1 | 0.8484 |
| TCGA-HT-7695-01 | Oligodendroglioma | 8.437 | 1 | 0.8521 |
| TCGA-DU-A7TG-01 | Oligodendroglioma | 8.436 | 1 | 0.8328 |
| TCGA-DH-A669-01 | Oligodendroglioma | 8.435 | 1 | 0.8521 |
| TCGA-DH-A7US-01 | Oligodendroglioma | 8.435 | 1 | 0.8227 |
| TCGA-DU-7302-01 | Oligodendroglioma | 8.429 | 1 | 0.8099 |
| TCGA-HT-7856-01 | Oligodendroglioma | 8.424 | 0 | 0.8262 |
| TCGA-HT-8558-01 | Oligodendroglioma | 8.421 | 0 | 0.8021 |
| TCGA-TM-A84O-01 | Oligodendroglioma | 8.42 | 1 | 0.8564 |
| TCGA-HT-7874-01 | Oligodendroglioma | 8.416 | 1 | 0.8404 |
| TCGA-DU-6399-01 | Oligodendroglioma | 8.411 | 1 | 0.732 |
| TCGA-HT-7687-01 | Oligodendroglioma | 8.39 | 1 | 0.8616 |
| TCGA-IK-7675-01 | Oligodendroglioma | 8.389 | 1 | 0.6821 |
| TCGA-HT-7602-01 | Oligodendroglioma | 8.381 | 1 | 0.8243 |
| TCGA-P5-A72U-01 | Oligodendroglioma | 8.379 | 0 | 0.8069 |
| TCGA-DB-A64R-01 | Oligodendroglioma | 8.365 | 1 | 0.8454 |
| TCGA-DU-7300-01 | Oligodendroglioma | 8.364 | 1 | 0.8553 |
| TCGA-HT-7677-01 | Oligodendroglioma | 8.362 | 1 | 0.8525 |
| TCGA-CS-6669-01 | Oligodendroglioma | 8.358 |  | 0.8078 |
| TCGA-HT-A5R5-01 | Oligodendroglioma | 8.357 | 1 | 0.835 |
| TCGA-HT-8012-01 | Oligodendroglioma | 8.355 | 1 | 0.7961 |
| TCGA-S9-A7QZ-01 | Oligodendroglioma | 8.354 | 1 | 0.8602 |
| TCGA-HT-8107-01 | Oligodendroglioma | 8.351 | 0 | 0.8034 |
| TCGA-DU-6410-01 | Oligodendroglioma | 8.341 | 1 | 0.8673 |
| TCGA-HT-7881-01 | Oligodendroglioma | 8.341 | 1 | 0.8365 |
| TCGA-HT-8109-01 | Oligodendroglioma | 8.338 | 1 | 0.8412 |
| TCGA-QH-A86X-01 | Oligodendroglioma | 8.32 | 1 | 0.8317 |
| TCGA-S9-A7J2-01 | Oligodendroglioma | 8.318 | 1 | 0.8588 |
| TCGA-HT-7875-01 | Oligodendroglioma | 8.317 | 1 | 0.8557 |
| TCGA-DU-6408-01 | Oligodendroglioma | 8.315 | 1 | 0.7609 |
| TCGA-TM-A84M-01 | Oligodendroglioma | 8.303 | 1 | 0.8219 |
| TCGA-HT-7467-01 | Oligodendroglioma | 8.302 | 1 | 0.8315 |
| TCGA-VM-A8CE-01 | Oligodendroglioma | 8.295 | 1 | 0.8289 |
| TCGA-DU-A7TA-01 | Oligodendroglioma | 8.291 | 0 | 0.8133 |
| TCGA-HW-7495-01 | Oligodendroglioma | 8.289 | 1 | 0.8569 |
| TCGA-DU-7014-01 | Oligodendroglioma | 8.283 |  | 0.7963 |
| TCGA-DU-7301-01 | Oligodendroglioma | 8.275 | 1 | 0.836 |
| TCGA-HT-7689-01 | Oligodendroglioma | 8.272 | 1 | 0.8344 |
| TCGA-DU-6401-01 | Oligodendroglioma | 8.254 | 1 | 0.7729 |
| TCGA-HT-7877-01 | Oligodendroglioma | 8.252 | 1 | 0.794 |
| TCGA-DU-8168-01 | Oligodendroglioma | 8.249 | 1 | 0.8758 |
| TCGA-HT-7468-01 | Oligodendroglioma | 8.23 | 1 | 0.8603 |
| TCGA-FG-A70Y-01 | Oligodendroglioma | 8.222 | 1 | 0.771 |
| TCGA-S9-A7J3-01 | Oligodendroglioma | 8.22 | 1 | 0.8497 |
| TCGA-QH-A6X9-01 | Oligodendroglioma | 8.22 | 1 | 0.7674 |
| TCGA-HT-7480-01 | Oligodendroglioma | 8.215 | 1 | 0.8514 |
| TCGA-S9-A6U2-01 | Oligodendroglioma | 8.211 | 1 | 0.8369 |
| TCGA-E1-5311-01 | Oligodendroglioma | 8.21 | 1 | 0.8552 |
| TCGA-HT-7620-01 | Oligodendroglioma | 8.209 | 1 | 0.827 |
| TCGA-TQ-A7RI-01 | Oligodendroglioma | 8.204 | 1 | 0.8035 |
| TCGA-S9-A7J1-01 | Oligodendroglioma | 8.199 | 1 | 0.8554 |
| TCGA-DU-A6S2-01 | Oligodendroglioma | 8.192 | 1 | 0.8443 |
| TCGA-DU-6407-01 | Oligodendroglioma | 8.169 | 1 | 0.8311 |
| TCGA-FG-7641-01 | Oligodendroglioma | 8.168 | 1 | 0.8412 |
| TCGA-TQ-A7RQ-01 | Oligodendroglioma | 8.167 | 1 | 0.8291 |
| TCGA-HT-7470-01 | Oligodendroglioma | 8.16 | 1 | 0.8178 |
| TCGA-E1-A7YQ-01 | Oligodendroglioma | 8.149 | 0 | 0.8299 |
| TCGA-HW-8322-01 | Oligodendroglioma | 8.142 | 1 | 0.8538 |
| TCGA-FG-7634-01 | Oligodendroglioma | 8.139 | 1 | 0.815 |
| TCGA-CS-5390-01 | Oligodendroglioma | 8.12 | 1 | 0.7518 |
| TCGA-E1-A7YO-01 | Oligodendroglioma | 8.119 | 1 | 0.8464 |
| TCGA-HT-A619-01 | Oligodendroglioma | 8.104 | 1 | 0.7965 |
| TCGA-CS-6668-01 | Oligodendroglioma | 8.053 | 1 | 0.8681 |
| TCGA-DU-8165-01 | Oligodendroglioma | 8.042 | 0 | 0.7929 |
| TCGA-CS-5395-01 | Oligodendroglioma | 8.035 | 0 | 0.8056 |
| TCGA-DU-6404-02 | Oligodendroglioma | 8.034 |  | 0.7624 |
| TCGA-DU-6404-01 | Oligodendroglioma | 8.013 | 0 | 0.7974 |
| TCGA-DH-A66G-01 | Oligodendroglioma | 8.003 | 1 | 0.7895 |
| TCGA-HT-7469-01 | Oligodendroglioma | 7.999 | 0 | 0.6526 |
| TCGA-FG-6690-01 | Oligodendroglioma | 7.959 | 1 | 0.8515 |
| TCGA-DU-7015-01 | Oligodendroglioma | 7.955 | 1 | 0.8607 |
| TCGA-HT-8108-01 | Oligodendroglioma | 7.924 | 1 | 0.8211 |
| TCGA-DU-A76L-01 | Oligodendroglioma | 7.865 | 0 | 0.7586 |
| TCGA-E1-A7YS-01 | Oligodendroglioma | 7.858 | 1 | 0.8521 |
| TCGA-HT-7472-01 | Oligodendroglioma | 7.829 | 1 | 0.8737 |
| TCGA-FG-8182-01 | Oligodendroglioma | 7.821 | 1 | 0.8423 |
| TCGA-HT-7882-01 | Oligodendroglioma | 7.787 | 0 | 0.7943 |
| TCGA-FG-8191-01 | Oligodendroglioma | 7.743 | 1 | 0.8399 |
| TCGA-TQ-A7RW-01 | Oligodendroglioma | 7.739 | 1 | 0.8141 |
| TCGA-DU-A7TB-01 | Oligodendroglioma | 7.607 | 0 | 0.6805 |
| TCGA-TM-A84J-01 | Oligodendroglioma | 7.338 | 0 | 0.6632 |
| TCGA-FG-A60L-01 | Astrocytoma | 9.942 | 1 | 0.7825 |
| TCGA-RY-A83Z-01 | Astrocytoma | 9.923 | 1 | 0.5362 |
| TCGA-HT-A4DS-01 | Astrocytoma | 9.802 | 0 | 0.7917 |
| TCGA-FG-A6J3-01 | Astrocytoma | 9.795 | 1 | 0.6208 |
| TCGA-VM-A8C9-01 | Astrocytoma | 9.752 | 0 | 0.822 |
| TCGA-VM-A8CF-01 | Astrocytoma | 9.706 | 1 | 0.8289 |
| TCGA-DU-7290-01 | Astrocytoma | 9.516 | 0 | 0.8286 |
| TCGA-HW-7490-01 | Astrocytoma | 9.239 | 1 | 0.8523 |
| TCGA-P5-A72W-01 | Astrocytoma | 9.136 | 1 | 0.8353 |
| TCGA-WY-A859-01 | Astrocytoma | 9.073 | 1 | 0.7666 |
| TCGA-DU-A7TC-01 | Astrocytoma | 9.005 | 1 | 0.8535 |
| TCGA-HT-A74O-01 | Astrocytoma | 8.948 | 1 | 0.7843 |
| TCGA-P5-A5F1-01 | Astrocytoma | 8.936 | 1 | 0.8228 |
| TCGA-QH-A6XC-01 | Astrocytoma | 8.913 | 0 | 0.7417 |
| TCGA-CS-4944-01 | Astrocytoma | 8.894 | 1 | 0.8566 |
| TCGA-HT-7606-01 | Astrocytoma | 8.826 | 1 | 0.7899 |
| TCGA-DB-A75P-01 | Astrocytoma | 8.818 | 0 | 0.7834 |
| TCGA-P5-A735-01 | Astrocytoma | 8.804 | 1 | 0.8471 |
| TCGA-HT-A74H-01 | Astrocytoma | 8.786 | 0 | 0.7781 |
| TCGA-DB-A75L-01 | Astrocytoma | 8.779 | 1 | 0.8143 |
| TCGA-DU-A76O-01 | Astrocytoma | 8.776 | 1 | 0.839 |
| TCGA-P5-A5EV-01 | Astrocytoma | 8.771 | 1 | 0.8483 |
| TCGA-S9-A6U5-01 | Astrocytoma | 8.765 | 1 | 0.822 |
| TCGA-HT-7476-01 | Astrocytoma | 8.762 | 0 | 0.6008 |
| TCGA-FG-A4MX-01 | Astrocytoma | 8.736 | 1 | 0.8306 |
| TCGA-DU-A5TU-01 | Astrocytoma | 8.731 | 1 | 0.757 |
| TCGA-DB-A75O-01 | Astrocytoma | 8.729 | 1 | 0.8389 |
| TCGA-DB-A64X-01 | Astrocytoma | 8.724 | 1 | 0.7428 |
| TCGA-FG-7636-01 | Astrocytoma | 8.718 | 1 | 0.8517 |
| TCGA-S9-A6TS-01 | Astrocytoma | 8.713 | 1 | 0.682 |
| TCGA-F6-A8O4-01 | Astrocytoma | 8.705 | 1 | 0.834 |
| TCGA-S9-A6U6-01 | Astrocytoma | 8.687 | 1 | 0.8167 |
| TCGA-FG-8185-01 | Astrocytoma | 8.672 | 1 | 0.8184 |
| TCGA-WH-A86K-01 | Astrocytoma | 8.669 | 1 | 0.7907 |
| TCGA-P5-A736-01 | Astrocytoma | 8.66 | 1 | 0.829 |
| TCGA-HT-7691-01 | Astrocytoma | 8.644 | 0 | 0.8071 |
| TCGA-HT-A5R7-01 | Astrocytoma | 8.636 | 1 | 0.7226 |
| TCGA-DB-A75M-01 | Astrocytoma | 8.632 | 1 | 0.8508 |
| TCGA-P5-A733-01 | Astrocytoma | 8.631 | 1 | 0.7133 |
| TCGA-DU-A5TW-01 | Astrocytoma | 8.623 | 1 | 0.6998 |
| TCGA-P5-A781-01 | Astrocytoma | 8.619 | 1 | 0.8385 |
| TCGA-S9-A7QW-01 | Astrocytoma | 8.618 | 1 | 0.815 |
| TCGA-QH-A6CX-01 | Astrocytoma | 8.61 | 0 | 0.81 |
| TCGA-CS-5394-01 | Astrocytoma | 8.607 | 1 | 0.8759 |
| TCGA-S9-A89Z-01 | Astrocytoma | 8.584 | 1 | 0.8053 |
| TCGA-VM-A8CD-01 | Astrocytoma | 8.584 | 0 | 0.8137 |
| TCGA-HT-7607-01 | Astrocytoma | 8.581 | 1 | 0.8222 |
| TCGA-WY-A85A-01 | Astrocytoma | 8.558 | 1 | 0.7249 |
| TCGA-DU-7292-01 | Astrocytoma | 8.555 | 0 | 0.7322 |
| TCGA-DH-5142-01 | Astrocytoma | 8.553 | 1 | 0.8382 |
| TCGA-P5-A5EY-01 | Astrocytoma | 8.55 | 0 | 0.8091 |
| TCGA-DU-5847-01 | Astrocytoma | 8.542 | 0 | 0.8176 |
| TCGA-P5-A5F2-01 | Astrocytoma | 8.54 | 1 | 0.8347 |
| TCGA-TM-A7C4-01 | Astrocytoma | 8.533 | 1 | 0.7815 |
| TCGA-CS-5397-01 | Astrocytoma | 8.531 | 0 | 0.8271 |
| TCGA-FG-6688-01 | Astrocytoma | 8.526 | 0 | 0.8124 |
| TCGA-E1-A7Z3-01 | Astrocytoma | 8.512 | 1 | 0.8378 |
| TCGA-DH-A66B-01 | Astrocytoma | 8.494 | 1 | 0.8312 |
| TCGA-S9-A7R2-01 | Astrocytoma | 8.493 | 0 | 0.8125 |
| TCGA-CS-6667-01 | Astrocytoma | 8.48 | 1 | 0.8569 |
| TCGA-HT-A616-01 | Astrocytoma | 8.473 | 1 | 0.7609 |
| TCGA-DU-7007-01 | Astrocytoma | 8.472 | 1 | 0.8288 |
| TCGA-S9-A7IZ-01 | Astrocytoma | 8.472 | 1 | 0.7695 |
| TCGA-FG-A87Q-01 | Astrocytoma | 8.463 | 0 | 0.7772 |
| TCGA-HT-8015-01 | Astrocytoma | 8.461 | 0 | 0.8001 |
| TCGA-S9-A7IX-01 | Astrocytoma | 8.459 | 0 | 0.8239 |
| TCGA-CS-6666-01 | Astrocytoma | 8.443 | 1 | 0.7084 |
| TCGA-WY-A858-01 | Astrocytoma | 8.438 | 1 | 0.7397 |
| TCGA-S9-A7R3-01 | Astrocytoma | 8.434 | 1 | 0.8551 |
| TCGA-S9-A6U0-01 | Astrocytoma | 8.424 | 0 | 0.7278 |
| TCGA-FG-6689-01 | Astrocytoma | 8.422 | 1 | 0.8427 |
| TCGA-P5-A72X-01 | Astrocytoma | 8.415 | 1 | 0.8 |
| TCGA-E1-5305-01 | Astrocytoma | 8.41 | 1 | 0.8593 |
| TCGA-CS-4942-01 | Astrocytoma | 8.406 | 1 | 0.8165 |
| TCGA-S9-A7QX-01 | Astrocytoma | 8.403 | 1 | 0.7236 |
| TCGA-DU-7299-01 | Astrocytoma | 8.397 | 1 | 0.8232 |
| TCGA-HT-8104-01 | Astrocytoma | 8.396 | 0 | 0.8008 |
| TCGA-S9-A6WL-01 | Astrocytoma | 8.391 | 1 | 0.8468 |
| TCGA-CS-4943-01 | Astrocytoma | 8.39 | 1 | 0.7631 |
| TCGA-E1-5302-01 | Astrocytoma | 8.373 | 1 | 0.8485 |
| TCGA-DU-6402-01 | Astrocytoma | 8.368 | 0 | 0.8316 |
| TCGA-E1-A7YK-01 | Astrocytoma | 8.36 | 1 | 0.8152 |
| TCGA-DU-6392-01 | Astrocytoma | 8.354 | 0 | 0.7845 |
| TCGA-S9-A7IS-01 | Astrocytoma | 8.345 | 1 | 0.5973 |
| TCGA-CS-4938-01 | Astrocytoma | 8.344 | 1 | 0.8429 |
| TCGA-P5-A5EU-01 | Astrocytoma | 8.341 | 1 | 0.801 |
| TCGA-TM-A84Q-01 | Astrocytoma | 8.339 | 1 | 0.8305 |
| TCGA-HT-7604-01 | Astrocytoma | 8.338 | 1 | 0.837 |
| TCGA-HT-8011-01 | Astrocytoma | 8.326 | 0 | 0.761 |
| TCGA-E1-A7YM-01 | Astrocytoma | 8.305 | 0 | 0.8364 |
| TCGA-TM-A84F-01 | Astrocytoma | 8.296 | 1 | 0.7922 |
| TCGA-TM-A7CF-02 | Astrocytoma | 8.293 |  | 0.818 |
| TCGA-E1-5304-01 | Astrocytoma | 8.292 | 1 | 0.8229 |
| TCGA-DB-5273-01 | Astrocytoma | 8.29 | 1 | 0.8621 |
| TCGA-E1-5307-01 | Astrocytoma | 8.289 | 1 | 0.8828 |
| TCGA-DU-A6S7-01 | Astrocytoma | 8.285 | 1 | 0.7072 |
| TCGA-E1-A7YN-01 | Astrocytoma | 8.285 | 0 | 0.7755 |
| TCGA-TM-A84B-01 | Astrocytoma | 8.268 | 0 | 0.808 |
| TCGA-HT-8564-01 | Astrocytoma | 8.266 | 0 | 0.7611 |
| TCGA-DH-A7UV-01 | Astrocytoma | 8.252 | 1 | 0.8425 |
| TCGA-WY-A85B-01 | Astrocytoma | 8.249 | 1 | 0.8343 |
| TCGA-HT-7485-01 | Astrocytoma | 8.247 | 1 | 0.8635 |
| TCGA-HW-7493-01 | Astrocytoma | 8.247 |  | 0.8206 |
| TCGA-HT-7478-01 | Astrocytoma | 8.245 | 1 | 0.8499 |
| TCGA-E1-A7YJ-01 | Astrocytoma | 8.244 | 0 | 0.8223 |
| TCGA-HT-7479-01 | Astrocytoma | 8.238 | 1 | 0.8477 |
| TCGA-E1-A7Z4-01 | Astrocytoma | 8.235 | 1 | 0.8554 |
| TCGA-RY-A843-01 | Astrocytoma | 8.235 | 1 | 0.8216 |
| TCGA-HW-8319-01 | Astrocytoma | 8.232 | 1 | 0.8055 |
| TCGA-VV-A86M-01 | Astrocytoma | 8.229 | 1 | 0.866 |
| TCGA-HT-7477-01 | Astrocytoma | 8.217 | 1 | 0.6722 |
| TCGA-TQ-A7RV-01 | Astrocytoma | 8.216 | 1 | 0.8537 |
| TCGA-S9-A6TU-01 | Astrocytoma | 8.211 | 1 | 0.8465 |
| TCGA-HT-A5RA-01 | Astrocytoma | 8.21 | 0 | 0.7716 |
| TCGA-DU-7298-01 | Astrocytoma | 8.207 | 1 | 0.8723 |
| TCGA-VM-A8CH-01 | Astrocytoma | 8.202 | 1 | 0.8062 |
| TCGA-S9-A6U1-01 | Astrocytoma | 8.197 | 1 | 0.7773 |
| TCGA-DH-A7UT-01 | Astrocytoma | 8.191 | 1 | 0.8218 |
| TCGA-TM-A7CA-01 | Astrocytoma | 8.191 | 1 | 0.8032 |
| TCGA-WY-A85C-01 | Astrocytoma | 8.175 | 1 | 0.706 |
| TCGA-FG-5963-01 | Astrocytoma | 8.173 | 0 | 0.7466 |
| TCGA-HT-7686-01 | Astrocytoma | 8.172 | 1 | 0.7883 |
| TCGA-HT-7680-01 | Astrocytoma | 8.171 | 0 | 0.7825 |
| TCGA-HT-A618-01 | Astrocytoma | 8.17 | 0 | 0.8059 |
| TCGA-VW-A8FI-01 | Astrocytoma | 8.162 | 0 | 0.8111 |
| TCGA-S9-A6U9-01 | Astrocytoma | 8.155 | 1 | 0.8423 |
| TCGA-E1-A7YD-01 | Astrocytoma | 8.155 | 0 | 0.7103 |
| TCGA-CS-6290-01 | Astrocytoma | 8.152 | 1 | 0.8342 |
| TCGA-FG-6691-01 | Astrocytoma | 8.147 | 1 | 0.8298 |
| TCGA-S9-A7R7-01 | Astrocytoma | 8.145 | 1 | 0.8467 |
| TCGA-CS-6188-01 | Astrocytoma | 8.142 | 0 | 0.8234 |
| TCGA-DU-6405-01 | Astrocytoma | 8.142 | 0 | 0.751 |
| TCGA-TQ-A7RV-02 | Astrocytoma | 8.13 |  | 0.8391 |
| TCGA-S9-A6U8-01 | Astrocytoma | 8.119 | 1 | 0.8473 |
| TCGA-E1-A7Z6-01 | Astrocytoma | 8.117 | 1 | 0.8143 |
| TCGA-DU-8158-01 | Astrocytoma | 8.112 | 0 | 0.813 |
| TCGA-DU-A5TY-01 | Astrocytoma | 8.111 | 0 | 0.7783 |
| TCGA-E1-A7YL-01 | Astrocytoma | 8.11 | 0 | 0.805 |
| TCGA-CS-4941-01 | Astrocytoma | 8.109 | 0 | 0.7413 |
| TCGA-DU-A7TJ-01 | Astrocytoma | 8.107 | 0 | 0.6707 |
| TCGA-TM-A7C3-01 | Astrocytoma | 8.104 | 0 | 0.8098 |
| TCGA-DU-A5TP-01 | Astrocytoma | 8.101 | 1 | 0.8342 |
| TCGA-HW-8321-01 | Astrocytoma | 8.094 | 1 | 0.7921 |
| TCGA-TM-A7CF-01 | Astrocytoma | 8.092 | 1 | 0.8428 |
| TCGA-HT-7601-01 | Astrocytoma | 8.088 | 1 | 0.805 |
| TCGA-DB-A4XF-01 | Astrocytoma | 8.083 | 1 | 0.8432 |
| TCGA-E1-A7YI-01 | Astrocytoma | 8.069 | 1 | 0.5491 |
| TCGA-HT-7855-01 | Astrocytoma | 8.064 | 1 | 0.8221 |
| TCGA-TM-A84C-01 | Astrocytoma | 8.047 | 0 | 0.774 |
| TCGA-S9-A6WN-01 | Astrocytoma | 8.042 | 1 | 0.8327 |
| TCGA-HT-8110-01 | Astrocytoma | 8.039 | 0 | 0.731 |
| TCGA-E1-A7YH-01 | Astrocytoma | 8.032 | 1 | 0.7716 |
| TCGA-HT-8563-01 | Astrocytoma | 8.03 | 1 | 0.841 |
| TCGA-E1-5303-01 | Astrocytoma | 8.028 | 1 | 0.8382 |
| TCGA-FG-5963-02 | Astrocytoma | 8.02 |  | 0.7122 |
| TCGA-HT-7860-01 | Astrocytoma | 8.019 | 0 | 0.8 |
| TCGA-HT-A61B-01 | Astrocytoma | 8.018 | 1 | 0.7859 |
| TCGA-P5-A5EW-01 | Astrocytoma | 8.014 | 1 | 0.7766 |
| TCGA-HT-7857-01 | Astrocytoma | 8.011 | 0 | 0.7238 |
| TCGA-S9-A6WG-01 | Astrocytoma | 8.003 | 1 | 0.8242 |
| TCGA-S9-A7R4-01 | Astrocytoma | 7.996 | 1 | 0.8469 |
| TCGA-DU-5854-01 | Astrocytoma | 7.996 | 0 | 0.8192 |
| TCGA-S9-A6TZ-01 | Astrocytoma | 7.995 | 1 | 0.8555 |
| TCGA-DU-A7TI-01 | Astrocytoma | 7.992 |  | 0.8404 |
| TCGA-DB-5277-01 | Astrocytoma | 7.983 | 1 | 0.547 |
| TCGA-P5-A5EZ-01 | Astrocytoma | 7.981 | 1 | 0.8144 |
| TCGA-S9-A7R8-01 | Astrocytoma | 7.978 | 1 | 0.8543 |
| TCGA-DB-A4XB-01 | Astrocytoma | 7.973 | 1 | 0.8378 |
| TCGA-HT-A5RB-01 | Astrocytoma | 7.968 | 1 | 0.8534 |
| TCGA-HW-A5KK-01 | Astrocytoma | 7.964 | 0 | 0.8115 |
| TCGA-HW-8320-01 | Astrocytoma | 7.933 | 1 | 0.8209 |
| TCGA-HW-A5KM-01 | Astrocytoma | 7.931 | 1 | 0.8627 |
| TCGA-HT-7884-01 | Astrocytoma | 7.928 | 1 | 0.8445 |
| TCGA-E1-A7YE-01 | Astrocytoma | 7.921 | 1 | 0.6431 |
| TCGA-CS-5393-01 | Astrocytoma | 7.909 | 1 | 0.8254 |
| TCGA-HT-7858-01 | Astrocytoma | 7.892 | 1 | 0.8515 |
| TCGA-HT-7854-01 | Astrocytoma | 7.891 | 0 | 0.8263 |
| TCGA-DH-A66D-01 | Astrocytoma | 7.887 | 1 | 0.8523 |
| TCGA-S9-A6WO-01 | Astrocytoma | 7.872 | 1 | 0.8522 |
| TCGA-QH-A6CS-01 | Astrocytoma | 7.868 | 0 | 0.8147 |
| TCGA-DH-A7UU-01 | Astrocytoma | 7.867 | 1 | 0.6957 |
| TCGA-HT-A5RC-01 | Astrocytoma | 7.851 | 0 | 0.8185 |
| TCGA-HT-8106-01 | Astrocytoma | 7.838 | 1 | 0.8189 |
| TCGA-S9-A6UA-01 | Astrocytoma | 7.822 | 0 | 0.7272 |
| TCGA-S9-A6WM-01 | Astrocytoma | 7.817 | 0 | 0.7779 |
| TCGA-KT-A7W1-01 | Astrocytoma | 7.807 | 0 | 0.6778 |
| TCGA-P5-A780-01 | Astrocytoma | 7.784 | 1 | 0.7498 |
| TCGA-S9-A89V-01 | Astrocytoma | 7.767 | 0 | 0.7618 |
| TCGA-CS-6665-01 | Astrocytoma | 7.745 | 1 | 0.6013 |
| TCGA-DU-7012-01 | Astrocytoma | 7.741 | 0 | 0.7837 |
| TCGA-FG-A87N-01 | Astrocytoma | 7.722 | 1 | 0.7021 |
| TCGA-DU-7010-01 | Astrocytoma | 7.691 | 1 | 0.6039 |
| TCGA-DU-7013-01 | Astrocytoma | 7.61 | 0 | 0.8233 |
| TCGA-HW-A5KL-01 | Astrocytoma | 7.592 | 1 | 0.8469 |
| TCGA-DB-A4XD-01 | Astrocytoma | 7.584 | 1 | 0.84 |
| TCGA-DU-7006-01 | Astrocytoma | 7.543 | 0 | 0.7188 |
| TCGA-TM-A84I-01 | Astrocytoma | 7.28 | 1 | 0.6692 |
| TCGA-15-1444-01 | Glioblastoma Multiforme (GBM) | 8.557 |  | 0.6401 |
| TCGA-14-0736-02 | Untreated primary (de novo) GBM | 10.1 |  | 0.8126 |
| TCGA-27-2526-01 | Untreated primary (de novo) GBM | 10.01 | 0 |  |
| TCGA-27-1831-01 | Untreated primary (de novo) GBM | 9.896 | 0 |  |
| TCGA-12-3652-01 | Untreated primary (de novo) GBM | 9.844 | 0 |  |
| TCGA-06-0178-01 | Untreated primary (de novo) GBM | 9.827 |  |  |
| TCGA-28-5213-01 | Untreated primary (de novo) GBM | 9.795 | 0 | 0.8253 |
| TCGA-06-0219-01 | Untreated primary (de novo) GBM | 9.735 | 0 |  |
| TCGA-14-1829-01 | Untreated primary (de novo) GBM | 9.724 | 0 |  |
| TCGA-76-4929-01 | Untreated primary (de novo) GBM | 9.578 | 0 | 0.6914 |
| TCGA-41-2572-01 | Untreated primary (de novo) GBM | 9.54 | 0 |  |
| TCGA-27-1832-01 | Untreated primary (de novo) GBM | 9.504 | 0 |  |
| TCGA-14-1402-02 | Untreated primary (de novo) GBM | 9.431 |  | 0.7273 |
| TCGA-12-5295-01 | Untreated primary (de novo) GBM | 9.417 | 0 | 0.6497 |
| TCGA-06-0749-01 | Untreated primary (de novo) GBM | 9.371 | 0 |  |
| TCGA-06-5411-01 | Untreated primary (de novo) GBM | 9.346 | 0 | 0.7242 |
| TCGA-06-5416-01 | Untreated primary (de novo) GBM | 9.345 | 0 | 0.5996 |
| TCGA-27-2524-01 | Untreated primary (de novo) GBM | 9.328 | 0 |  |
| TCGA-12-1597-01 | Untreated primary (de novo) GBM | 9.288 | 0 |  |
| TCGA-26-5136-01 | Untreated primary (de novo) GBM | 9.273 | 0 | 0.7208 |
| TCGA-06-0745-01 | Untreated primary (de novo) GBM | 9.271 | 0 |  |
| TCGA-06-2569-01 | Untreated primary (de novo) GBM | 9.266 | 0 |  |
| TCGA-32-4213-01 | Untreated primary (de novo) GBM | 9.259 | 0 |  |
| TCGA-27-2519-01 | Untreated primary (de novo) GBM | 9.239 | 0 |  |
| TCGA-28-5220-01 | Untreated primary (de novo) GBM | 9.217 | 0 | 0.7759 |
| TCGA-26-5132-01 | Untreated primary (de novo) GBM | 9.203 | 0 | 0.7244 |
| TCGA-14-2554-01 | Untreated primary (de novo) GBM | 9.188 | 0 |  |
| TCGA-06-0168-01 | Untreated primary (de novo) GBM | 9.182 | 0 |  |
| TCGA-06-0750-01 | Untreated primary (de novo) GBM | 9.173 | 0 |  |
| TCGA-06-0132-01 | Untreated primary (de novo) GBM | 9.154 | 0 |  |
| TCGA-06-0744-01 | Untreated primary (de novo) GBM | 9.141 | 0 |  |
| TCGA-06-2567-01 | Untreated primary (de novo) GBM | 9.092 | 0 |  |
| TCGA-27-2521-01 | Untreated primary (de novo) GBM | 9.064 | 1 |  |
| TCGA-14-0817-01 | Untreated primary (de novo) GBM | 9.042 | 0 |  |
| TCGA-06-0743-01 | Untreated primary (de novo) GBM | 9.028 | 0 |  |
| TCGA-28-2499-01 | Untreated primary (de novo) GBM | 9.022 | 0 |  |
| TCGA-76-4931-01 | Untreated primary (de novo) GBM | 9.009 | 0 | 0.8002 |
| TCGA-02-2486-01 | Untreated primary (de novo) GBM | 9.009 | 0 |  |
| TCGA-12-5299-01 | Untreated primary (de novo) GBM | 8.983 | 0 | 0.848 |
| TCGA-06-5859-01 | Untreated primary (de novo) GBM | 8.974 | 0 | 0.7866 |
| TCGA-06-5418-01 | Untreated primary (de novo) GBM | 8.972 | 0 | 0.8221 |
| TCGA-08-0386-01 | Untreated primary (de novo) GBM | 8.969 | 0 |  |
| TCGA-06-5415-01 | Untreated primary (de novo) GBM | 8.944 | 0 | 0.8132 |
| TCGA-12-3650-01 | Untreated primary (de novo) GBM | 8.938 | 0 |  |
| TCGA-14-1823-01 | Untreated primary (de novo) GBM | 8.937 | 0 |  |
| TCGA-06-5413-01 | Untreated primary (de novo) GBM | 8.921 | 0 | 0.8036 |
| TCGA-76-4925-01 | Untreated primary (de novo) GBM | 8.92 | 0 | 0.7183 |
| TCGA-06-0184-01 | Untreated primary (de novo) GBM | 8.916 | 0 |  |
| TCGA-19-2629-01 | Untreated primary (de novo) GBM | 8.9 | 1 |  |
| TCGA-41-2571-01 | Untreated primary (de novo) GBM | 8.893 | 0 |  |
| TCGA-14-0787-01 | Untreated primary (de novo) GBM | 8.884 | 0 |  |
| TCGA-14-0790-01 | Untreated primary (de novo) GBM | 8.858 | 0 |  |
| TCGA-32-2616-01 | Untreated primary (de novo) GBM | 8.857 | 0 |  |
| TCGA-28-5204-01 | Untreated primary (de novo) GBM | 8.855 | 0 | 0.8369 |
| TCGA-32-1980-01 | Untreated primary (de novo) GBM | 8.842 | 0 | 0.81 |
| TCGA-26-1442-01 | Untreated primary (de novo) GBM | 8.84 | 1 | 0.7084 |
| TCGA-26-5133-01 | Untreated primary (de novo) GBM | 8.835 | 0 | 0.7805 |
| TCGA-06-0882-01 | Untreated primary (de novo) GBM | 8.83 | 0 |  |
| TCGA-32-5222-01 | Untreated primary (de novo) GBM | 8.829 | 0 | 0.7493 |
| TCGA-19-2625-01 | Untreated primary (de novo) GBM | 8.828 | 0 |  |
| TCGA-28-2513-01 | Untreated primary (de novo) GBM | 8.826 | 0 |  |
| TCGA-06-5412-01 | Untreated primary (de novo) GBM | 8.796 | 0 | 0.8021 |
| TCGA-06-0138-01 | Untreated primary (de novo) GBM | 8.795 |  |  |
| TCGA-19-1389-02 | Untreated primary (de novo) GBM | 8.793 |  | 0.8259 |
| TCGA-06-5408-01 | Untreated primary (de novo) GBM | 8.789 | 0 | 0.7954 |
| TCGA-06-0649-01 | Untreated primary (de novo) GBM | 8.754 | 0 |  |
| TCGA-02-2483-01 | Untreated primary (de novo) GBM | 8.75 | 1 |  |
| TCGA-06-1804-01 | Untreated primary (de novo) GBM | 8.748 | 0 | 0.7246 |
| TCGA-28-5208-01 | Untreated primary (de novo) GBM | 8.743 | 0 | 0.7744 |
| TCGA-06-0238-01 | Untreated primary (de novo) GBM | 8.743 | 0 |  |
| TCGA-15-0742-01 | Untreated primary (de novo) GBM | 8.734 | 0 |  |
| TCGA-06-0156-01 | Untreated primary (de novo) GBM | 8.727 |  |  |
| TCGA-19-2624-01 | Untreated primary (de novo) GBM | 8.714 | 0 |  |
| TCGA-28-1747-01 | Untreated primary (de novo) GBM | 8.712 | 0 |  |
| TCGA-32-1982-01 | Untreated primary (de novo) GBM | 8.707 | 0 |  |
| TCGA-06-0141-01 | Untreated primary (de novo) GBM | 8.705 | 0 |  |
| TCGA-27-2528-01 | Untreated primary (de novo) GBM | 8.677 | 0 |  |
| TCGA-06-5410-01 | Untreated primary (de novo) GBM | 8.67 | 0 | 0.8106 |
| TCGA-76-4927-01 | Untreated primary (de novo) GBM | 8.667 | 0 | 0.8169 |
| TCGA-02-0055-01 | Untreated primary (de novo) GBM | 8.667 | 0 |  |
| TCGA-14-0781-01 | Untreated primary (de novo) GBM | 8.666 | 0 | 0.8071 |
| TCGA-06-2558-01 | Untreated primary (de novo) GBM | 8.66 | 0 |  |
| TCGA-32-2632-01 | Untreated primary (de novo) GBM | 8.658 | 0 |  |
| TCGA-28-5216-01 | Untreated primary (de novo) GBM | 8.657 | 0 | 0.6123 |
| TCGA-14-1825-01 | Untreated primary (de novo) GBM | 8.655 | 0 |  |
| TCGA-12-0821-01 | Untreated primary (de novo) GBM | 8.638 | 0 |  |
| TCGA-32-1970-01 | Untreated primary (de novo) GBM | 8.638 | 0 |  |
| TCGA-76-4926-01 | Untreated primary (de novo) GBM | 8.624 | 0 | 0.8078 |
| TCGA-12-0616-01 | Untreated primary (de novo) GBM | 8.614 | 0 |  |
| TCGA-32-2634-01 | Untreated primary (de novo) GBM | 8.609 | 0 |  |
| TCGA-76-4928-01 | Untreated primary (de novo) GBM | 8.592 | 0 | 0.7928 |
| TCGA-06-0130-01 | Untreated primary (de novo) GBM | 8.59 | 0 |  |
| TCGA-19-1787-01 | Untreated primary (de novo) GBM | 8.588 | 0 |  |
| TCGA-26-5135-01 | Untreated primary (de novo) GBM | 8.579 | 0 | 0.6057 |
| TCGA-06-0211-02 | Untreated primary (de novo) GBM | 8.56 |  | 0.7838 |
| TCGA-32-2638-01 | Untreated primary (de novo) GBM | 8.556 | 0 |  |
| TCGA-06-0646-01 | Untreated primary (de novo) GBM | 8.544 | 0 |  |
| TCGA-06-0211-01 | Untreated primary (de novo) GBM | 8.537 | 0 | 0.7712 |
| TCGA-06-0644-01 | Untreated primary (de novo) GBM | 8.537 | 0 |  |
| TCGA-27-2523-01 | Untreated primary (de novo) GBM | 8.532 | 0 |  |
| TCGA-28-2514-01 | Untreated primary (de novo) GBM | 8.531 | 0 |  |
| TCGA-19-2620-01 | Untreated primary (de novo) GBM | 8.527 | 0 |  |
| TCGA-27-1835-01 | Untreated primary (de novo) GBM | 8.524 | 0 |  |
| TCGA-27-1837-01 | Untreated primary (de novo) GBM | 8.522 | 0 |  |
| TCGA-26-5139-01 | Untreated primary (de novo) GBM | 8.517 | 0 | 0.7889 |
| TCGA-28-2509-01 | Untreated primary (de novo) GBM | 8.513 | 0 |  |
| TCGA-41-4097-01 | Untreated primary (de novo) GBM | 8.508 | 0 |  |
| TCGA-06-0878-01 | Untreated primary (de novo) GBM | 8.497 | 0 |  |
| TCGA-06-5417-01 | Untreated primary (de novo) GBM | 8.495 | 1 | 0.423 |
| TCGA-26-5134-01 | Untreated primary (de novo) GBM | 8.477 | 0 | 0.703 |
| TCGA-14-1034-01 | Untreated primary (de novo) GBM | 8.46 | 0 |  |
| TCGA-19-2619-01 | Untreated primary (de novo) GBM | 8.457 | 0 |  |
| TCGA-28-5209-01 | Untreated primary (de novo) GBM | 8.452 | 0 | 0.7724 |
| TCGA-06-0157-01 | Untreated primary (de novo) GBM | 8.451 | 0 |  |
| TCGA-12-0618-01 | Untreated primary (de novo) GBM | 8.42 | 0 |  |
| TCGA-06-0187-01 | Untreated primary (de novo) GBM | 8.417 | 0 |  |
| TCGA-02-2485-01 | Untreated primary (de novo) GBM | 8.411 | 0 |  |
| TCGA-06-2563-01 | Untreated primary (de novo) GBM | 8.394 | 0 |  |
| TCGA-12-3653-01 | Untreated primary (de novo) GBM | 8.387 | 0 |  |
| TCGA-41-3915-01 | Untreated primary (de novo) GBM | 8.384 | 0 |  |
| TCGA-14-0871-01 | Untreated primary (de novo) GBM | 8.383 | 0 |  |
| TCGA-19-4065-02 | Untreated primary (de novo) GBM | 8.382 |  | 0.8051 |
| TCGA-27-1834-01 | Untreated primary (de novo) GBM | 8.356 | 0 |  |
| TCGA-06-0645-01 | Untreated primary (de novo) GBM | 8.355 | 0 |  |
| TCGA-06-2561-01 | Untreated primary (de novo) GBM | 8.348 | 0 |  |
| TCGA-16-0846-01 | Untreated primary (de novo) GBM | 8.332 | 0 |  |
| TCGA-06-5856-01 | Untreated primary (de novo) GBM | 8.321 | 0 | 0.7794 |
| TCGA-06-0686-01 | Untreated primary (de novo) GBM | 8.315 | 0 |  |
| TCGA-06-5414-01 | Untreated primary (de novo) GBM | 8.307 | 0 | 0.813 |
| TCGA-28-5218-01 | Untreated primary (de novo) GBM | 8.3 | 0 | 0.8064 |
| TCGA-06-2559-01 | Untreated primary (de novo) GBM | 8.292 | 0 |  |
| TCGA-06-2562-01 | Untreated primary (de novo) GBM | 8.284 | 0 |  |
| TCGA-06-0210-01 | Untreated primary (de novo) GBM | 8.277 | 0 | 0.7764 |
| TCGA-06-0174-01 | Untreated primary (de novo) GBM | 8.274 | 0 |  |
| TCGA-32-2615-01 | Untreated primary (de novo) GBM | 8.251 | 0 |  |
| TCGA-06-2570-01 | Untreated primary (de novo) GBM | 8.248 | 1 |  |
| TCGA-14-0789-01 | Untreated primary (de novo) GBM | 8.24 | 0 |  |
| TCGA-41-5651-01 | Untreated primary (de novo) GBM | 8.238 | 0 | 0.764 |
| TCGA-06-2557-01 | Untreated primary (de novo) GBM | 8.236 | 0 |  |
| TCGA-06-0158-01 | Untreated primary (de novo) GBM | 8.227 | 0 |  |
| TCGA-16-1045-01 | Untreated primary (de novo) GBM | 8.212 | 0 |  |
| TCGA-19-4065-01 | Untreated primary (de novo) GBM | 8.192 | 0 | 0.7543 |
| TCGA-28-5215-01 | Untreated primary (de novo) GBM | 8.189 | 0 | 0.6491 |
| TCGA-06-0125-02 | Untreated primary (de novo) GBM | 8.187 |  | 0.7932 |
| TCGA-14-1034-02 | Untreated primary (de novo) GBM | 8.186 |  | 0.795 |
| TCGA-06-0747-01 | Untreated primary (de novo) GBM | 8.155 | 0 |  |
| TCGA-28-5207-01 | Untreated primary (de novo) GBM | 8.137 | 0 | 0.7907 |
| TCGA-06-5858-01 | Untreated primary (de novo) GBM | 8.118 | 0 | 0.7508 |
| TCGA-06-0125-01 | Untreated primary (de novo) GBM | 8.118 | 0 | 0.7208 |
| TCGA-06-0152-02 | Untreated primary (de novo) GBM | 8.11 |  | 0.8066 |
| TCGA-19-0957-02 | Untreated primary (de novo) GBM | 8.064 |  | 0.7788 |
| TCGA-28-1753-01 | Untreated primary (de novo) GBM | 8.06 | 0 |  |
| TCGA-06-2564-01 | Untreated primary (de novo) GBM | 8.052 | 0 |  |
| TCGA-12-0619-01 | Untreated primary (de novo) GBM | 8.023 | 0 |  |
| TCGA-06-2565-01 | Untreated primary (de novo) GBM | 8.004 | 0 |  |
| TCGA-06-0171-02 | Untreated primary (de novo) GBM | 7.997 |  | 0.8349 |
| TCGA-06-0210-02 | Untreated primary (de novo) GBM | 7.937 |  | 0.7235 |
| TCGA-06-0221-02 | Untreated primary (de novo) GBM | 7.93 |  | 0.5741 |
| TCGA-02-0047-01 | Untreated primary (de novo) GBM | 7.885 | 0 |  |
| TCGA-06-0129-01 | Untreated primary (de novo) GBM | 7.84 | 1 |  |
| TCGA-27-1830-01 | Untreated primary (de novo) GBM | 7.776 | 0 |  |
| TCGA-06-0190-01 | Untreated primary (de novo) GBM | 7.748 | 0 | 0.8181 |
| TCGA-19-1390-01 | Untreated primary (de novo) GBM | 7.653 | 0 |  |
| TCGA-06-0190-02 | Untreated primary (de novo) GBM | 7.619 |  | 0.8093 |
| TCGA-19-5960-01 | Untreated primary (de novo) GBM | 7.541 | 0 | 0.8094 |

**Table S6 The comprehensive information of IDH1 mutation of the patients in TCGA LGG GBM cohort**

| **Sample** | **chr** | **reference** | **alt** | **effect** | **aminoAcid** | **dnaVaf** |
| --- | --- | --- | --- | --- | --- | --- |
| TCGA-QH-A65X-01 | no variant | no variant | no variant | no variant | no variant | no variant |
| TCGA-DB-5274-01 | chr2 | C | T | Missense_Mutation | p.R132H | 0.29 |
| TCGA-HT-7684-01 | chr2 | C | T | Missense_Mutation | p.R132H | 0.44 |
| TCGA-HT-7681-01 | no variant | no variant | no variant | no variant | no variant | no variant |
| TCGA-KT-A74X-01 | chr2 | C | T | Missense_Mutation | p.R132H | 0.33 |
| TCGA-QH-A6CZ-01 | chr2 | C | T | Missense_Mutation | p.R132H | 0.51 |
| TCGA-P5-A730-01 | no variant | no variant | no variant | no variant | no variant | no variant |
| TCGA-QH-A65S-01 | chr2 | C | T | Missense_Mutation | p.R132H | 0.39 |
| TCGA-S9-A6WH-01 | chr2 | C | T | Missense_Mutation | p.R132H | 0.39 |
| TCGA-S9-A6TV-01 | chr2 | C | T | Missense_Mutation | p.R132H | 0.36 |
| TCGA-S9-A6WI-01 | chr2 | G | A | Missense_Mutation | p.R132C | 0.11 |
| TCGA-QH-A6X4-01 | chr2 | C | T | Missense_Mutation | p.R132H | 0.29 |
| TCGA-QH-A6X5-01 | chr2 | C | T | Missense_Mutation | p.R132H | 0.17 |
| TCGA-DB-A4X9-01 | chr2 | C | T | Missense_Mutation | p.R132H | 0.38 |
| TCGA-DB-A4XA-01 | chr2 | C | T | Missense_Mutation | p.R132H | 0.28 |
| TCGA-P5-A77X-01 | chr2 | C | T | Missense_Mutation | p.R132H | 0.34 |
| TCGA-QH-A6CV-01 | no variant | no variant | no variant | no variant | no variant | no variant |
| TCGA-HT-7608-01 | chr2 | C | T | Missense_Mutation | p.R132H | 0.35 |
| TCGA-S9-A7QY-01 | no variant | no variant | no variant | no variant | no variant | no variant |
| TCGA-HT-8018-01 | chr2 | C | T | Missense_Mutation | p.R132H | 0.16 |
| TCGA-FG-8181-01 | no variant | no variant | no variant | no variant | no variant | no variant |
| TCGA-HT-7692-01 | chr2 | C | T | Missense_Mutation | p.R132H | 0.23 |
| TCGA-P5-A731-01 | chr2 | C | T | Missense_Mutation | p.R132H | 0.31 |
| TCGA-S9-A7IQ-01 | chr2 | C | T | Missense_Mutation | p.R132H | 0.19 |
| TCGA-DU-5872-01 | chr2 | C | T | Missense_Mutation | p.R132H | 0.39 |
| TCGA-S9-A6WP-01 | chr2 | C | T | Missense_Mutation | p.R132H | 0.37 |
| TCGA-HT-7690-01 | chr2 | C | T | Missense_Mutation | p.R132H | 0.36 |
| TCGA-DU-A6S6-01 | chr2 | C | T | Missense_Mutation | p.R132H | 0.29 |
| TCGA-S9-A7IY-01 | chr2 | C | T | Missense_Mutation | p.R132H | 0.36 |
| TCGA-FG-7643-01 | no variant | no variant | no variant | no variant | no variant | no variant |
| TCGA-FG-8186-01 | chr2 | C | T | Missense_Mutation | p.R132H | 0.29 |
| TCGA-FG-7637-01 | chr2 | C | T | Missense_Mutation | p.R132H | 0.46 |
| TCGA-CS-6186-01 | no variant | no variant | no variant | no variant | no variant | no variant |
| TCGA-DB-A64W-01 | chr2 | C | T | Missense_Mutation | p.R132H | 0.43 |
| TCGA-QH-A6CY-01 | chr2 | C | T | Missense_Mutation | p.R132H | 0.42 |
| TCGA-DB-5270-01 | chr2 | C | T | Missense_Mutation | p.R132H | 0.31 |
| TCGA-DU-A5TR-01 | chr2 | G | T | Missense_Mutation | p.R132S | 0.43 |
| TCGA-DB-A64O-01 | no variant | no variant | no variant | no variant | no variant | no variant |
| TCGA-TM-A7C5-01 | chr2 | C | T | Missense_Mutation | p.R132H | 0.42 |
| TCGA-HW-7489-01 | chr2 | C | T | Missense_Mutation | p.R132H | 0.24 |
| TCGA-FG-A70Z-01 | no variant | no variant | no variant | no variant | no variant | no variant |
| TCGA-DU-6395-01 | chr2 | C | T | Missense_Mutation | p.R132H | 0.37 |
| TCGA-RY-A845-01 | chr2 | C | T | Missense_Mutation | p.R132H | 0.33 |
| TCGA-FG-A713-01 | chr2 | C | T | Missense_Mutation | p.R132H | 0.16 |
| TCGA-DH-5143-01 | chr2 | C | T | Missense_Mutation | p.R132H | 0.31 |
| TCGA-P5-A737-01 | chr2 | C | T | Missense_Mutation | p.R132H | 0.62 |
| TCGA-DB-A75K-01 | chr2 | C | T | Missense_Mutation | p.R132H | 0.44 |
| TCGA-DU-7304-01 | chr2 | G | C | Missense_Mutation | p.R132G | 0.35 |
| TCGA-DB-5275-01 | chr2 | C | T | Missense_Mutation | p.R132H | 0.38 |
| TCGA-E1-A7YU-01 | chr2 | C | T | Missense_Mutation | p.R132H | 0.27 |
| TCGA-S9-A6WQ-01 | chr2 | C | T | Missense_Mutation | p.R132H | 0.31 |
| TCGA-HT-7475-01 | chr2 | C | T | Missense_Mutation | p.R132H | 0.31 |
| TCGA-DB-A64S-01 | chr2 | G | A | Missense_Mutation | p.R132C | 0.31 |
| TCGA-IK-8125-01 | chr2 | C | T | Missense_Mutation | p.R132H | 0.23 |
| TCGA-HT-7474-01 | chr2 | C | T | Missense_Mutation | p.R132H | 0.35 |
| TCGA-VV-A829-01 | chr2 | C | T | Missense_Mutation | p.R132H | 0.33 |
| TCGA-P5-A77W-01 | chr2 | C | T | Missense_Mutation | p.R132H | 0.42 |
| TCGA-FG-8187-01 | chr2 | C | T | Missense_Mutation | p.R132H | 0.27 |
| TCGA-DU-7306-01 | chr2 | C | T | Missense_Mutation | p.R132H | 0.34 |
| TCGA-HT-7483-01 | chr2 | G | C | Missense_Mutation | p.R132G | 0.33 |
| TCGA-DB-A4XE-01 | chr2 | C | T | Missense_Mutation | p.R132H | 0.52 |
| TCGA-QH-A6XA-01 | chr2 | C | T | Missense_Mutation | p.R132H | 0.38 |
| TCGA-DB-A64Q-01 | chr2 | C | T | Missense_Mutation | p.R132H | 0.4 |
| TCGA-FG-A4MW-01 | no variant | no variant | no variant | no variant | no variant | no variant |
| TCGA-WY-A85D-01 | chr2 | C | T | Missense_Mutation | p.R132H | 0.4 |
| TCGA-HT-7880-01 | chr2 | C | T | Missense_Mutation | p.R132H | 0.21 |
| TCGA-DU-5871-01 | chr2 | G | C | Missense_Mutation | p.R132G | 0.44 |
| TCGA-TQ-A7RG-01 | chr2 | C | T | Missense_Mutation | p.R132H | 0.39 |
| TCGA-DU-7019-01 | chr2 | C | T | Missense_Mutation | p.R132H | 0.4 |
| TCGA-DU-8161-01 | no variant | no variant | no variant | no variant | no variant | no variant |
| TCGA-DU-A7T8-01 | chr2 | C | T | Missense_Mutation | p.R132H | 0.31 |
| TCGA-DU-8167-01 | chr2 | C | T | Missense_Mutation | p.R132H | 0.37 |
| TCGA-TQ-A7RK-01 | chr2 | G | C | Missense_Mutation | p.R132G | 0.37 |
| TCGA-TM-A84L-01 | chr2 | C | T | Missense_Mutation | p.R132H | 0.28 |
| TCGA-QH-A870-01 | chr2 | C | T | Missense_Mutation | p.R132H | 0.35 |
| TCGA-FG-8188-01 | chr2 | C | T | Missense_Mutation | p.R132H | 0.37 |
| TCGA-DU-8162-01 | no variant | no variant | no variant | no variant | no variant | no variant |
| TCGA-DU-5852-01 | no variant | no variant | no variant | no variant | no variant | no variant |
| TCGA-HT-A74J-01 | chr2 | C | T | Missense_Mutation | p.R132H | 0.5 |
| TCGA-FG-A60J-01 | chr2 | C | T | Missense_Mutation | p.R132H | 0.23 |
| TCGA-DU-7011-01 | chr2 | C | T | Missense_Mutation | p.R132H | 0.25 |
| TCGA-DU-5872-02 |  |  |  |  |  |  |
| TCGA-HT-A614-01 | chr2 | C | T | Missense_Mutation | p.R132H | 0.28 |
| TCGA-FG-A4MY-01 | chr2 | C | T | Missense_Mutation | p.R132H | 0.25 |
| TCGA-HT-8013-01 | chr2 | C | T | Missense_Mutation | p.R132H | 0.47 |
| TCGA-E1-5322-01 | chr2 | C | T | Missense_Mutation | p.R132H | 0.32 |
| TCGA-FG-5965-01 | chr2 | C | T | Missense_Mutation | p.R132H | 0.26 |
| TCGA-HT-8114-01 | chr2 | C | T | Missense_Mutation | p.R132H | 0.47 |
| TCGA-WY-A85E-01 | chr2 | C | T | Missense_Mutation | p.R132H | 0.3 |
| TCGA-DU-5855-01 | chr2 | C | T | Missense_Mutation | p.R132H | 0.32 |
| TCGA-TQ-A7RO-01 | no variant | no variant | no variant | no variant | no variant | no variant |
| TCGA-HT-8111-01 | chr2 | C | T | Missense_Mutation | p.R132H | 0.4 |
| TCGA-DB-A64U-01 | chr2 | C | T | Missense_Mutation | p.R132H | 0.19 |
| TCGA-TQ-A7RJ-01 | chr2 | G | C | Missense_Mutation | p.R132G | 0.38 |
| TCGA-FG-A60K-01 | chr2 | C | T | Missense_Mutation | p.R132H | 0.46 |
| TCGA-TQ-A7RP-01 | no variant | no variant | no variant | no variant | no variant | no variant |
| TCGA-HT-A74L-01 | chr2 | C | T | Missense_Mutation | p.R132H | 0.33 |
| TCGA-HT-7610-01 | chr2 | C | T | Missense_Mutation | p.R132H | 0.28 |
| TCGA-E1-A7YV-01 | chr2 | C | T | Missense_Mutation | p.R132H | 0.28 |
| TCGA-HT-7611-01 | chr2 | G | C | Missense_Mutation | p.R132G | 0.32 |
| TCGA-TM-A84T-01 | chr2 | C | T | Missense_Mutation | p.R132H | 0.33 |
| TCGA-DB-5280-01 | chr2 | C | T | Missense_Mutation | p.R132H | 0.39 |
| TCGA-DB-5276-01 | chr2 | G | A | Missense_Mutation | p.R132C | 0.19 |
| TCGA-DU-5851-01 | chr2 | C | T | Missense_Mutation | p.R132H | 0.25 |
| TCGA-HT-7482-01 | chr2 | G | C | Missense_Mutation | p.R132G | 0.27 |
| TCGA-QH-A6X3-01 | chr2 | C | T | Missense_Mutation | p.R132H | 0.44 |
| TCGA-DB-A4XH-01 | chr2 | C | T | Missense_Mutation | p.R132H | 0.42 |
| TCGA-HT-7879-01 | chr2 | C | T | Missense_Mutation | p.R132H | 0.19 |
| TCGA-DU-5853-01 | chr2 | C | T | Missense_Mutation | p.R132H | 0.47 |
| TCGA-DB-A4XC-01 | chr2 | C | T | Missense_Mutation | p.R132H | 0.19 |
| TCGA-TQ-A7RH-01 | chr2 | C | T | Missense_Mutation | p.R132H | 0.41 |
| TCGA-TQ-A7RR-01 | chr2 | C | T | Missense_Mutation | p.R132H | 0.36 |
| TCGA-HT-7609-01 | chr2 | C | T | Missense_Mutation | p.R132H | 0.43 |
| TCGA-FN-7833-01 | chr2 | C | T | Missense_Mutation | p.R132H | 0.33 |
| TCGA-DU-6542-01 | chr2 | C | T | Missense_Mutation | p.R132H | 0.28 |
| TCGA-DB-5281-01 | chr2 | C | T | Missense_Mutation | p.R132H | 0.42 |
| TCGA-HT-7873-01 | chr2 | C | T | Missense_Mutation | p.R132H | 0.26 |
| TCGA-DU-8163-01 | chr2 | C | T | Missense_Mutation | p.R132H | 0.26 |
| TCGA-DU-8166-01 | chr2 | C | T | Missense_Mutation | p.R132H | 0.39 |
| TCGA-HT-7902-01 | chr2 | C | T | Missense_Mutation | p.R132H | 0.19 |
| TCGA-QH-A6CW-01 | chr2 | C | T | Missense_Mutation | p.R132H | 0.48 |
| TCGA-E1-A7YW-01 | chr2 | C | T | Missense_Mutation | p.R132H | 0.43 |
| TCGA-DH-5140-01 | no variant | no variant | no variant | no variant | no variant | no variant |
| TCGA-FG-A4MU-01 | no variant | no variant | no variant | no variant | no variant | no variant |
| TCGA-HT-7473-01 | chr2 | C | T | Missense_Mutation | p.R132H | 0.4 |
| TCGA-DU-6403-01 | no variant | no variant | no variant | no variant | no variant | no variant |
| TCGA-DU-6406-01 | no variant | no variant | no variant | no variant | no variant | no variant |
| TCGA-TQ-A7RM-01 | chr2 | C | T | Missense_Mutation | p.R132H | 0.48 |
| TCGA-DU-6396-01 | chr2 | C | T | Missense_Mutation | p.R132H | 0.29 |
| TCGA-DU-A7TD-01 | no variant | no variant | no variant | no variant | no variant | no variant |
| TCGA-TM-A84H-01 | chr2 | C | T | Missense_Mutation | p.R132H | 0.35 |
| TCGA-FG-A6IZ-01 | chr2 | C | T | Missense_Mutation | p.R132H | 0.35 |
| TCGA-FG-A6J1-01 | chr2 | C | T | Missense_Mutation | p.R132H | 0.45 |
| TCGA-HT-A74K-01 | chr2 | C | T | Missense_Mutation | p.R132H | 0.28 |
| TCGA-R8-A73M-01 | chr2 | C | T | Missense_Mutation | p.R132H | 0.45 |
| TCGA-P5-A5F6-01 | no variant | no variant | no variant | no variant | no variant | no variant |
| TCGA-RY-A840-01 | chr2 | C | T | Missense_Mutation | p.R132H | 0.31 |
| TCGA-S9-A6TW-01 | chr2 | C | T | Missense_Mutation | p.R132H | 0.38 |
| TCGA-RY-A83Y-01 | chr2 | C | T | Missense_Mutation | p.R132H | 0.39 |
| TCGA-VM-A8CA-01 | chr2 | C | T | Missense_Mutation | p.R132H | 0.36 |
| TCGA-VM-A8CB-01 | chr2 | C | T | Missense_Mutation | p.R132H | 0.36 |
| TCGA-HT-7603-01 | chr2 | C | T | Missense_Mutation | p.R132H | 0.27 |
| TCGA-DU-A6S8-01 | chr2 | C | T | Missense_Mutation | p.R132H | 0.44 |
| TCGA-DH-5141-01 | no variant | no variant | no variant | no variant | no variant | no variant |
| TCGA-QH-A6CU-01 | chr2 | C | T | Missense_Mutation | p.R132H | 0.29 |
| TCGA-HT-7693-01 | chr2 | G | A | Missense_Mutation | p.R132C | 0.47 |
| TCGA-DH-A7UR-01 | no variant | no variant | no variant | no variant | no variant | no variant |
| TCGA-DU-5874-01 | chr2 | C | T | Missense_Mutation | p.R132H | 0.34 |
| TCGA-DU-7018-01 | chr2 | C | T | Missense_Mutation | p.R132H | 0.48 |
| TCGA-S9-A6TY-01 | no variant | no variant | no variant | no variant | no variant | no variant |
| TCGA-QH-A65Z-01 | chr2 | C | T | Missense_Mutation | p.R132H | 0.38 |
| TCGA-HT-A4DV-01 | chr2 | C | T | Missense_Mutation | p.R132H | 0.16 |
| TCGA-VW-A7QS-01 | chr2 | C | T | Missense_Mutation | p.R132H | 0.38 |
| TCGA-DB-A64L-01 | chr2 | C | T | Missense_Mutation | p.R132H | 0.31 |
| TCGA-F6-A8O3-01 | chr2 | C | T | Missense_Mutation | p.R132H | 0.3 |
| TCGA-DU-A7T6-01 | chr2 | C | T | Missense_Mutation | p.R132H | 0.39 |
| TCGA-FG-5964-01 | chr2 | C | T | Missense_Mutation | p.R132H | 0.19 |
| TCGA-QH-A65V-01 | chr2 | C | T | Missense_Mutation | p.R132H | 0.33 |
| TCGA-VM-A8C8-01 | chr2 | C | T | Missense_Mutation | p.R132H | 0.43 |
| TCGA-QH-A65R-01 | chr2 | C | T | Missense_Mutation | p.R132H | 0.32 |
| TCGA-DU-6394-01 | chr2 | C | T | Missense_Mutation | p.R132H | 0.3 |
| TCGA-S9-A6WD-01 | chr2 | C | T | Missense_Mutation | p.R132H | 0.43 |
| TCGA-HT-A617-01 | no variant | no variant | no variant | no variant | no variant | no variant |
| TCGA-FG-A710-01 | chr2 | C | T | Missense_Mutation | p.R132H | 0.34 |
| TCGA-FG-5962-01 | chr2 | C | T | Missense_Mutation | p.R132H | 0.3 |
| TCGA-DU-A76R-01 | chr2 | C | T | Missense_Mutation | p.R132H | 0.37 |
| TCGA-HW-A5KJ-01 | chr2 | C | T | Missense_Mutation | p.R132H | 0.44 |
| TCGA-HT-7605-01 | chr2 | C | T | Missense_Mutation | p.R132H | 0.32 |
| TCGA-R8-A6MO-01 | chr2 | C | T | Missense_Mutation | p.R132H | 0.3 |
| TCGA-S9-A6TX-01 | chr2 | C | T | Missense_Mutation | p.R132H | 0.42 |
| TCGA-E1-A7YY-01 | chr2 | C | T | Missense_Mutation | p.R132H | 0.13 |
| TCGA-DB-A64V-01 | chr2 | C | T | Missense_Mutation | p.R132H | 0.28 |
| TCGA-DB-A64P-01 | chr2 | C | T | Missense_Mutation | p.R132H | 0.51 |
| TCGA-TM-A84R-01 | no variant | no variant | no variant | no variant | no variant | no variant |
| TCGA-S9-A6UB-01 | chr2 | C | T | Missense_Mutation | p.R132H | 0.41 |
| TCGA-P5-A5F4-01 | chr2 | G | A | Missense_Mutation | p.R132C | 0.3 |
| TCGA-DU-6393-01 | chr2 | C | T | Missense_Mutation | p.R132H | 0.34 |
| TCGA-DU-A76K-01 | no variant | no variant | no variant | no variant | no variant | no variant |
| TCGA-E1-5318-01 | no variant | no variant | no variant | no variant | no variant | no variant |
| TCGA-DU-6400-01 | chr2 | C | T | Missense_Mutation | p.R132H | 0.41 |
| TCGA-DH-5144-01 | chr2 | C | T | Missense_Mutation | p.R132H | 0.46 |
| TCGA-HT-A61C-01 | no variant | no variant | no variant | no variant | no variant | no variant |
| TCGA-P5-A5EX-01 | chr2 | C | T | Missense_Mutation | p.R132H | 0.23 |
| TCGA-E1-A7Z2-01 | no variant | no variant | no variant | no variant | no variant | no variant |
| TCGA-HT-7471-01 | chr2 | C | T | Missense_Mutation | p.R132H | 0.43 |
| TCGA-TQ-A7RU-01 | chr2 | C | T | Missense_Mutation | p.R132H | 0.43 |
| TCGA-DU-A6S3-01 | chr2 | C | T | Missense_Mutation | p.R132H | 0.34 |
| TCGA-R8-A6ML-01 | chr2 | C | T | Missense_Mutation | p.R132H | 0.41 |
| TCGA-TQ-A7RF-01 | chr2 | C | T | Missense_Mutation | p.R132H | 0.42 |
| TCGA-P5-A5ET-01 | chr2 | C | T | Missense_Mutation | p.R132H | 0.35 |
| TCGA-FG-7638-01 | chr2 | C | T | Missense_Mutation | p.R132H | 0.35 |
| TCGA-TM-A84S-01 | chr2 | C | T | Missense_Mutation | p.R132H | 0.38 |
| TCGA-TQ-A7RN-01 | chr2 | C | T | Missense_Mutation | p.R132H | 0.4 |
| TCGA-HT-7694-01 | chr2 | C | T | Missense_Mutation | p.R132H | 0.23 |
| TCGA-E1-5319-01 | chr2 | C | T | Missense_Mutation | p.R132H | 0.37 |
| TCGA-FG-A4MT-01 | chr2 | C | T | Missense_Mutation | p.R132H | 0.55 |
| TCGA-QH-A6X8-01 | chr2 | C | T | Missense_Mutation | p.R132H | 0.34 |
| TCGA-TQ-A7RS-01 | chr2 | C | T | Missense_Mutation | p.R132H | 0.44 |
| TCGA-TM-A84G-01 | chr2 | C | T | Missense_Mutation | p.R132H | 0.54 |
| TCGA-HT-7616-01 | chr2 | C | T | Missense_Mutation | p.R132H | 0.41 |
| TCGA-DU-7008-01 | chr2 | C | T | Missense_Mutation | p.R132H | 0.33 |
| TCGA-RY-A847-01 | chr2 | C | T | Missense_Mutation | p.R132H | 0.4 |
| TCGA-EZ-7264-01 | chr2 | C | T | Missense_Mutation | p.R132H | 0.37 |
| TCGA-S9-A6WE-01 | chr2 | C | T | Missense_Mutation | p.R132H | 0.41 |
| TCGA-FG-A711-01 | chr2 | C | T | Missense_Mutation | p.R132H | 0.31 |
| TCGA-HT-7676-01 | chr2 | C | T | Missense_Mutation | p.R132H | 0.39 |
| TCGA-CS-6670-01 | chr2 | C | T | Missense_Mutation | p.R132H | 0.41 |
| TCGA-FG-6692-01 | no variant | no variant | no variant | no variant | no variant | no variant |
| TCGA-DH-A66F-01 | chr2 | C | T | Missense_Mutation | p.R132H | 0.39 |
| TCGA-DB-5279-01 | chr2 | C | T | Missense_Mutation | p.R132H | 0.47 |
| TCGA-HW-7487-01 | chr2 | C | T | Missense_Mutation | p.R132H | 0.21 |
| TCGA-HT-8113-01 | no variant | no variant | no variant | no variant | no variant | no variant |
| TCGA-R8-A6MK-01 | chr2 | C | T | Missense_Mutation | p.R132H | 0.34 |
| TCGA-DU-5849-01 | no variant | no variant | no variant | no variant | no variant | no variant |
| TCGA-W9-A837-01 | chr2 | C | T | Missense_Mutation | p.R132H | 0.4 |
| TCGA-HT-7688-01 | chr2 | C | T | Missense_Mutation | p.R132H | 0.27 |
| TCGA-RY-A83X-01 | chr2 | C | T | Missense_Mutation | p.R132H | 0.36 |
| TCGA-DU-7309-01 | no variant | no variant | no variant | no variant | no variant | no variant |
| TCGA-DU-6397-01 | chr2 | C | T | Missense_Mutation | p.R132H | 0.39 |
| TCGA-HT-A615-01 | chr2 | C | T | Missense_Mutation | p.R132H | 0.34 |
| TCGA-HT-7481-01 | chr2 | C | T | Missense_Mutation | p.R132H | 0.18 |
| TCGA-DU-5870-01 | chr2 | C | T | Missense_Mutation | p.R132H | 0.36 |
| TCGA-DU-A5TS-01 | chr2 | C | T | Missense_Mutation | p.R132H | 0.31 |
| TCGA-HT-8105-01 | chr2 | C | T | Missense_Mutation | p.R132H | 0.41 |
| TCGA-FG-8189-01 | no variant | no variant | no variant | no variant | no variant | no variant |
| TCGA-DU-8164-01 | chr2 | C | T | Missense_Mutation | p.R132H | 0.34 |
| TCGA-HT-8010-01 | chr2 | C | T | Missense_Mutation | p.R132H | 0.29 |
| TCGA-P5-A72Z-01 | chr2 | C | T | Missense_Mutation | p.R132H | 0.59 |
| TCGA-P5-A5F0-01 | chr2 | C | T | Missense_Mutation | p.R132H | 0.29 |
| TCGA-HT-A61A-01 | no variant | no variant | no variant | no variant | no variant | no variant |
| TCGA-DB-5278-01 | chr2 | C | T | Missense_Mutation | p.R132H | 0.39 |
| TCGA-TQ-A8XE-01 | chr2 | C | T | Missense_Mutation | p.R132H | 0.32 |
| TCGA-HW-7491-01 | no variant | no variant | no variant | no variant | no variant | no variant |
| TCGA-HT-A5R9-01 | no variant | no variant | no variant | no variant | no variant | no variant |
| TCGA-CS-5396-01 | chr2 | C | T | Missense_Mutation | p.R132H | 0.44 |
| TCGA-DU-7294-01 | chr2 | C | T | Missense_Mutation | p.R132H | 0.43 |
| TCGA-DB-A4XG-01 | no variant | no variant | no variant | no variant | no variant | no variant |
| TCGA-S9-A7J0-01 | chr2 | G | T | Missense_Mutation | p.R132S | 0.58 |
| TCGA-DU-7009-01 | chr2 | C | T | Missense_Mutation | p.R132H | 0.44 |
| TCGA-DU-A5TT-01 | no variant | no variant | no variant | no variant | no variant | no variant |
| TCGA-S9-A7R1-01 | chr2 | C | T | Missense_Mutation | p.R132H | 0.4 |
| TCGA-HW-7486-01 | chr2 | C | T | Missense_Mutation | p.R132H | 0.14 |
| TCGA-HT-7695-01 | chr2 | C | T | Missense_Mutation | p.R132H | 0.32 |
| TCGA-DU-A7TG-01 | chr2 | C | T | Missense_Mutation | p.R132H | 0.09 |
| TCGA-DH-A669-01 | chr2 | C | T | Missense_Mutation | p.R132H | 0.44 |
| TCGA-DH-A7US-01 | chr2 | C | T | Missense_Mutation | p.R132H | 0.34 |
| TCGA-DU-7302-01 | chr2 | C | T | Missense_Mutation | p.R132H | 0.37 |
| TCGA-HT-7856-01 | no variant | no variant | no variant | no variant | no variant | no variant |
| TCGA-HT-8558-01 | no variant | no variant | no variant | no variant | no variant | no variant |
| TCGA-TM-A84O-01 | chr2 | C | T | Missense_Mutation | p.R132H | 0.36 |
| TCGA-HT-7874-01 | chr2 | C | T | Missense_Mutation | p.R132H | 0.16 |
| TCGA-DU-6399-01 | chr2 | C | T | Missense_Mutation | p.R132H | 0.43 |
| TCGA-HT-7687-01 | chr2 | C | T | Missense_Mutation | p.R132H | 0.42 |
| TCGA-IK-7675-01 | chr2 | C | T | Missense_Mutation | p.R132H | 0.51 |
| TCGA-HT-7602-01 | chr2 | C | T | Missense_Mutation | p.R132H | 0.21 |
| TCGA-P5-A72U-01 | no variant | no variant | no variant | no variant | no variant | no variant |
| TCGA-DB-A64R-01 | chr2 | C | T | Missense_Mutation | p.R132H | 0.4 |
| TCGA-DU-7300-01 | chr2 | C | T | Missense_Mutation | p.R132H | 0.2 |
| TCGA-HT-7677-01 | chr2 | C | T | Missense_Mutation | p.R132H | 0.42 |
| TCGA-HT-A5R5-01 | chr2 | C | T | Missense_Mutation | p.R132H | 0.3 |
| TCGA-HT-8012-01 | chr2 | C | T | Missense_Mutation | p.R132H | 0.44 |
| TCGA-S9-A7QZ-01 | chr2 | C | T | Missense_Mutation | p.R132H | 0.38 |
| TCGA-HT-8107-01 | no variant | no variant | no variant | no variant | no variant | no variant |
| TCGA-DU-6410-01 | chr2 | C | T | Missense_Mutation | p.R132H | 0.39 |
| TCGA-HT-7881-01 | chr2 | C | T | Missense_Mutation | p.R132H | 0.12 |
| TCGA-HT-8109-01 | chr2 | C | T | Missense_Mutation | p.R132H | 0.27 |
| TCGA-QH-A86X-01 | chr2 | C | T | Missense_Mutation | p.R132H | 0.39 |
| TCGA-S9-A7J2-01 | chr2 | C | T | Missense_Mutation | p.R132H | 0.45 |
| TCGA-HT-7875-01 | chr2 | C | T | Missense_Mutation | p.R132H | 0.34 |
| TCGA-DU-6408-01 | chr2 | C | T | Missense_Mutation | p.R132H | 0.36 |
| TCGA-TM-A84M-01 | chr2 | C | T | Missense_Mutation | p.R132H | 0.49 |
| TCGA-HT-7467-01 | chr2 | C | T | Missense_Mutation | p.R132H | 0.12 |
| TCGA-VM-A8CE-01 | chr2 | C | T | Missense_Mutation | p.R132H | 0.26 |
| TCGA-DU-A7TA-01 | no variant | no variant | no variant | no variant | no variant | no variant |
| TCGA-HW-7495-01 | chr2 | C | T | Missense_Mutation | p.R132H | 0.32 |
| TCGA-DU-7301-01 | chr2 | C | T | Missense_Mutation | p.R132H | 0.33 |
| TCGA-HT-7689-01 | chr2 | C | T | Missense_Mutation | p.R132H | 0.28 |
| TCGA-DU-6401-01 | chr2 | G | T | Missense_Mutation | p.R132S | 0.35 |
| TCGA-HT-7877-01 | chr2 | C | T | Missense_Mutation | p.R132H | 0.45 |
| TCGA-DU-8168-01 | chr2 | C | T | Missense_Mutation | p.R132H | 0.45 |
| TCGA-HT-7468-01 | chr2 | C | T | Missense_Mutation | p.R132H | 0.36 |
| TCGA-FG-A70Y-01 | chr2 | C | T | Missense_Mutation | p.R132H | 0.26 |
| TCGA-S9-A7J3-01 | chr2 | C | T | Missense_Mutation | p.R132H | 0.29 |
| TCGA-QH-A6X9-01 | chr2 | C | T | Missense_Mutation | p.R132H | 0.4 |
| TCGA-HT-7480-01 | chr2 | C | T | Missense_Mutation | p.R132H | 0.42 |
| TCGA-S9-A6U2-01 | chr2 | C | T | Missense_Mutation | p.R132H | 0.32 |
| TCGA-E1-5311-01 | chr2 | C | T | Missense_Mutation | p.R132H | 0.44 |
| TCGA-HT-7620-01 | chr2 | C | T | Missense_Mutation | p.R132H | 0.19 |
| TCGA-TQ-A7RI-01 | chr2 | C | T | Missense_Mutation | p.R132H | 0.36 |
| TCGA-S9-A7J1-01 | chr2 | C | T | Missense_Mutation | p.R132H | 0.37 |
| TCGA-DU-A6S2-01 | chr2 | C | T | Missense_Mutation | p.R132H | 0.41 |
| TCGA-DU-6407-01 | chr2 | C | T | Missense_Mutation | p.R132H | 0.38 |
| TCGA-FG-7641-01 | chr2 | C | T | Missense_Mutation | p.R132H | 0.27 |
| TCGA-TQ-A7RQ-01 | chr2 | C | T | Missense_Mutation | p.R132H | 0.45 |
| TCGA-HT-7470-01 | chr2 | G | T | Missense_Mutation | p.R132S | 0.25 |
| TCGA-E1-A7YQ-01 | no variant | no variant | no variant | no variant | no variant | no variant |
| TCGA-HW-8322-01 | chr2 | C | T | Missense_Mutation | p.R132H | 0.38 |
| TCGA-FG-7634-01 | chr2 | C | T | Missense_Mutation | p.R132H | 0.41 |
| TCGA-CS-5390-01 | chr2 | C | T | Missense_Mutation | p.R132H | 0.34 |
| TCGA-E1-A7YO-01 | chr2 | C | T | Missense_Mutation | p.R132H | 0.44 |
| TCGA-HT-A619-01 | chr2 | C | T | Missense_Mutation | p.R132H | 0.41 |
| TCGA-CS-6668-01 | chr2 | C | T | Missense_Mutation | p.R132H | 0.46 |
| TCGA-DU-8165-01 | no variant | no variant | no variant | no variant | no variant | no variant |
| TCGA-CS-5395-01 | no variant | no variant | no variant | no variant | no variant | no variant |
| TCGA-DU-6404-01 | no variant | no variant | no variant | no variant | no variant | no variant |
| TCGA-DH-A66G-01 | chr2 | C | T | Missense_Mutation | p.R132H | 0.38 |
| TCGA-HT-7469-01 | no variant | no variant | no variant | no variant | no variant | no variant |
| TCGA-FG-6690-01 | chr2 | C | T | Missense_Mutation | p.R132H | 0.26 |
| TCGA-DU-7015-01 | chr2 | C | T | Missense_Mutation | p.R132H | 0.38 |
| TCGA-HT-8108-01 | chr2 | C | T | Missense_Mutation | p.R132H | 0.4 |
| TCGA-DU-A76L-01 | no variant | no variant | no variant | no variant | no variant | no variant |
| TCGA-E1-A7YS-01 | chr2 | C | T | Missense_Mutation | p.R132H | 0.53 |
| TCGA-HT-7472-01 | chr2 | C | T | Missense_Mutation | p.R132H | 0.38 |
| TCGA-FG-8182-01 | chr2 | C | T | Missense_Mutation | p.R132H | 0.34 |
| TCGA-HT-7882-01 | no variant | no variant | no variant | no variant | no variant | no variant |
| TCGA-FG-8191-01 | chr2 | C | T | Missense_Mutation | p.R132H | 0.35 |
| TCGA-TQ-A7RW-01 | chr2 | C | T | Missense_Mutation | p.R132H | 0.36 |
| TCGA-DU-A7TB-01 | no variant | no variant | no variant | no variant | no variant | no variant |
| TCGA-TM-A84J-01 | no variant | no variant | no variant | no variant | no variant | no variant |
| TCGA-FG-A60L-01 | chr2 | C | T | Missense_Mutation | p.R132H | 0.35 |
| TCGA-RY-A83Z-01 | chr2 | C | T | Missense_Mutation | p.R132H | 0.35 |
| TCGA-HT-A4DS-01 | no variant | no variant | no variant | no variant | no variant | no variant |
| TCGA-FG-A6J3-01 | chr2 | C | T | Missense_Mutation | p.R132H | 0.21 |
| TCGA-VM-A8C9-01 | no variant | no variant | no variant | no variant | no variant | no variant |
| TCGA-VM-A8CF-01 | chr2 | C | T | Missense_Mutation | p.R132H | 0.45 |
| TCGA-DU-7290-01 | no variant | no variant | no variant | no variant | no variant | no variant |
| TCGA-HW-7490-01 | chr2 | C | T | Missense_Mutation | p.R132H | 0.27 |
| TCGA-P5-A72W-01 | chr2 | C | T | Missense_Mutation | p.R132H | 0.53 |
| TCGA-WY-A859-01 | chr2 | G | T | Missense_Mutation | p.R132S | 0.38 |
| TCGA-DU-A7TC-01 | chr2 | C | T | Missense_Mutation | p.R132H | 0.3 |
| TCGA-HT-A74O-01 | chr2 | G | C | Missense_Mutation | p.R132G | 0.48 |
| TCGA-P5-A5F1-01 | chr2 | C | T | Missense_Mutation | p.R132H | 0.32 |
| TCGA-QH-A6XC-01 | no variant | no variant | no variant | no variant | no variant | no variant |
| TCGA-CS-4944-01 | chr2 | C | T | Missense_Mutation | p.R132H | 0.3 |
| TCGA-HT-7606-01 | chr2 | C | T | Missense_Mutation | p.R132H | 0.38 |
| TCGA-DB-A75P-01 | no variant | no variant | no variant | no variant | no variant | no variant |
| TCGA-P5-A735-01 | chr2 | C | T | Missense_Mutation | p.R132H | 0.51 |
| TCGA-HT-A74H-01 | no variant | no variant | no variant | no variant | no variant | no variant |
| TCGA-DB-A75L-01 | chr2 | C | T | Missense_Mutation | p.R132H | 0.36 |
| TCGA-DU-A76O-01 | chr2 | C | T | Missense_Mutation | p.R132H | 0.36 |
| TCGA-P5-A5EV-01 | chr2 | C | T | Missense_Mutation | p.R132H | 0.4 |
| TCGA-S9-A6U5-01 | chr2 | C | T | Missense_Mutation | p.R132H | 0.37 |
| TCGA-HT-7476-01 | no variant | no variant | no variant | no variant | no variant | no variant |
| TCGA-FG-A4MX-01 | chr2 | C | T | Missense_Mutation | p.R132H | 0.44 |
| TCGA-DU-A5TU-01 | chr2 | C | T | Missense_Mutation | p.R132H | 0.37 |
| TCGA-DB-A75O-01 | chr2 | C | T | Missense_Mutation | p.R132H | 0.35 |
| TCGA-DB-A64X-01 | chr2 | C | T | Missense_Mutation | p.R132H | 0.38 |
| TCGA-FG-7636-01 | chr2 | C | T | Missense_Mutation | p.R132H | 0.41 |
| TCGA-S9-A6TS-01 | chr2 | C | T | Missense_Mutation | p.R132H | 0.45 |
| TCGA-F6-A8O4-01 | chr2 | C | T | Missense_Mutation | p.R132H | 0.29 |
| TCGA-S9-A6U6-01 | chr2 | C | T | Missense_Mutation | p.R132H | 0.35 |
| TCGA-FG-8185-01 | chr2 | G | A | Missense_Mutation | p.R132C | 0.37 |
| TCGA-WH-A86K-01 | chr2 | C | T | Missense_Mutation | p.R132H | 0.4 |
| TCGA-P5-A736-01 | chr2 | C | T | Missense_Mutation | p.R132H | 0.38 |
| TCGA-HT-7691-01 | no variant | no variant | no variant | no variant | no variant | no variant |
| TCGA-HT-A5R7-01 | chr2 | C | T | Missense_Mutation | p.R132H | 0.19 |
| TCGA-DB-A75M-01 | chr2 | G | A | Missense_Mutation | p.R132C | 0.2 |
| TCGA-P5-A733-01 | chr2 | C | T | Missense_Mutation | p.R132H | 0.39 |
| TCGA-DU-A5TW-01 | chr2 | C | T | Missense_Mutation | p.R132H | 0.5 |
| TCGA-P5-A781-01 | chr2 | C | T | Missense_Mutation | p.R132H | 0.27 |
| TCGA-S9-A7QW-01 | chr2 | C | T | Missense_Mutation | p.R132H | 0.42 |
| TCGA-QH-A6CX-01 | no variant | no variant | no variant | no variant | no variant | no variant |
| TCGA-CS-5394-01 | chr2 | C | T | Missense_Mutation | p.R132H | 0.33 |
| TCGA-S9-A89Z-01 | chr2 | C | T | Missense_Mutation | p.R132H | 0.28 |
| TCGA-VM-A8CD-01 | no variant | no variant | no variant | no variant | no variant | no variant |
| TCGA-HT-7607-01 | chr2 | C | T | Missense_Mutation | p.R132H | 0.14 |
| TCGA-WY-A85A-01 | chr2 | C | T | Missense_Mutation | p.R132H | 0.32 |
| TCGA-DU-7292-01 | no variant | no variant | no variant | no variant | no variant | no variant |
| TCGA-DH-5142-01 | chr2 | C | T | Missense_Mutation | p.R132H | 0.37 |
| TCGA-P5-A5EY-01 | no variant | no variant | no variant | no variant | no variant | no variant |
| TCGA-DU-5847-01 | no variant | no variant | no variant | no variant | no variant | no variant |
| TCGA-P5-A5F2-01 | chr2 | C | T | Missense_Mutation | p.R132H | 0.38 |
| TCGA-TM-A7C4-01 | chr2 | C | T | Missense_Mutation | p.R132H | 0.37 |
| TCGA-CS-5397-01 | no variant | no variant | no variant | no variant | no variant | no variant |
| TCGA-FG-6688-01 | no variant | no variant | no variant | no variant | no variant | no variant |
| TCGA-E1-A7Z3-01 | chr2 | C | T | Missense_Mutation | p.R132H | 0.23 |
| TCGA-DH-A66B-01 | chr2 | C | T | Missense_Mutation | p.R132H | 0.46 |
| TCGA-S9-A7R2-01 | no variant | no variant | no variant | no variant | no variant | no variant |
| TCGA-CS-6667-01 | chr2 | C | T | Missense_Mutation | p.R132H | 0.39 |
| TCGA-HT-A616-01 | chr2 | C | T | Missense_Mutation | p.R132H | 0.23 |
| TCGA-DU-7007-01 | chr2 | C | T | Missense_Mutation | p.R132H | 0.41 |
| TCGA-S9-A7IZ-01 | chr2 | C | T | Missense_Mutation | p.R132H | 0.33 |
| TCGA-FG-A87Q-01 | no variant | no variant | no variant | no variant | no variant | no variant |
| TCGA-HT-8015-01 | no variant | no variant | no variant | no variant | no variant | no variant |
| TCGA-S9-A7IX-01 | no variant | no variant | no variant | no variant | no variant | no variant |
| TCGA-CS-6666-01 | chr2 | G | A | Missense_Mutation | p.R132C | 0.4 |
| TCGA-WY-A858-01 | chr2 | C | T | Missense_Mutation | p.R132H | 0.28 |
| TCGA-S9-A7R3-01 | chr2 | C | T | Missense_Mutation | p.R132H | 0.41 |
| TCGA-S9-A6U0-01 | no variant | no variant | no variant | no variant | no variant | no variant |
| TCGA-FG-6689-01 | chr2 | C | T | Missense_Mutation | p.R132H | 0.3 |
| TCGA-P5-A72X-01 | chr2 | C | T | Missense_Mutation | p.R132H | 0.36 |
| TCGA-E1-5305-01 | chr2 | C | T | Missense_Mutation | p.R132H | 0.42 |
| TCGA-CS-4942-01 | chr2 | C | T | Missense_Mutation | p.R132H | 0.32 |
| TCGA-S9-A7QX-01 | chr2 | C | T | Missense_Mutation | p.R132H | 0.34 |
| TCGA-DU-7299-01 | chr2 | C | T | Missense_Mutation | p.R132H | 0.45 |
| TCGA-HT-8104-01 | no variant | no variant | no variant | no variant | no variant | no variant |
| TCGA-S9-A6WL-01 | chr2 | C | T | Missense_Mutation | p.R132H | 0.38 |
| TCGA-CS-4943-01 | chr2 | C | T | Missense_Mutation | p.R132H | 0.46 |
| TCGA-E1-5302-01 | chr2 | C | T | Missense_Mutation | p.R132H | 0.33 |
| TCGA-DU-6402-01 | no variant | no variant | no variant | no variant | no variant | no variant |
| TCGA-E1-A7YK-01 | chr2 | C | T | Missense_Mutation | p.R132H | 0.13 |
| TCGA-DU-6392-01 | no variant | no variant | no variant | no variant | no variant | no variant |
| TCGA-S9-A7IS-01 | chr2 | C | T | Missense_Mutation | p.R132H | 0.39 |
| TCGA-CS-4938-01 | chr2 | C | T | Missense_Mutation | p.R132H | 0.36 |
| TCGA-P5-A5EU-01 | chr2 | C | T | Missense_Mutation | p.R132H | 0.38 |
| TCGA-TM-A84Q-01 | chr2 | C | T | Missense_Mutation | p.R132H | 0.51 |
| TCGA-HT-7604-01 | chr2 | G | T | Missense_Mutation | p.R132S | 0.54 |
| TCGA-HT-8011-01 | no variant | no variant | no variant | no variant | no variant | no variant |
| TCGA-E1-A7YM-01 | no variant | no variant | no variant | no variant | no variant | no variant |
| TCGA-TM-A84F-01 | chr2 | C | T | Missense_Mutation | p.R132H | 0.22 |
| TCGA-E1-5304-01 | chr2 | C | T | Missense_Mutation | p.R132H | 0.56 |
| TCGA-DB-5273-01 | chr2 | C | T | Missense_Mutation | p.R132H | 0.26 |
| TCGA-E1-5307-01 | chr2 | C | T | Missense_Mutation | p.R132H | 0.27 |
| TCGA-DU-A6S7-01 | chr2 | G | T | Missense_Mutation | p.R132S | 0.45 |
| TCGA-E1-A7YN-01 | no variant | no variant | no variant | no variant | no variant | no variant |
| TCGA-TM-A84B-01 | no variant | no variant | no variant | no variant | no variant | no variant |
| TCGA-HT-8564-01 | no variant | no variant | no variant | no variant | no variant | no variant |
| TCGA-DH-A7UV-01 | chr2 | G | C | Missense_Mutation | p.R132G | 0.36 |
| TCGA-WY-A85B-01 | chr2 | G | C | Missense_Mutation | p.R132G | 0.37 |
| TCGA-HT-7485-01 | chr2 | C | T | Missense_Mutation | p.R132H | 0.33 |
| TCGA-HT-7478-01 | chr2 | C | T | Missense_Mutation | p.R132H | 0.33 |
| TCGA-E1-A7YJ-01 | no variant | no variant | no variant | no variant | no variant | no variant |
| TCGA-HT-7479-01 | chr2 | G | A | Missense_Mutation | p.R132C | 0.25 |
| TCGA-E1-A7Z4-01 | chr2 | C | T | Missense_Mutation | p.R132H | 0.4 |
| TCGA-RY-A843-01 | chr2 | C | T | Missense_Mutation | p.R132H | 0.27 |
| TCGA-HW-8319-01 | chr2 | C | T | Missense_Mutation | p.R132H | 0.37 |
| TCGA-VV-A86M-01 | chr2 | C | T | Missense_Mutation | p.R132H | 0.53 |
| TCGA-HT-7477-01 | chr2 | C | T | Missense_Mutation | p.R132H | 0.52 |
| TCGA-TQ-A7RV-01 | chr2 | C | T | Missense_Mutation | p.R132H | 0.39 |
| TCGA-S9-A6TU-01 | chr2 | C | T | Missense_Mutation | p.R132H | 0.33 |
| TCGA-HT-A5RA-01 | no variant | no variant | no variant | no variant | no variant | no variant |
| TCGA-DU-7298-01 | chr2 | C | T | Missense_Mutation | p.R132H | 0.29 |
| TCGA-VM-A8CH-01 | chr2 | G | A | Missense_Mutation | p.R132C | 0.41 |
| TCGA-S9-A6U1-01 | chr2 | G | A | Missense_Mutation | p.R132C | 0.34 |
| TCGA-DH-A7UT-01 | chr2 | C | T | Missense_Mutation | p.R132H | 0.36 |
| TCGA-TM-A7CA-01 | chr2 | C | T | Missense_Mutation | p.R132H | 0.34 |
| TCGA-WY-A85C-01 | chr2 | G | C | Missense_Mutation | p.R132G | 0.48 |
| TCGA-FG-5963-01 | no variant | no variant | no variant | no variant | no variant | no variant |
| TCGA-HT-7686-01 | chr2 | C | T | Missense_Mutation | p.R132H | 0.38 |
| TCGA-HT-7680-01 | no variant | no variant | no variant | no variant | no variant | no variant |
| TCGA-HT-A618-01 | no variant | no variant | no variant | no variant | no variant | no variant |
| TCGA-VW-A8FI-01 | no variant | no variant | no variant | no variant | no variant | no variant |
| TCGA-S9-A6U9-01 | chr2 | G | A | Missense_Mutation | p.R132C | 0.32 |
| TCGA-E1-A7YD-01 | no variant | no variant | no variant | no variant | no variant | no variant |
| TCGA-CS-6290-01 | chr2 | C | T | Missense_Mutation | p.R132H | 0.2 |
| TCGA-FG-6691-01 | chr2 | G | T | Missense_Mutation | p.R132S | 0.34 |
| TCGA-S9-A7R7-01 | chr2 | C | T | Missense_Mutation | p.R132H | 0.24 |
| TCGA-CS-6188-01 | no variant | no variant | no variant | no variant | no variant | no variant |
| TCGA-DU-6405-01 | no variant | no variant | no variant | no variant | no variant | no variant |
| TCGA-S9-A6U8-01 | chr2 | G | A | Missense_Mutation | p.R132C | 0.4 |
| TCGA-E1-A7Z6-01 | chr2 | C | T | Missense_Mutation | p.R132H | 0.33 |
| TCGA-DU-8158-01 | no variant | no variant | no variant | no variant | no variant | no variant |
| TCGA-DU-A5TY-01 | no variant | no variant | no variant | no variant | no variant | no variant |
| TCGA-E1-A7YL-01 | no variant | no variant | no variant | no variant | no variant | no variant |
| TCGA-CS-4941-01 | no variant | no variant | no variant | no variant | no variant | no variant |
| TCGA-DU-A7TJ-01 | no variant | no variant | no variant | no variant | no variant | no variant |
| TCGA-TM-A7C3-01 | no variant | no variant | no variant | no variant | no variant | no variant |
| TCGA-DU-A5TP-01 | chr2 | C | T | Missense_Mutation | p.R132H | 0.29 |
| TCGA-HW-8321-01 | chr2 | C | T | Missense_Mutation | p.R132H | 0.31 |
| TCGA-TM-A7CF-01 | chr2 | C | T | Missense_Mutation | p.R132H | 0.27 |
| TCGA-HT-7601-01 | chr2 | C | T | Missense_Mutation | p.R132H | 0.26 |
| TCGA-DB-A4XF-01 | chr2 | G | A | Missense_Mutation | p.R132C | 0.4 |
| TCGA-E1-A7YI-01 | chr2 | C | T | Missense_Mutation | p.R132H | 0.36 |
| TCGA-HT-7855-01 | chr2 | G | A | Missense_Mutation | p.R132C | 0.47 |
| TCGA-TM-A84C-01 | no variant | no variant | no variant | no variant | no variant | no variant |
| TCGA-S9-A6WN-01 | chr2 | C | T | Missense_Mutation | p.R132H | 0.21 |
| TCGA-HT-8110-01 | no variant | no variant | no variant | no variant | no variant | no variant |
| TCGA-E1-A7YH-01 | chr2 | C | T | Missense_Mutation | p.R132H | 0.32 |
| TCGA-HT-8563-01 | chr2 | C | T | Missense_Mutation | p.R132H | 0.22 |
| TCGA-E1-5303-01 | chr2 | C | T | Missense_Mutation | p.R132H | 0.31 |
| TCGA-HT-7860-01 | no variant | no variant | no variant | no variant | no variant | no variant |
| TCGA-HT-A61B-01 | chr2 | C | T | Missense_Mutation | p.R132H | 0.26 |
| TCGA-P5-A5EW-01 | chr2 | C | T | Missense_Mutation | p.R132H | 0.45 |
| TCGA-HT-7857-01 | no variant | no variant | no variant | no variant | no variant | no variant |
| TCGA-S9-A6WG-01 | chr2 | C | T | Missense_Mutation | p.R132H | 0.28 |
| TCGA-S9-A7R4-01 | chr2 | C | T | Missense_Mutation | p.R132H | 0.3 |
| TCGA-DU-5854-01 | no variant | no variant | no variant | no variant | no variant | no variant |
| TCGA-S9-A6TZ-01 | chr2 | C | T | Missense_Mutation | p.R132H | 0.36 |
| TCGA-DB-5277-01 | chr2 | C | T | Missense_Mutation | p.R132H | 0.27 |
| TCGA-P5-A5EZ-01 | chr2 | G | A | Missense_Mutation | p.R132C | 0.25 |
| TCGA-S9-A7R8-01 | chr2 | C | T | Missense_Mutation | p.R132H | 0.36 |
| TCGA-DB-A4XB-01 | chr2 | C | T | Missense_Mutation | p.R132H | 0.29 |
| TCGA-HT-A5RB-01 | chr2 | C | T | Missense_Mutation | p.R132H | 0.28 |
| TCGA-HW-A5KK-01 | no variant | no variant | no variant | no variant | no variant | no variant |
| TCGA-HW-8320-01 | chr2 | C | T | Missense_Mutation | p.R132H | 0.41 |
| TCGA-HW-A5KM-01 | chr2 | C | T | Missense_Mutation | p.R132H | 0.35 |
| TCGA-HT-7884-01 | chr2 | C | T | Missense_Mutation | p.R132H | 0.43 |
| TCGA-E1-A7YE-01 | chr2 | C | T | Missense_Mutation | p.R132H | 0.42 |
| TCGA-CS-5393-01 | chr2 | C | T | Missense_Mutation | p.R132H | 0.31 |
| TCGA-HT-7858-01 | chr2 | G | T | Missense_Mutation | p.R132S | 0.21 |
| TCGA-HT-7854-01 | no variant | no variant | no variant | no variant | no variant | no variant |
| TCGA-DH-A66D-01 | chr2 | C | T | Missense_Mutation | p.R132H | 0.36 |
| TCGA-S9-A6WO-01 | chr2 | C | T | Missense_Mutation | p.R132H | 0.44 |
| TCGA-QH-A6CS-01 | no variant | no variant | no variant | no variant | no variant | no variant |
| TCGA-DH-A7UU-01 | chr2 | C | T | Missense_Mutation | p.R132H | 0.45 |
| TCGA-HT-A5RC-01 | no variant | no variant | no variant | no variant | no variant | no variant |
| TCGA-HT-8106-01 | chr2 | C | T | Missense_Mutation | p.R132H | 0.17 |
| TCGA-S9-A6UA-01 | no variant | no variant | no variant | no variant | no variant | no variant |
| TCGA-S9-A6WM-01 | no variant | no variant | no variant | no variant | no variant | no variant |
| TCGA-KT-A7W1-01 | no variant | no variant | no variant | no variant | no variant | no variant |
| TCGA-P5-A780-01 | chr2 | C | T | Missense_Mutation | p.R132H | 0.33 |
| TCGA-S9-A89V-01 | no variant | no variant | no variant | no variant | no variant | no variant |
| TCGA-CS-6665-01 | chr2 | C | T | Missense_Mutation | p.R132H | 0.19 |
| TCGA-DU-7012-01 | no variant | no variant | no variant | no variant | no variant | no variant |
| TCGA-FG-A87N-01 | chr2 | C | T | Missense_Mutation | p.R132H | 0.34 |
| TCGA-DU-7010-01 | chr2 | C | T | Missense_Mutation | p.R132H | 0.33 |
| TCGA-DU-7013-01 | no variant | no variant | no variant | no variant | no variant | no variant |
| TCGA-HW-A5KL-01 | chr2 | C | T | Missense_Mutation | p.R132H | 0.3 |
| TCGA-DB-A4XD-01 | chr2 | G | A | Missense_Mutation | p.R132C | 0.4 |
| TCGA-DU-7006-01 | no variant | no variant | no variant | no variant | no variant | no variant |
| TCGA-TM-A84I-01 | chr2 | C | T | Missense_Mutation | p.R132H | 0.5 |
| TCGA-06-A7TL-01 | chr2 | C | T | Missense_Mutation | p.R132H | 0.26 |
| TCGA-19-A6J5-01 | chr2 | C | T | Missense_Mutation | p.R132H | 0.54 |
| TCGA-19-A6J4-01 | no variant | no variant | no variant | no variant | no variant | no variant |
| TCGA-06-1806-01 | no variant | no variant | no variant | no variant | no variant | no variant |
| TCGA-76-6283-01 | no variant | no variant | no variant | no variant | no variant | no variant |
| TCGA-4W-AA9T-01 | no variant | no variant | no variant | no variant | no variant | no variant |
| TCGA-RR-A6KC-01 | no variant | no variant | no variant | no variant | no variant | no variant |
| TCGA-RR-A6KB-01 | no variant | no variant | no variant | no variant | no variant | no variant |
| TCGA-RR-A6KA-01 | no variant | no variant | no variant | no variant | no variant | no variant |
| TCGA-06-A6S1-01 | no variant | no variant | no variant | no variant | no variant | no variant |
| TCGA-06-A7TK-01 | no variant | no variant | no variant | no variant | no variant | no variant |
| TCGA-76-6660-01 | no variant | no variant | no variant | no variant | no variant | no variant |
| TCGA-06-A5U1-01 | no variant | no variant | no variant | no variant | no variant | no variant |
| TCGA-OX-A56R-01 | no variant | no variant | no variant | no variant | no variant | no variant |
| TCGA-4W-AA9S-01 | no variant | no variant | no variant | no variant | no variant | no variant |
| TCGA-06-A6S0-01 | no variant | no variant | no variant | no variant | no variant | no variant |
| TCGA-19-A60I-01 | no variant | no variant | no variant | no variant | no variant | no variant |
| TCGA-4W-AA9R-01 | no variant | no variant | no variant | no variant | no variant | no variant |
| TCGA-06-A5U0-01 | no variant | no variant | no variant | no variant | no variant | no variant |
| TCGA-26-A7UX-01 | no variant | no variant | no variant | no variant | no variant | no variant |
| TCGA-06-0119-01 | no variant | no variant | no variant | no variant | no variant | no variant |
| TCGA-06-0140-01 | no variant | no variant | no variant | no variant | no variant | no variant |
| TCGA-06-0142-01 | no variant | no variant | no variant | no variant | no variant | no variant |
| TCGA-06-0151-01 | no variant | no variant | no variant | no variant | no variant | no variant |
| TCGA-06-0165-01 | no variant | no variant | no variant | no variant | no variant | no variant |
| TCGA-06-0167-01 | no variant | no variant | no variant | no variant | no variant | no variant |
| TCGA-06-0240-01 | no variant | no variant | no variant | no variant | no variant | no variant |
| TCGA-76-4932-01 | no variant | no variant | no variant | no variant | no variant | no variant |
| TCGA-02-2470-01 | no variant | no variant | no variant | no variant | no variant | no variant |
| TCGA-32-2495-01 | no variant | no variant | no variant | no variant | no variant | no variant |
| TCGA-32-4209-01 | no variant | no variant | no variant | no variant | no variant | no variant |
| TCGA-02-0007-01 |  |  |  |  |  |  |
| TCGA-02-0010-01 |  |  |  |  |  |  |
| TCGA-02-0024-01 |  |  |  |  |  |  |
| TCGA-02-0028-01 |  |  |  |  |  |  |
| TCGA-02-0043-01 |  |  |  |  |  |  |
| TCGA-02-0057-01 |  |  |  |  |  |  |
| TCGA-02-0058-01 |  |  |  |  |  |  |
| TCGA-02-0083-01 |  |  |  |  |  |  |
| TCGA-02-0089-01 |  |  |  |  |  |  |
| TCGA-02-0102-01 |  |  |  |  |  |  |
| TCGA-02-0107-01 |  |  |  |  |  |  |
| TCGA-02-0113-01 |  |  |  |  |  |  |
| TCGA-02-0114-01 |  |  |  |  |  |  |
| TCGA-02-0115-01 |  |  |  |  |  |  |
| TCGA-02-0116-01 |  |  |  |  |  |  |
| TCGA-02-2466-01 |  |  |  |  |  |  |
| TCGA-14-0736-02 |  |  |  |  |  |  |
| TCGA-27-2526-01 | no variant | no variant | no variant | no variant | no variant | no variant |
| TCGA-27-1831-01 | no variant | no variant | no variant | no variant | no variant | no variant |
| TCGA-12-3652-01 | no variant | no variant | no variant | no variant | no variant | no variant |
| TCGA-06-0178-01 |  |  |  |  |  |  |
| TCGA-28-5213-01 | no variant | no variant | no variant | no variant | no variant | no variant |
| TCGA-06-0219-01 | no variant | no variant | no variant | no variant | no variant | no variant |
| TCGA-14-1829-01 | no variant | no variant | no variant | no variant | no variant | no variant |
| TCGA-76-4929-01 | no variant | no variant | no variant | no variant | no variant | no variant |
| TCGA-41-2572-01 | no variant | no variant | no variant | no variant | no variant | no variant |
| TCGA-27-1832-01 | no variant | no variant | no variant | no variant | no variant | no variant |
| TCGA-14-1402-02 |  |  |  |  |  |  |
| TCGA-12-5295-01 | no variant | no variant | no variant | no variant | no variant | no variant |
| TCGA-06-0749-01 | no variant | no variant | no variant | no variant | no variant | no variant |
| TCGA-06-5411-01 | no variant | no variant | no variant | no variant | no variant | no variant |
| TCGA-06-5416-01 | no variant | no variant | no variant | no variant | no variant | no variant |
| TCGA-27-2524-01 | no variant | no variant | no variant | no variant | no variant | no variant |
| TCGA-12-1597-01 | no variant | no variant | no variant | no variant | no variant | no variant |
| TCGA-26-5136-01 | no variant | no variant | no variant | no variant | no variant | no variant |
| TCGA-06-0745-01 | no variant | no variant | no variant | no variant | no variant | no variant |
| TCGA-06-2569-01 | no variant | no variant | no variant | no variant | no variant | no variant |
| TCGA-32-4213-01 | no variant | no variant | no variant | no variant | no variant | no variant |
| TCGA-27-2519-01 | no variant | no variant | no variant | no variant | no variant | no variant |
| TCGA-28-5220-01 | no variant | no variant | no variant | no variant | no variant | no variant |
| TCGA-26-5132-01 | no variant | no variant | no variant | no variant | no variant | no variant |
| TCGA-14-2554-01 | no variant | no variant | no variant | no variant | no variant | no variant |
| TCGA-06-0168-01 | no variant | no variant | no variant | no variant | no variant | no variant |
| TCGA-06-0750-01 | no variant | no variant | no variant | no variant | no variant | no variant |
| TCGA-06-0132-01 | no variant | no variant | no variant | no variant | no variant | no variant |
| TCGA-06-0744-01 | no variant | no variant | no variant | no variant | no variant | no variant |
| TCGA-06-2567-01 | no variant | no variant | no variant | no variant | no variant | no variant |
| TCGA-27-2521-01 | chr2 | C | T | Missense_Mutation | p.R132H | 0.34 |
| TCGA-14-0817-01 | no variant | no variant | no variant | no variant | no variant | no variant |
| TCGA-06-0743-01 | no variant | no variant | no variant | no variant | no variant | no variant |
| TCGA-28-2499-01 | no variant | no variant | no variant | no variant | no variant | no variant |
| TCGA-76-4931-01 | no variant | no variant | no variant | no variant | no variant | no variant |
| TCGA-02-2486-01 | no variant | no variant | no variant | no variant | no variant | no variant |
| TCGA-12-5299-01 | no variant | no variant | no variant | no variant | no variant | no variant |
| TCGA-06-5859-01 | no variant | no variant | no variant | no variant | no variant | no variant |
| TCGA-06-5418-01 | no variant | no variant | no variant | no variant | no variant | no variant |
| TCGA-08-0386-01 | no variant | no variant | no variant | no variant | no variant | no variant |
| TCGA-06-5415-01 | no variant | no variant | no variant | no variant | no variant | no variant |
| TCGA-12-3650-01 | no variant | no variant | no variant | no variant | no variant | no variant |
| TCGA-14-1823-01 | no variant | no variant | no variant | no variant | no variant | no variant |
| TCGA-06-5413-01 | no variant | no variant | no variant | no variant | no variant | no variant |
| TCGA-76-4925-01 | no variant | no variant | no variant | no variant | no variant | no variant |
| TCGA-06-0184-01 | no variant | no variant | no variant | no variant | no variant | no variant |
| TCGA-19-2629-01 | chr2 | C | T | Missense_Mutation | p.R132H | 0.33 |
| TCGA-41-2571-01 | no variant | no variant | no variant | no variant | no variant | no variant |
| TCGA-14-0787-01 | no variant | no variant | no variant | no variant | no variant | no variant |
| TCGA-14-0790-01 | no variant | no variant | no variant | no variant | no variant | no variant |
| TCGA-32-2616-01 | no variant | no variant | no variant | no variant | no variant | no variant |
| TCGA-28-5204-01 | no variant | no variant | no variant | no variant | no variant | no variant |
| TCGA-32-1980-01 | no variant | no variant | no variant | no variant | no variant | no variant |
| TCGA-26-1442-01 | chr2 | C | T | Missense_Mutation | p.R132H | 0.46 |
| TCGA-26-5133-01 | no variant | no variant | no variant | no variant | no variant | no variant |
| TCGA-06-0882-01 | no variant | no variant | no variant | no variant | no variant | no variant |
| TCGA-32-5222-01 | no variant | no variant | no variant | no variant | no variant | no variant |
| TCGA-19-2625-01 | no variant | no variant | no variant | no variant | no variant | no variant |
| TCGA-28-2513-01 | no variant | no variant | no variant | no variant | no variant | no variant |
| TCGA-06-5412-01 | no variant | no variant | no variant | no variant | no variant | no variant |
| TCGA-06-0138-01 |  |  |  |  |  |  |
| TCGA-19-1389-02 |  |  |  |  |  |  |
| TCGA-06-5408-01 | no variant | no variant | no variant | no variant | no variant | no variant |
| TCGA-06-0649-01 | no variant | no variant | no variant | no variant | no variant | no variant |
| TCGA-02-2483-01 | chr2 | C | T | Missense_Mutation | p.R132H | 0.36 |
| TCGA-06-1804-01 | no variant | no variant | no variant | no variant | no variant | no variant |
| TCGA-28-5208-01 | no variant | no variant | no variant | no variant | no variant | no variant |
| TCGA-06-0238-01 | no variant | no variant | no variant | no variant | no variant | no variant |
| TCGA-15-0742-01 | no variant | no variant | no variant | no variant | no variant | no variant |
| TCGA-06-0156-01 |  |  |  |  |  |  |
| TCGA-19-2624-01 | no variant | no variant | no variant | no variant | no variant | no variant |
| TCGA-28-1747-01 | no variant | no variant | no variant | no variant | no variant | no variant |
| TCGA-32-1982-01 | no variant | no variant | no variant | no variant | no variant | no variant |
| TCGA-06-0141-01 | no variant | no variant | no variant | no variant | no variant | no variant |
| TCGA-27-2528-01 | no variant | no variant | no variant | no variant | no variant | no variant |
| TCGA-06-5410-01 | no variant | no variant | no variant | no variant | no variant | no variant |
| TCGA-76-4927-01 | no variant | no variant | no variant | no variant | no variant | no variant |
| TCGA-02-0055-01 | no variant | no variant | no variant | no variant | no variant | no variant |
| TCGA-14-0781-01 | no variant | no variant | no variant | no variant | no variant | no variant |
| TCGA-06-2558-01 | no variant | no variant | no variant | no variant | no variant | no variant |
| TCGA-32-2632-01 | no variant | no variant | no variant | no variant | no variant | no variant |
| TCGA-28-5216-01 | no variant | no variant | no variant | no variant | no variant | no variant |
| TCGA-14-1825-01 | no variant | no variant | no variant | no variant | no variant | no variant |
| TCGA-12-0821-01 | no variant | no variant | no variant | no variant | no variant | no variant |
| TCGA-32-1970-01 | no variant | no variant | no variant | no variant | no variant | no variant |
| TCGA-76-4926-01 | no variant | no variant | no variant | no variant | no variant | no variant |
| TCGA-12-0616-01 | no variant | no variant | no variant | no variant | no variant | no variant |
| TCGA-32-2634-01 | no variant | no variant | no variant | no variant | no variant | no variant |
| TCGA-76-4928-01 | no variant | no variant | no variant | no variant | no variant | no variant |
| TCGA-06-0130-01 | no variant | no variant | no variant | no variant | no variant | no variant |
| TCGA-19-1787-01 | no variant | no variant | no variant | no variant | no variant | no variant |
| TCGA-26-5135-01 | no variant | no variant | no variant | no variant | no variant | no variant |
| TCGA-32-2638-01 | no variant | no variant | no variant | no variant | no variant | no variant |
| TCGA-06-0646-01 | no variant | no variant | no variant | no variant | no variant | no variant |
| TCGA-06-0211-01 | no variant | no variant | no variant | no variant | no variant | no variant |
| TCGA-06-0644-01 | no variant | no variant | no variant | no variant | no variant | no variant |
| TCGA-27-2523-01 | no variant | no variant | no variant | no variant | no variant | no variant |
| TCGA-28-2514-01 | no variant | no variant | no variant | no variant | no variant | no variant |
| TCGA-19-2620-01 | no variant | no variant | no variant | no variant | no variant | no variant |
| TCGA-27-1835-01 | no variant | no variant | no variant | no variant | no variant | no variant |
| TCGA-27-1837-01 | no variant | no variant | no variant | no variant | no variant | no variant |
| TCGA-26-5139-01 | no variant | no variant | no variant | no variant | no variant | no variant |
| TCGA-28-2509-01 | no variant | no variant | no variant | no variant | no variant | no variant |
| TCGA-41-4097-01 | no variant | no variant | no variant | no variant | no variant | no variant |
| TCGA-06-0878-01 | no variant | no variant | no variant | no variant | no variant | no variant |
| TCGA-06-5417-01 | chr2 | C | T | Missense_Mutation | p.R132H | 0.4 |
| TCGA-26-5134-01 | no variant | no variant | no variant | no variant | no variant | no variant |
| TCGA-14-1034-01 | no variant | no variant | no variant | no variant | no variant | no variant |
| TCGA-19-2619-01 | no variant | no variant | no variant | no variant | no variant | no variant |
| TCGA-28-5209-01 | no variant | no variant | no variant | no variant | no variant | no variant |
| TCGA-06-0157-01 | no variant | no variant | no variant | no variant | no variant | no variant |
| TCGA-12-0618-01 | no variant | no variant | no variant | no variant | no variant | no variant |
| TCGA-06-0187-01 | no variant | no variant | no variant | no variant | no variant | no variant |
| TCGA-02-2485-01 | no variant | no variant | no variant | no variant | no variant | no variant |
| TCGA-06-2563-01 | no variant | no variant | no variant | no variant | no variant | no variant |
| TCGA-12-3653-01 | no variant | no variant | no variant | no variant | no variant | no variant |
| TCGA-41-3915-01 | no variant | no variant | no variant | no variant | no variant | no variant |
| TCGA-14-0871-01 | no variant | no variant | no variant | no variant | no variant | no variant |
| TCGA-27-1834-01 | no variant | no variant | no variant | no variant | no variant | no variant |
| TCGA-06-0645-01 | no variant | no variant | no variant | no variant | no variant | no variant |
| TCGA-06-2561-01 | no variant | no variant | no variant | no variant | no variant | no variant |
| TCGA-16-0846-01 | no variant | no variant | no variant | no variant | no variant | no variant |
| TCGA-06-5856-01 | no variant | no variant | no variant | no variant | no variant | no variant |
| TCGA-06-0686-01 | no variant | no variant | no variant | no variant | no variant | no variant |
| TCGA-06-5414-01 | no variant | no variant | no variant | no variant | no variant | no variant |
| TCGA-28-5218-01 | no variant | no variant | no variant | no variant | no variant | no variant |
| TCGA-06-2559-01 | no variant | no variant | no variant | no variant | no variant | no variant |
| TCGA-06-2562-01 | no variant | no variant | no variant | no variant | no variant | no variant |
| TCGA-06-0210-01 | no variant | no variant | no variant | no variant | no variant | no variant |
| TCGA-06-0174-01 | no variant | no variant | no variant | no variant | no variant | no variant |
| TCGA-32-2615-01 | no variant | no variant | no variant | no variant | no variant | no variant |
| TCGA-06-2570-01 | chr2 | C | T | Missense_Mutation | p.R132H | 0.45 |
| TCGA-14-0789-01 | no variant | no variant | no variant | no variant | no variant | no variant |
| TCGA-41-5651-01 | no variant | no variant | no variant | no variant | no variant | no variant |
| TCGA-06-2557-01 | no variant | no variant | no variant | no variant | no variant | no variant |
| TCGA-06-0158-01 | no variant | no variant | no variant | no variant | no variant | no variant |
| TCGA-16-1045-01 | no variant | no variant | no variant | no variant | no variant | no variant |
| TCGA-19-4065-01 | no variant | no variant | no variant | no variant | no variant | no variant |
| TCGA-28-5215-01 | no variant | no variant | no variant | no variant | no variant | no variant |
| TCGA-06-0747-01 | no variant | no variant | no variant | no variant | no variant | no variant |
| TCGA-28-5207-01 | no variant | no variant | no variant | no variant | no variant | no variant |
| TCGA-06-5858-01 | no variant | no variant | no variant | no variant | no variant | no variant |
| TCGA-06-0125-01 | no variant | no variant | no variant | no variant | no variant | no variant |
| TCGA-28-1753-01 | no variant | no variant | no variant | no variant | no variant | no variant |
| TCGA-06-2564-01 | no variant | no variant | no variant | no variant | no variant | no variant |
| TCGA-12-0619-01 | no variant | no variant | no variant | no variant | no variant | no variant |
| TCGA-06-2565-01 | no variant | no variant | no variant | no variant | no variant | no variant |
| TCGA-02-0047-01 | no variant | no variant | no variant | no variant | no variant | no variant |
| TCGA-06-0129-01 | chr2 | C | T | Missense_Mutation | p.R132H | 0.31 |
| TCGA-27-1830-01 | no variant | no variant | no variant | no variant | no variant | no variant |
| TCGA-06-0190-01 | no variant | no variant | no variant | no variant | no variant | no variant |
| TCGA-19-1390-01 | no variant | no variant | no variant | no variant | no variant | no variant |
| TCGA-19-5960-01 | no variant | no variant | no variant | no variant | no variant | no variant |
| TCGA-06-0221-01 | chr2 | G | C | Missense_Mutation | p.R132G | 0.34 |
| TCGA-06-6701-01 | chr2 | C | T | Missense_Mutation | p.R132H | 0.44 |
| TCGA-06-6389-01 | chr2 | C | T | Missense_Mutation | p.R132H | 0.33 |
| TCGA-06-0128-01 | chr2 | C | T | Missense_Mutation | p.R132H | 0.42 |
| TCGA-14-1456-01 | chr2 | C | T | Missense_Mutation | p.R132H | 0.28 |
| TCGA-14-4157-01 | chr2 | C | T | Missense_Mutation | p.R132H | 0.35 |
| TCGA-32-4208-01 | chr2 | C | T | Missense_Mutation | p.R132H | 0.34 |
| TCGA-19-5947-01 | no variant | no variant | no variant | no variant | no variant | no variant |
| TCGA-06-6697-01 | no variant | no variant | no variant | no variant | no variant | no variant |
| TCGA-81-5911-01 | no variant | no variant | no variant | no variant | no variant | no variant |
| TCGA-26-6173-01 | no variant | no variant | no variant | no variant | no variant | no variant |
| TCGA-76-6662-01 | no variant | no variant | no variant | no variant | no variant | no variant |
| TCGA-76-6663-01 | no variant | no variant | no variant | no variant | no variant | no variant |
| TCGA-14-0862-01 | no variant | no variant | no variant | no variant | no variant | no variant |
| TCGA-14-1450-01 | no variant | no variant | no variant | no variant | no variant | no variant |
| TCGA-14-1043-01 | no variant | no variant | no variant | no variant | no variant | no variant |
| TCGA-74-6575-01 | no variant | no variant | no variant | no variant | no variant | no variant |
| TCGA-41-6646-01 | no variant | no variant | no variant | no variant | no variant | no variant |
| TCGA-74-6581-01 | no variant | no variant | no variant | no variant | no variant | no variant |
| TCGA-28-5214-01 | no variant | no variant | no variant | no variant | no variant | no variant |
| TCGA-76-6282-01 | no variant | no variant | no variant | no variant | no variant | no variant |
| TCGA-76-6192-01 | no variant | no variant | no variant | no variant | no variant | no variant |
| TCGA-06-6390-01 | no variant | no variant | no variant | no variant | no variant | no variant |
| TCGA-28-6450-01 | no variant | no variant | no variant | no variant | no variant | no variant |
| TCGA-28-5211-01 | no variant | no variant | no variant | no variant | no variant | no variant |
| TCGA-76-6286-01 | no variant | no variant | no variant | no variant | no variant | no variant |
| TCGA-06-0650-01 | no variant | no variant | no variant | no variant | no variant | no variant |
| TCGA-19-5952-01 | no variant | no variant | no variant | no variant | no variant | no variant |
| TCGA-06-6699-01 | no variant | no variant | no variant | no variant | no variant | no variant |
| TCGA-06-0152-01 | no variant | no variant | no variant | no variant | no variant | no variant |
| TCGA-14-0736-01 | no variant | no variant | no variant | no variant | no variant | no variant |
| TCGA-76-6193-01 | no variant | no variant | no variant | no variant | no variant | no variant |
| TCGA-06-6693-01 | no variant | no variant | no variant | no variant | no variant | no variant |
| TCGA-74-6577-01 | no variant | no variant | no variant | no variant | no variant | no variant |
| TCGA-76-6280-01 | no variant | no variant | no variant | no variant | no variant | no variant |
| TCGA-19-5958-01 | no variant | no variant | no variant | no variant | no variant | no variant |
| TCGA-06-6388-01 | no variant | no variant | no variant | no variant | no variant | no variant |
| TCGA-06-0171-01 | no variant | no variant | no variant | no variant | no variant | no variant |
| TCGA-19-5953-01 | no variant | no variant | no variant | no variant | no variant | no variant |
| TCGA-06-6698-01 | no variant | no variant | no variant | no variant | no variant | no variant |
| TCGA-87-5896-01 | no variant | no variant | no variant | no variant | no variant | no variant |
| TCGA-74-6573-01 | no variant | no variant | no variant | no variant | no variant | no variant |
| TCGA-19-5950-01 | no variant | no variant | no variant | no variant | no variant | no variant |
| TCGA-06-6695-01 | no variant | no variant | no variant | no variant | no variant | no variant |
| TCGA-74-6584-01 | no variant | no variant | no variant | no variant | no variant | no variant |
| TCGA-14-1395-01 | no variant | no variant | no variant | no variant | no variant | no variant |
| TCGA-76-6661-01 | no variant | no variant | no variant | no variant | no variant | no variant |
| TCGA-74-6578-01 | no variant | no variant | no variant | no variant | no variant | no variant |
| TCGA-19-5959-01 | no variant | no variant | no variant | no variant | no variant | no variant |
| TCGA-76-6656-01 | no variant | no variant | no variant | no variant | no variant | no variant |
| TCGA-32-1979-01 | no variant | no variant | no variant | no variant | no variant | no variant |
| TCGA-81-5910-01 | no variant | no variant | no variant | no variant | no variant | no variant |
| TCGA-19-5955-01 | no variant | no variant | no variant | no variant | no variant | no variant |
| TCGA-76-6664-01 | no variant | no variant | no variant | no variant | no variant | no variant |
| TCGA-76-6657-01 | no variant | no variant | no variant | no variant | no variant | no variant |
| TCGA-76-6191-01 | no variant | no variant | no variant | no variant | no variant | no variant |
| TCGA-06-6700-01 | no variant | no variant | no variant | no variant | no variant | no variant |
| TCGA-19-5956-01 | no variant | no variant | no variant | no variant | no variant | no variant |
| TCGA-26-6174-01 | no variant | no variant | no variant | no variant | no variant | no variant |
| TCGA-19-5954-01 | no variant | no variant | no variant | no variant | no variant | no variant |
| TCGA-19-5951-01 | no variant | no variant | no variant | no variant | no variant | no variant |
| TCGA-06-6694-01 | no variant | no variant | no variant | no variant | no variant | no variant |
| TCGA-76-6285-01 | no variant | no variant | no variant | no variant | no variant | no variant |
| TCGA-06-6391-01 | no variant | no variant | no variant | no variant | no variant | no variant |
| TCGA-12-5301-01 | no variant | no variant | no variant | no variant | no variant | no variant |
| TCGA-28-5219-01 | no variant | no variant | no variant | no variant | no variant | no variant |
| TCGA-76-4935-01 | no variant | no variant | no variant | no variant | no variant | no variant |
| TCGA-76-4934-01 | no variant | no variant | no variant | no variant | no variant | no variant |
| TCGA-14-0740-01 | no variant | no variant | no variant | no variant | no variant | no variant |
| TCGA-02-0003-01 | no variant | no variant | no variant | no variant | no variant | no variant |
| TCGA-02-0033-01 | no variant | no variant | no variant | no variant | no variant | no variant |
| TCGA-06-0122-01 | no variant | no variant | no variant | no variant | no variant | no variant |
| TCGA-06-0124-01 | no variant | no variant | no variant | no variant | no variant | no variant |
| TCGA-06-0126-01 | no variant | no variant | no variant | no variant | no variant | no variant |
| TCGA-06-0137-01 | no variant | no variant | no variant | no variant | no variant | no variant |
| TCGA-06-0139-01 | no variant | no variant | no variant | no variant | no variant | no variant |
| TCGA-06-0145-01 | no variant | no variant | no variant | no variant | no variant | no variant |
| TCGA-06-0154-01 | no variant | no variant | no variant | no variant | no variant | no variant |
| TCGA-06-0155-01 | no variant | no variant | no variant | no variant | no variant | no variant |
| TCGA-06-0166-01 | no variant | no variant | no variant | no variant | no variant | no variant |
| TCGA-06-0169-01 | no variant | no variant | no variant | no variant | no variant | no variant |
| TCGA-06-0173-01 | no variant | no variant | no variant | no variant | no variant | no variant |
| TCGA-06-0185-01 | no variant | no variant | no variant | no variant | no variant | no variant |
| TCGA-06-0188-01 | no variant | no variant | no variant | no variant | no variant | no variant |
| TCGA-06-0189-01 | no variant | no variant | no variant | no variant | no variant | no variant |
| TCGA-06-0192-01 | no variant | no variant | no variant | no variant | no variant | no variant |
| TCGA-06-0195-01 | no variant | no variant | no variant | no variant | no variant | no variant |
| TCGA-06-0209-01 | no variant | no variant | no variant | no variant | no variant | no variant |
| TCGA-06-0213-01 | no variant | no variant | no variant | no variant | no variant | no variant |
| TCGA-06-0214-01 | no variant | no variant | no variant | no variant | no variant | no variant |
| TCGA-06-0216-01 | no variant | no variant | no variant | no variant | no variant | no variant |
| TCGA-06-0237-01 | no variant | no variant | no variant | no variant | no variant | no variant |
| TCGA-06-0241-01 | no variant | no variant | no variant | no variant | no variant | no variant |
| TCGA-06-0648-01 | no variant | no variant | no variant | no variant | no variant | no variant |
| TCGA-06-0875-01 | no variant | no variant | no variant | no variant | no variant | no variant |
| TCGA-06-0876-01 | no variant | no variant | no variant | no variant | no variant | no variant |
| TCGA-06-0877-01 | no variant | no variant | no variant | no variant | no variant | no variant |
| TCGA-06-0879-01 | no variant | no variant | no variant | no variant | no variant | no variant |
| TCGA-06-0881-01 | no variant | no variant | no variant | no variant | no variant | no variant |
| TCGA-06-0939-01 | no variant | no variant | no variant | no variant | no variant | no variant |
| TCGA-12-0615-01 | no variant | no variant | no variant | no variant | no variant | no variant |
| TCGA-12-0688-01 | no variant | no variant | no variant | no variant | no variant | no variant |
| TCGA-12-0692-01 | no variant | no variant | no variant | no variant | no variant | no variant |
| TCGA-12-3649-01 | no variant | no variant | no variant | no variant | no variant | no variant |
| TCGA-14-0786-01 | no variant | no variant | no variant | no variant | no variant | no variant |
| TCGA-14-0813-01 | no variant | no variant | no variant | no variant | no variant | no variant |
| TCGA-14-3476-01 | no variant | no variant | no variant | no variant | no variant | no variant |
| TCGA-16-0848-01 | no variant | no variant | no variant | no variant | no variant | no variant |
| TCGA-16-0861-01 | no variant | no variant | no variant | no variant | no variant | no variant |
| TCGA-19-1790-01 | no variant | no variant | no variant | no variant | no variant | no variant |
| TCGA-19-2623-01 | no variant | no variant | no variant | no variant | no variant | no variant |
| TCGA-19-2631-01 | no variant | no variant | no variant | no variant | no variant | no variant |
| TCGA-19-4068-01 | no variant | no variant | no variant | no variant | no variant | no variant |
| TCGA-26-1439-01 | no variant | no variant | no variant | no variant | no variant | no variant |
| TCGA-27-1833-01 | no variant | no variant | no variant | no variant | no variant | no variant |
| TCGA-27-1836-01 | no variant | no variant | no variant | no variant | no variant | no variant |
| TCGA-27-1838-01 | no variant | no variant | no variant | no variant | no variant | no variant |
| TCGA-27-2518-01 | no variant | no variant | no variant | no variant | no variant | no variant |
| TCGA-27-2527-01 | no variant | no variant | no variant | no variant | no variant | no variant |
| TCGA-28-2502-01 | no variant | no variant | no variant | no variant | no variant | no variant |
| TCGA-32-1977-01 | no variant | no variant | no variant | no variant | no variant | no variant |
| TCGA-32-1986-01 | no variant | no variant | no variant | no variant | no variant | no variant |
| TCGA-32-1991-01 | no variant | no variant | no variant | no variant | no variant | no variant |
| TCGA-32-2491-01 | no variant | no variant | no variant | no variant | no variant | no variant |
| TCGA-32-2494-01 | no variant | no variant | no variant | no variant | no variant | no variant |
| TCGA-32-4210-01 | no variant | no variant | no variant | no variant | no variant | no variant |
| TCGA-32-4211-01 | no variant | no variant | no variant | no variant | no variant | no variant |
| TCGA-32-4719-01 | no variant | no variant | no variant | no variant | no variant | no variant |
| TCGA-41-2573-01 | no variant | no variant | no variant | no variant | no variant | no variant |
| TCGA-41-2575-01 | no variant | no variant | no variant | no variant | no variant | no variant |
| TCGA-41-3392-01 | no variant | no variant | no variant | no variant | no variant | no variant |
| TCGA-41-3393-01 | no variant | no variant | no variant | no variant | no variant | no variant |
